# Low GNG12 Expression Predicts Adverse Outcomes: A Potential Therapeutic Target for Osteosarcoma

**DOI:** 10.3389/fimmu.2021.758845

**Published:** 2021-10-06

**Authors:** Jinghong Yuan, Zhao Yuan, Aifang Ye, Tianlong Wu, Jingyu Jia, Jia Guo, Jian Zhang, Tao Li, Xigao Cheng

**Affiliations:** ^1^ Department of Orthopaedics, The Second Affiliated Hospital of Nanchang University, Nanchang, China; ^2^ Clinical Research Center, The Second Affiliated Hospital of Nanchang University, Nanchang, China; ^3^ Department of Otorhinolaryngology, Jiangxi Provincial Children’s Hospital, Nanchang, China; ^4^ Institute of Orthopaedics of Jiangxi Province, Nanchang, China; ^5^ Institute of Minimally Invasive Orthopaedics of Nanchang University, Nanchang University, Nanchang, China; ^6^ Department of Orthopaedics, Jiangxi Provincial People’s Hospital Affiliated to Nanchang University, Nanchang, China

**Keywords:** osteosarcoma, GNG12, biomarker, therapeutic target, prognosis

## Abstract

**Background:**

G protein subunit gamma 12 (GNG12) is observed in some types of cancer, but its role in osteosarcoma is unknown. This study hypothesized that GNG12 may be a potential biomarker and therapeutic target. We aimed to identify an association between GNG12 and osteosarcoma based on the Gene Expression Omnibus and the Therapeutically Applicable Research to Generate Effective Treatments (TARGET) databases.

**Methods:**

Osteosarcoma samples in GSE42352 and TARGET database were selected as the test cohorts. As the external validation cohort, 78 osteosarcoma specimens from The Second Affiliated Hospital of Nanchang University were collected. Patients with osteosarcoma were divided into high and low GNG12 mRNA-expression groups; differentially expressed genes were identified as GNG12-related genes. The biological function of GNG12 was annotated using Gene Ontology, Kyoto Encyclopedia of Genes and Genomes, gene set enrichment analysis, and immune infiltration analysis. Gene expression correlation analysis and competing endogenous RNA regulatory network construction were used to determine potential biological regulatory relationships of GNG12. Overall survival, Kaplan–Meier analysis, and log-rank tests were calculated to determine GNG12 reliability in predicting survival prognosis.

**Results:**

GNG12 expression decreased in osteosarcoma samples. GNG12 was a highly effective biomarker for osteosarcoma [area under the receiver operating characteristic (ROC) curve (AUC) = 0.920], and the results of our Kaplan–Meier analysis indicated that overall survival and progression-free survival differed significantly between low and high GNG-expression group (p < 0.05). Functional analyses indicated that GNG12 may promote osteosarcoma through regulating the endoplasmic reticulum. Expression correlation analysis and competing endogenous RNA network construction showed that HOTTIP/miR-27a-3p may regulate GNG12 expression. Furthermore, the subunit suppresses adaptive immunity *via* inhibiting M1 and M2 macrophage infiltration. GNG12 was inhibited in metastatic osteosarcoma compared with non-metastatic osteosarcoma, and its expression predicted survival of patients (1, 3, and 5-year AUCs were 0.961, 0.826, and 0.808, respectively).

**Conclusion:**

This study identified GNG12 as a potential biomarker for osteosarcoma prognosis, highlighting its potential as an immunotherapy target.

## 1 Introduction

Osteosarcoma is among the most common primary solid malignant bone tumors in adolescents and young adults. Usually originating in the metaphyses of long bones, the cancer is characterized by heterogeneous presentation, high mortality, and an annual incidence of 8–11 million among 15–19-year-olds (1). After comprehensive treatment with methods such as radiotherapy and chemotherapy, the 5-year survival rate is 65%–70% (2, 3), but these improvements have been mainly limited to patients with non-metastatic disease. Moreover, the progress of research aiming to raise osteosarcoma survival has stalled over the past 30 years (4). Therefore, it is critical to determine reliable predictors related to osteosarcoma metastasis and prognosis and to make available novel targets for therapy and prognosis prediction. However, due to the complex molecular mechanisms of osteosarcoma, the predictive capability of traditional clinical information is limited. Thus, it is crucial to find novel prognostic biomarkers to predict survival and metastases more accurately in osteosarcoma. Although biomarkers such as FAT10 and MYC have been associated with osteosarcoma in recent studies, their reliability requires further investigation (5, 6).

G protein subunit gamma 12 (GNG12) is a protein-coding gene located on chromosome 1, first reported in 1995 (7). In the GeneCards database (www.genecards.org), related pathways include “Translation Translation regulation by Alpha-1 adrenergic receptors” and “Sweet Taste Signaling”. Gene Ontology (GO) annotations related to this gene include obsolete signal transducer activity and phosphate ion binding. Previous research (8) suggested that GNG12 knockdown in BV-2 cells increased nitric oxide levels and tumor necrosis factor alpha (TNF-α) expression in response to lipopolysaccharide (LPS) stimulation, indicating that GNG12 was an essential negative regulator of inflammation. Transforming growth factor beta (TGF-β)-induced chondrogenic differentiation of mesenchymal stem cells is promoted when C-type natriuretic peptide/natriuretic peptide receptor-B reduces GNG12 expression (9). The first association of GNG12 with cancer was a study showing that low GNG12 expression increases the proliferation of endometrial cancer (10). Subsequently, HOXA13 upregulation was found to be a promoter of lung squamous cancer progression through reducing GNG12 expression (11). Although these studies suggest an important role for GNG12 in cancer progression, we currently know little about the underlying mechanisms and function of this protein in osteosarcoma progression and immunology.

This study thus aimed to determine the role of GNG12 in patients with osteosarcoma. We obtained two test cohort from the Gene Expression Omnibus (GEO) database and the Therapeutically Applicable Research to Generate Effective Treatments (TARGET) database. To clarify biological function, we compared expression matrices of high and low GNG12-expression groups to identify potential GNG12-related genes. These genes were screened using functional enrichment analyses, gene set enrichment analysis (GSEA) regulation networks, immune infiltration analysis, and pan-cancer analysis. We also collected osteosarcoma samples to evaluate GNG12 predictive ability on survival and prognosis, using immunohistochemistry, histochemistry score (H-Score) analysis, and receiver operating characteristic (ROC) analysis.

Our results demonstrated that low GNG12 expression is a potential biomarker of osteosarcoma, linked to poor prognosis. GSEA revealed that GNG12 expression was associated with “extracellular matrix organization”, “core matrisome”, “cytoplasmic ribosomal proteins”, “cell adhesion molecules cams”, “class A 1 rhodopsin-like receptors”, “GPCR ligand binding”, and “diseases of metabolism”, and “Matrisome”. Immune infiltration analysis demonstrated that GNG12 regulated the proportions of macrophages (M0, M1, and M2) and mast cells to influence the tumor microenvironment. Finally, we confirmed the feasibility of GNG12 as a biomarker in our collected osteosarcoma samples.

## 2 Materials and Methods

### 2.1 Patients and Identification of Differentially Expressed Genes

The GEO (http://www.ncbi.nlm.nih.gov/geo) database is a free public gene-expression data repository containing microarray and high-throughput sequencing data. Gene-expression datasets from GSE42352 (12, 13), including 103 osteosarcoma cases and 15 normal controls, were collected from GEO as the test cohort. Using the median value of GNG12 expression as the cutoff, the 103 patients were divided into high (n = 52) and low (n = 51) GNG12 mRNA expression groups. Significant differentially expressed genes (DEGs) between high and low GNG12-expression groups were screened with the limma package (http://www.bioconductor.org/packages/release/bioc/html/limma.html) in R version 3.6.3 (http://www.R-project.org/) (14). Genes were considered differentially expressed with an adjusted p < 0.5 and |log fold change| (|logFC|) ≥ 1. Heat maps were constructed using R package “pheatmap”, version 1.0.12 (https://cran.rproject.org/web/packages/pheatmap/index.html), and volcano plots were generated with “ggplot2” version 3.3.3 (https://cran.r-project.org/web/packages/ggplot2/index.html). In addition, gene expression profiles and clinical data of osteosarcoma cases were collected from TARGET (https://ocg.cancer.gov/programs/target). The TARGET cohort (n = 101) were divided into high/low groups using the same cutoff as the GEO cohort (high, 51 cases; low, 50 cases). The same method as described above was used to determine DEGs between TARGET high/low groups. Common DEGs between the GSE42352 and TARGET datasets were represented with a Venn diagram made in ggplot2.

The Second Affiliated Hospital of Nanchang University provided 78 osteosarcoma specimens from January 2012 to December 2016 as an external validation cohort. Samples were provided as formalin fixed paraffin-embedded blocks.

This research was approved by the Ethics Committee of The Second Affiliated Hospital of Nanchang University [Review (2020) No. (086)]. All participants provided informed consent.

### 2.2 Functional Enrichment Analysis

Functional enrichment analyses (Gene Ontology, GO; https://www.geneontology.org and Kyoto Encyclopedia of Genes and Genomes, KEGG; https://www.genome.jp/kegg/) were performed on DEGs between high and low GNG12-expression groups in the test cohort, using clusterProfiler version 3.14.3 (http://www.bioconductor.org/packages/release/bioc/html/clusterProfiler.html). The results were visualized with the “ggplot2” package (https://cran.r-project.org/web/packages/ggplot2/index.html). Xiantao Academic online (https://www.xiantao.love) was used to generate a diagram of the functional enrichment network analysis.

Significant differences in function and pathways between high/low GNG12-expression groups were determined using GSEA in clusterProfiler and Xiantao Academic online. For each analysis, gene set permutations were performed 1,000 times to obtain a normalized enrichment score (NES). Enrichment was considered significant with adjusted p < 0.05, false discovery rate (FDR) q < 0.25, and |NES| > 1. The selected reference gene set was c2.cp.v7.2.symbols.gmt (Curated), and results were visualized in ggplot2.

### 2.3 Protein–Protein Interaction Network Construction and Hub-Gene Extraction

A protein–protein interaction (PPI) network of DEGs was constructed using Metascape online tools (https://metascape.org) with the following parameters: min network size = 3 and max network size = 500. Crucial proteins in this PPI network were screened using the Molecular Complex Detection (MCODE, http://apps.cytoscape.org/apps/mcode), a plug-in of Cytoscape version 3.7.2 (https://cytoscape.org/).

### 2.4 Immune Infiltration Analysis

CIBERSORT (https://cibersort.stanford.edu/) is an algorithm developed to characterize the cellular composition of complex tissues through gene expression profiles compared with a signature matrix (LM22). LM22 comprises 547 genes that define 22 immunization cell subtypes. This analysis was used to calculate infiltration abundance of 22 immune cells (T cells, B cells, plasma cells, natural killer cells, myeloid subgroups) in 103 osteosarcoma samples across high and low GNG12-expression groups. We also analyzed any correlations between the two GNG12 groups in terms of expression distribution among the 22 infiltrating immune cells; the correlation heat map was then plotted. Red represents a positive correlation, and blue represents a negative correlation, with darker colors and values closer to 1 indicating stronger correlations.

### 2.5 GNG12 Expression Analysis

The UCSC XENA platform (https://xenabrowser.net/datapages/) is a pan-cancer HTSeq-TPM database of TCGA and GTEx, processed uniformly by Toil. Based on XENA data, differential GNG12 mRNA expression between tumor and normal tissues was determined in R and visualized with ggplot2. Spearman’s correlations were calculated between GNG12 expression in the TARGET cohort with the expression of nine DEGs (GREM1, CAV1, PTGDS, PRB2, PRB1, NDUFB9, AMBN, FAT3, and IBSP) common across GSE42352 and TARGET datasets. Again, the analysis was performed in R and the results visualized in ggplot2.

#### 2.5.1 Construction of Competing Endogenous RNA Network

A differential long noncoding RNA (lncRNA) expression matrix was obtained from TARGET using the limma package. The miRcode database (http://www.mircode.org/) was used to predict a series of highly conserved microRNAs (miRNAs) linked to differentially expressed lncRNA. The miRNA target genes were predicted using miRDB (http://mirdb.org/), miRTarBase (http://miRTarBase.cuhk.edu.cn/), and TargetScan (http://www.targetscan.org/mamm_31/) databases. Predicted mRNAs were associated with the six GNG12-related genes (GREM1, CAV1, NDUFB9, AMBN, FAT3, and IBSP) to establish a competing endogenous RNA (ceRNA) network.

### 2.6 Survival Analysis

Kaplan–Meier (KM) analyses of the TARGET database were performed using R packages survival (https://www.rdocumentation.org/packages/survival/versions/2.42-3) and survminer (https://cran.rstudio.com/web/packages/survminer/index.html), to determine whether GNG12 expression accurately predicted overall survival time (OS) and progression-free survival (PFS). The timeROC package (https://cran.r-project.org/web/packages/timeROC/index.html) was used to analyze 1-, 3-, and 5-year survival prognosis of GNG12-related lncRNAs, miRNAs, and genes. All outcomes were visualized in ggplot2.

### 2.7 Assessing Diagnostic Performance

An ROC curve was generated using the pROC package (https://cran.r-project.org/web/packages/pROC/). The ordinate was drawn with the true positive rate (sensitivity), and the abscissa was the false positive rate (1 − specificity). This analysis determined whether GNG12 expression can distinguish between 15 standard samples and 103 osteosarcoma samples. Also determined was the cutoff value that produces the highest likelihood ratio for assessing the recognition threshold of GNG12 as a biomarker for distinguishing osteosarcoma from normal tissue.

### 2.8 Immunohistochemical Staining and Evaluation

To further verify the suitability of GNG12 expression for predicting survival, immunohistochemistry was performed on paraffin sections following the standard protocol (GNG12, Ab204757, 1:100). All slides were observed and photographed under a XSP-C204 microscope (CIC). GNG12 expression was assessed using the H-score.

### 2.9 Validation of GNG12 as a Prognostic Predictor of Osteosarcoma

Known survival outcomes of osteosarcoma patients with high and low H-scores were subjected to KM analysis using the R package “survival” and visualized in “surviminer”. The accuracy of GNG12 expression in predicting survival prognosis (1, 3, and 5 years) was verified with timeROC and visualized in ggplot2. An AUC >0.5 directly reflects the prognostic value of the biomarker. Additionally, an AUC closer to 1 and to the (0, 1) point indicates greater authenticity of the prognostic prediction.

### 2.10 Statistical Analysis

Statistics were performed in Microsoft Excel and R version 3.6.3. The log-rank test was used to perform the KM survival analysis. Continuous variables that violated the normality assumption according to the Shapiro–Wilk normality test were analyzed with the Wilcoxon rank sum test. Categorical variables were analyzed with chi-square tests. Significance was set at p < 0.05 (ns, p > 0.05; *p < 0.05; **p < 0.01; ***p < 0.001).

## 3 Results

### 3.1 Identification of DEGs

**Figure 1:**
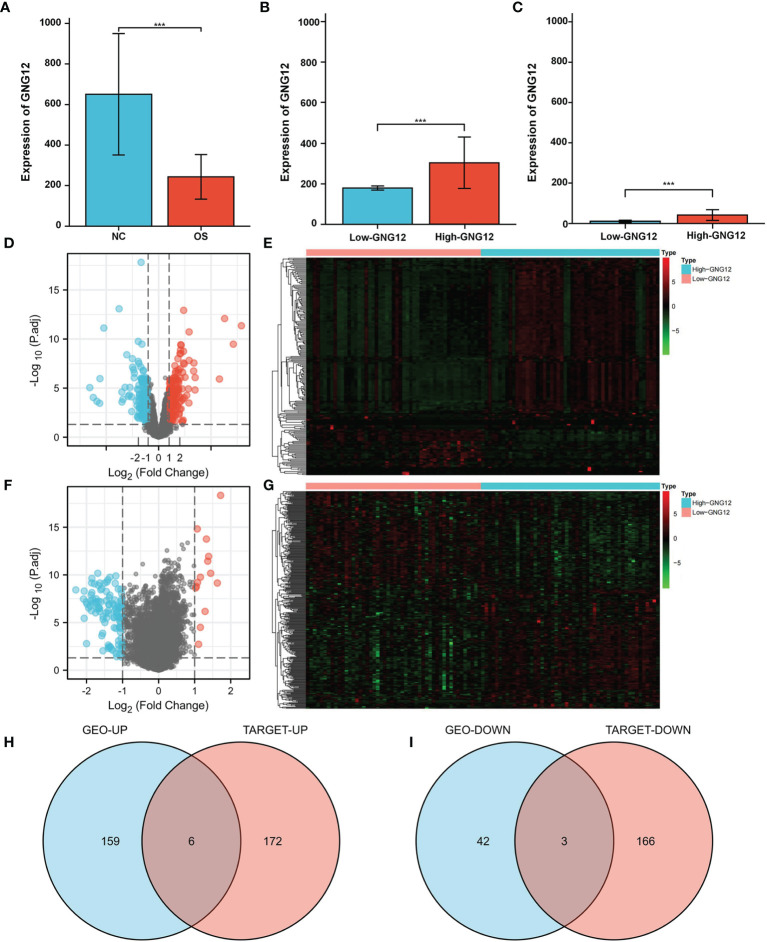
GNG12 and coexpressed gene difference analysis. **(A)** Compared with normal tissues, GNG12 is significantly lower expressed in tumor tissues. **(B)** In GSE42352 cohort, the expression level of GNG12 in low/high GNG12-expression groups. **(C)** In TARGET cohort, the expression level of GNG12 in low/high GNG12 expression groups. **(D)** The median expression of GNG12 in 103 osteosarcoma samples in the GSE42352 data set was divided into high and low expression groups, and the significantly different genes between the two groups were displayed in the form of a volcano graph. **(E)** A heat map showing the significantly different genes between the two groups of the GSE42352 data set. **(F)** GNG12 was used in the median expression values of 101 osteosarcoma samples in the TARGET data set divided into high and low expression groups, showing the significant difference genes between the two groups in the form of volcano maps. **(G)** The TARGET data set shows the significant difference genes between the two groups in the form of a heat map. **(H)** Venn diagram of the intersection of upregulated DEGs in the GEO and TARGET cohort. **(I)** Venn diagram of the intersection of downregulated DEGs in the GEO and TARGET cohort. ***p < 0.001.

GNG12 expression was significantly different between the standard samples (n = 15) and osteosarcoma samples (n = 103) (**Figure 1A**). After screening for DEGs between high and low GNG12-expression groups (**Figure 1B**), we found 210 coexpressed genes (165 upregulated and 45 downregulated, **Supplementary Table 1**) that we have visualized in a volcano plot (**Figure 1D**) and a heat map (**Figure 1E**). Using the same method to divide the TARGET cohort (n = 101) into high/low GNG12-expression groups (**Figure 1C**), we obtained 347 DEGs (178 upregulated and 169 downregulated, **Supplementary Table 2** and **Figures 1F, G**). We then screened out nine common DEGs (six upregulated and three downregulated) across the two DEG sets (**Figures 1H, I**).

### 3.2 Functional Enrichment Analysis and GSEA

**Figure 2:**
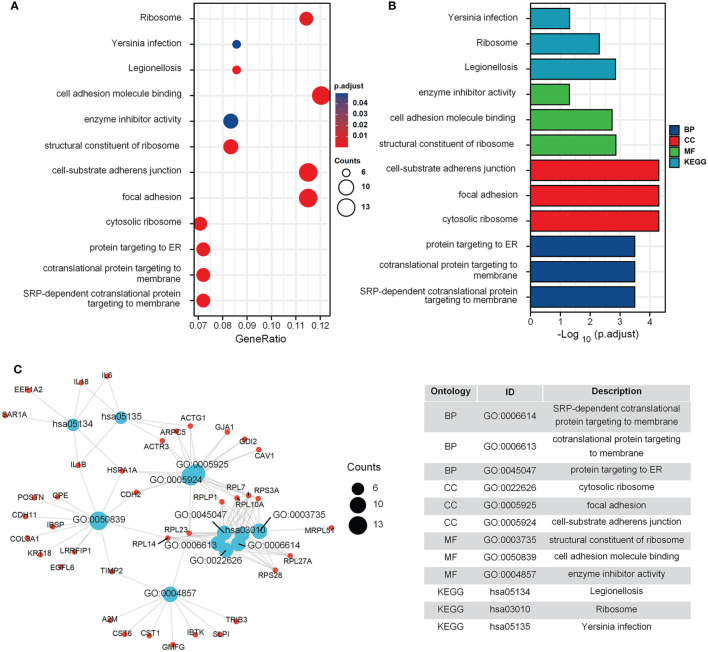
GO and KEGG enrichment analysis in GEO cohort. **(A)** The bubble graph of clusterProfiler package for GO and KEGG enrichment analysis; the bubble size represents the number of gene enrichment, and color represents significance in GSE42352 data set. **(B)** The bar graph of clusterProfiler package for GO and KEGG enrichment analysis; length represents significance in GSE42352 data set. **(C)** A function enrichment network in GSE42352 data set.

**Figure 3:**
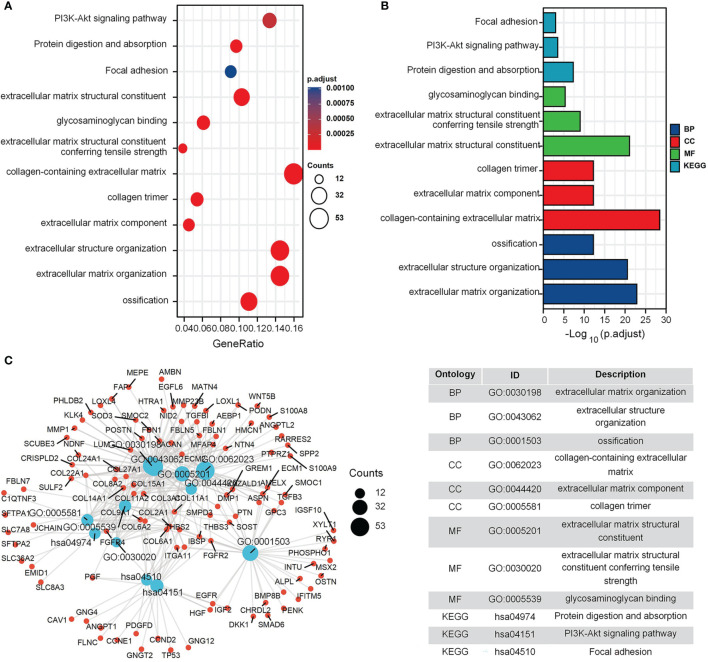
GO and KEGG enrichment analysis in TARGET cohort. **(A)** The bubble graph of clusterProfiler package for GO and KEGG enrichment analysis; the bubble size represents the number of gene enrichment, and color represents significance in TARGET data set. **(B)** The bar graph of clusterProfiler package for GO and KEGG enrichment analysis; length represents significance in TARGET data set. **(C)** A function enrichment network in TARGET data set.

The GO and KEGG enrichment results for the GEO cohort (**Figures 2A, B**) indicated that the following terms were enriched: “protein targeting to endoplasmic reticulum (ER)”, “establishment of protein localization to ER”, “focal adhesion”, and “cell adhesion molecule binding” (**Figure 2C**). For the TARGET cohort, enriched terms were “ossification”, “cartilage development”, “integrin binding”, “PI3K-Akt signaling pathway”, and “Focal adhesion” (**Figure 3**).

**Figure 4:**
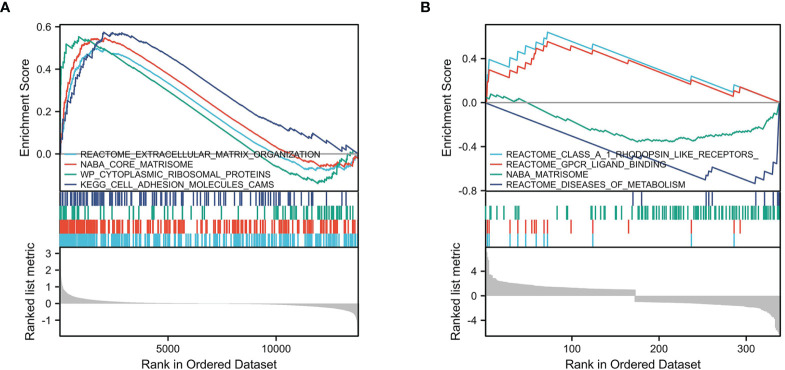
GSEA enrichment analysis. **(A)** GSEA enrichment analysis in GEO cohort. **(B)** GSEA enrichment analysis in TARGET cohort.

We selected msigdb.v7.0.entrez.gmt as the reference gene set for our GSEA. For the GEO cohort, DEGs were involved in “extracellular matrix organization” from the Reactome database, “core matrisome” from NABA, “cytoplasmic ribosomal proteins” from WP, and “cell adhesion molecules cams” from KEGG (**Figure 4A**). For the TARGET cohort, DEGs were involved in “class A 1 rhodopsin like receptors”, “GPCR ligand binding”, and “diseases of metabolism” from the Reactome database, and “Matrisome” from NABA (**Figure 4B**).

### 3.3 Construction of PPI Networks and Hub-Gene Screening

**Figure 5:**
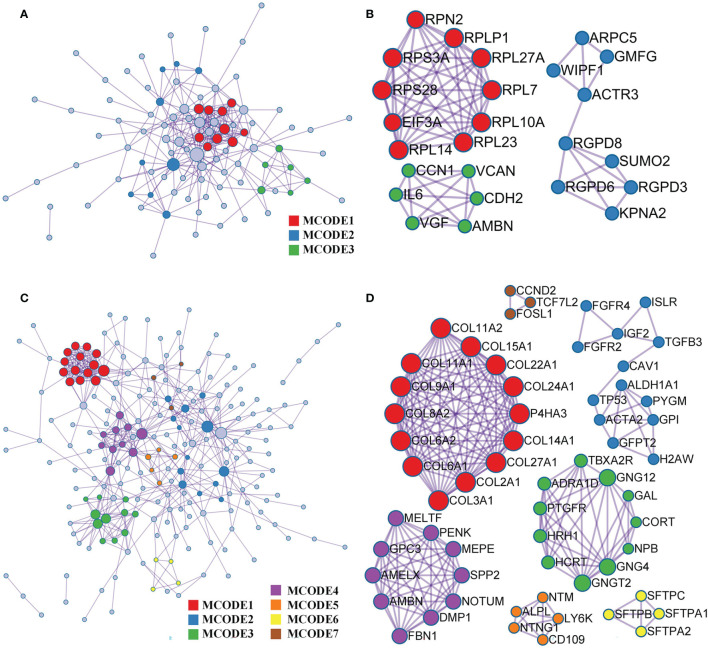
PPI and hub gene clusters network construction. **(A)** A PPI network from GSE42352 data set. **(B)** Three hub gene clusters obtained by the MCODE clustering algorithm from GSE42352 data set. **(C)** A PPI network from TARGET data set. **(D)** Seven hub gene clusters obtained by the MCODE clustering algorithm from TARGET data set.

Based on the Metascape online tool, we constructed two PPI networks of DEGs from the GEO and TARGET cohorts (**Figures 5A, C**). We then screened hub-gene clusters using the MCODE clustering algorithm. In the GEO cohort, screened hub genes were linked in the following networks: MCODE_1 (cytoplasmic translation, cytosolic ribosome, and ribosome; EIF3A, RPL23, RPL14, RPL7, RPL27A, RPS28, RPLP1, RPS3A, RPL10A, and RPN2), MCODE_2 (NLS-bearing protein import into the nucleus, protein import into the nucleus, and import into the nucleus; KPNA2, RGPD6, RGPD3, RGPD8, SUMO2, ACTR3, WIPF1, ARPC5, and GMFG), and MCODE_3 (ER, growth factor activity, and extracellular matrix structural constituent; VCAN, CCN1, AMBN, CDH2, VGF, and IL6; **Figure 5B**). Hub genes of the TARGET cohort were involved in more networks. These were MCODE_1 (extracellular matrix structural constituent conferring tensile strength, collagen trimer, and protein digestion and absorption; COL8A2, COL9A1, COL11A1, COL11A2, COL15A1, COL22A1, COL24A1, P4HA3, COL14A1, COL27A1, COL2A1, COL3A1, COL6A1, and COL6A2), MCODE_2 (*in utero* embryonic development, peptidyl-tyrosine phosphorylation, and peptidyl-tyrosine modification; FGFR4, ISLR, FGF2, IGF2, TGFB3, CAV1, TP53, ACTA2, ALDH1A1, PYGM, GPI, GFPT2, and H2AW), MCODE_3 (G-protein beta-subunit binding, neuropeptide hormone activity, and heterotrimeric G-protein complex; GNG12, GAL, CORT, NPB, GNG4, GNGT2, HCRT, HRH1, PTGFR, ADRA1D, and TBXA2R), MCODE_4 (ER lumen, biomineral tissue development, and biomineralization; MELTF, PENK, MEPE, SPP2, MOTUM, DMP1, FBN1, AMBN, AMELX, and GPC3), MCODE_5 (anchored component of membrane; NTM, ALPL, NTNG1, CD109, and LY6K), MCODE_6 (lamellar body, mutivesicular body and respiratory gaseous exchange by respiratory system; SFTPB, SFTPC, SFTPA1, and SFTPA2), and MCODE_7 (Wnt signaling pathway; CCND2, TCF7L2, and FOSL1; **Figure 5D**).

### 3.4 Correlation Between GNG12 Expression and Immune Infiltration

**Figure 6:**
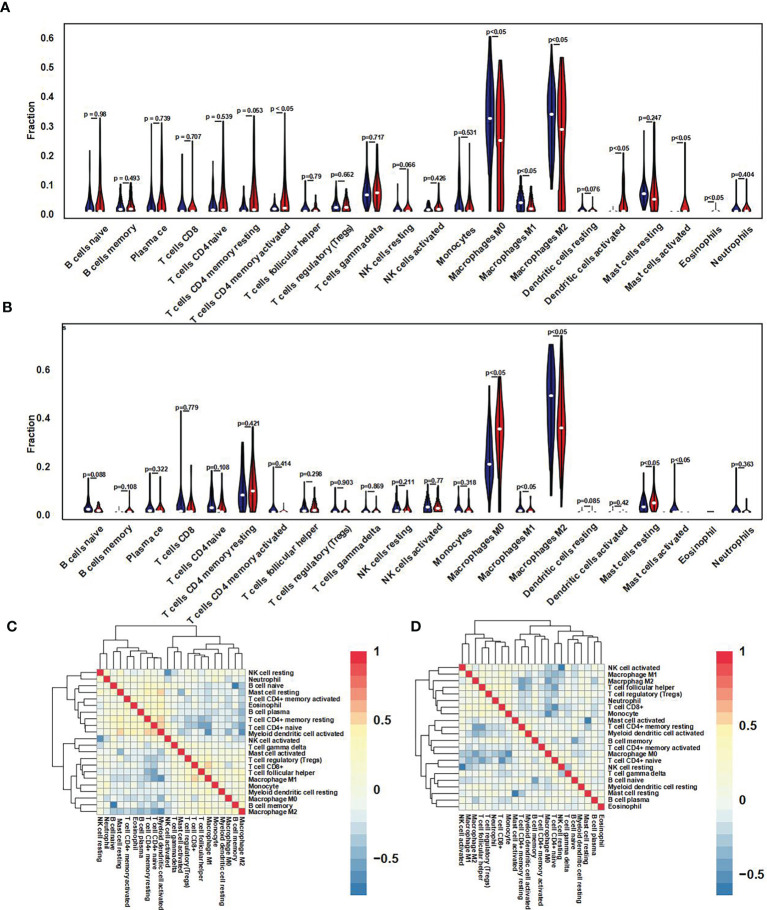
Immune cell infiltration difference and correlation analysis. **(A)** Using the CIBERSORT package, the LM22 algorithm was used to calculate the difference in expression of 22 immune cells in the GNG12 high and low expression groups in the 103 osteosarcoma samples in the GEO set. Blue represents the low expression group, and red represents the high expression group. **(B)** Violin diagram of the difference in expression of 22 immune cells infiltration between the high and low expression groups of GNG12 in 101 osteosarcoma samples in the TARGET set. Blue represents the low expression group; red represents the high expression group. **(C)** Correlation heat map of 22 immune cells in the GEO set. **(D)** Correlation heat map of 22 immune cells in the TARGET set. The *p*-value represents the significance of the difference; p < 0.05 is considered a significant difference.

For further immune infiltration analysis, we used CIBERSORT package and LM22 algorithm to calculate the infiltration abundance of 22 kinds of immune cells between high/low expression level of GNG12 groups in test and validation cohort, including different B cells, T cells, natural killer cells, plasma cells, and different myeloid subsets. The results were visualized as two violin maps (**Figures 6A, B**) and two correlation heat maps (**Figures 6C, D**) to reveal the differences in the expression of immune cells between groups.

### 3.5 Expression Analysis of GNG12-Related Genes

**Figure 7:**
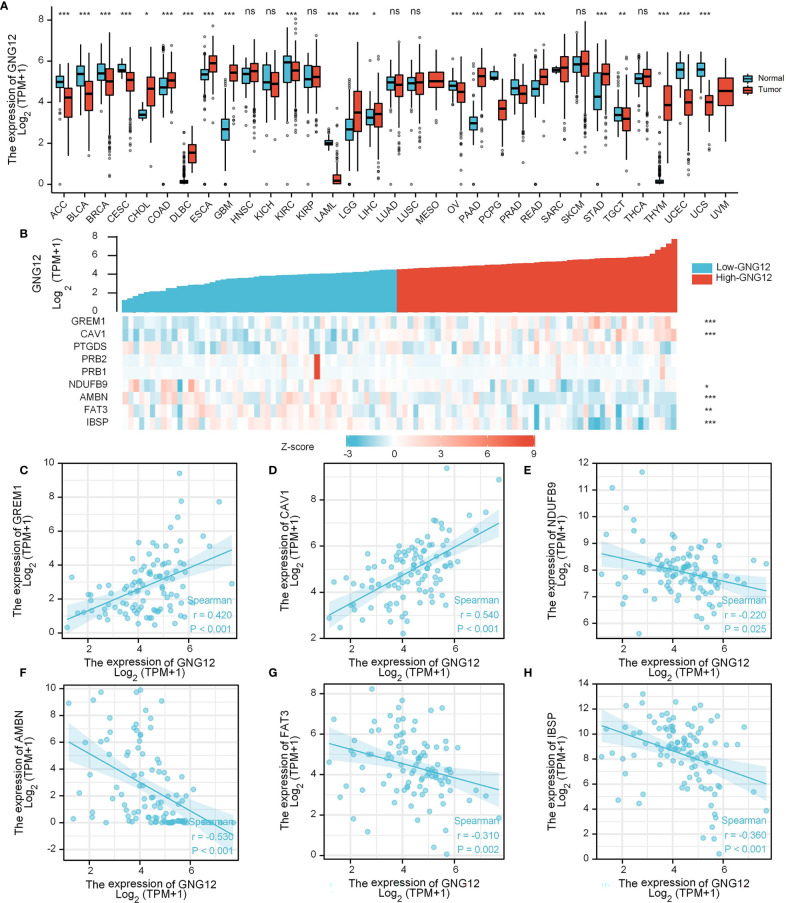
Expression analysis of GNG12-related genes. **(A)** Differential expression of GNG12 in pan-cancer. **(B)** A gene expression-related heat map of the nine common DEGs between GEO and TARGET sets. **(C–H)** Scatter plots of the expression correlation between GREM1, CAV1, NDUFB9, AMBN, FAT3, and IBSP and GNG12. Significance was set at p < 0.05 (ns, p < 0.05; *p < 0.05; **p < 0.01; ***p < 0.001).

Evaluation of differential GNG12 expression in pan-cancer revealed that GNG12 was downregulated in 12 tumor types (i.e., ACC, BLCA, BRCA, CESC, KIRC, LAML, OV, PCPG, PRAD, TGCT, UCEC, and UCS) and upregulated in 11 (CHOL, COAD, DLBC, ESCA, GBM, LGG, LIHC, PAAD, READ, STAD, and THYM; **Figure 7A**). When we used a gene-expression heat map to examine the nine common DEGs across GEO and TARGET cohorts, we found that GREM1 and CAV1 expression were positively correlated with GNG12 expression in the TARGET cohort. Additionally, NDUFB9, AMBN, FAT3, and IBSP expression were negatively correlated (**Figure 7B**). We then generated six scatterplots of correlations between the expression of these six genes and of GNG12 (**Figures 7C–H**).

### 3.6 Competitive Endogenous RNA Network

**Figure 8:**
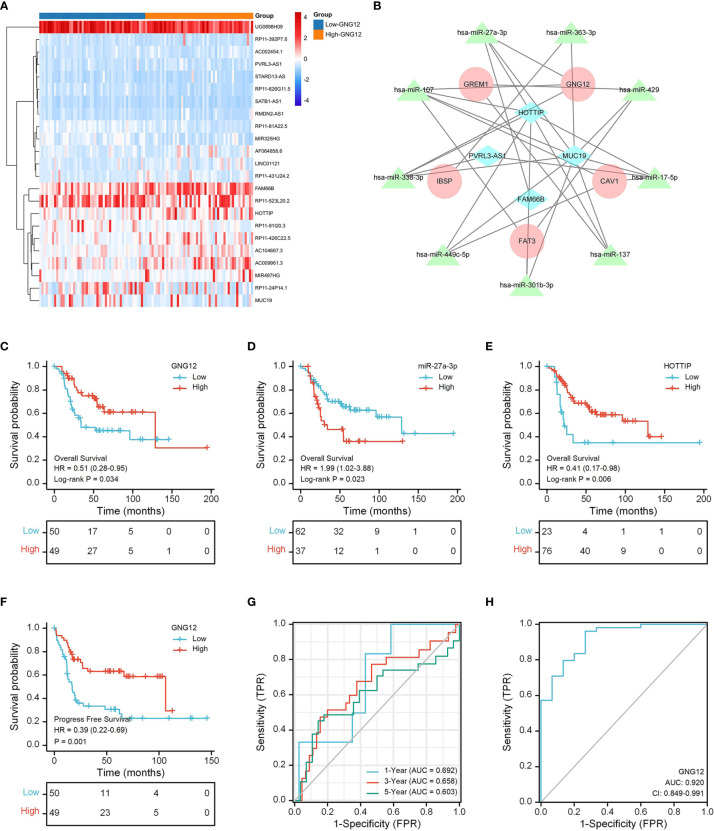
GNG12-related ceRNA network construction and survival analysis. **(A)** The TARGET data set shows the significant difference lncRNAs between the two groups in the form of a heat map. **(B)** A ceRNA network: blue diamond, lncRNAs; green triangle, miRNAs; red round, mRNAs. **(C)** The effect of low GNG12 expression on the prognosis of osteosarcoma overall survival (OS) is statistically significant. **(D)** The effect of high miR-27a-3p expression on the prognosis of osteosarcoma overall survival (OS) is statistically significant. **(E)** The effect of low HOTTIP expression on the prognosis of osteosarcoma overall survival (OS) is statistically significant. **(F)** The effect of low GNG12 expression on the prognosis of osteosarcoma progress-free survival (PFS) is statistically significant. **(G)** Time-dependent ROC curves, 1 year (AUC = 0.692), 3 years (AUC = 0.658), and 5 years (AUC = 0.603). **(H)** The area under the ROC curve (AUC) in the GEO set assesses the performance for distinguishing osteosarcoma from normal tissue of GNG12 (AUC = 0.92).

Using limma, we obtained 22 differentially expressed lncRNAs (DELs) from the TARGET database and visualized them on a heat map (**Figure 8A**). We then used the miRcode website to predict miRNAs that were highly conserved with DELs. Furthermore, we identified mRNAs targeted by the miRNAs through a series of databases and then constructed a ceRNA network with DEGs (**Figure 8B**).

### 3.7 Survival Analysis of GNG12 in TARGET Cohort

The results of our KM analysis indicated that OS and PFS differed significantly between low and high GNG-expression groups (**Figures 8C, F**). In addition, the ceRNA network revealed that low HOTTIP and GNG12 expression led to high risk. Elevated hsa-miR-27a-3p expression also resulted in high risk (**Figures 8D, E**). Through time-dependent ROC curves, we found that GNG12 was a reliable predictor of patient prognosis (**Figure 8G**).

We used an ROC curve to analyze whether GNG12 expression could distinguish between 15 normal samples and 103 tumor samples from the GEO dataset. We found an AUC of 0.920, indicating that GNG12 expression was an excellent biomarker for distinguishing osteosarcoma from normal tissue. (**Figure 8H**).

### 3.8 Validating Prognostic Value of GNG12 Expression in Osteosarcoma

**Figure 9:**
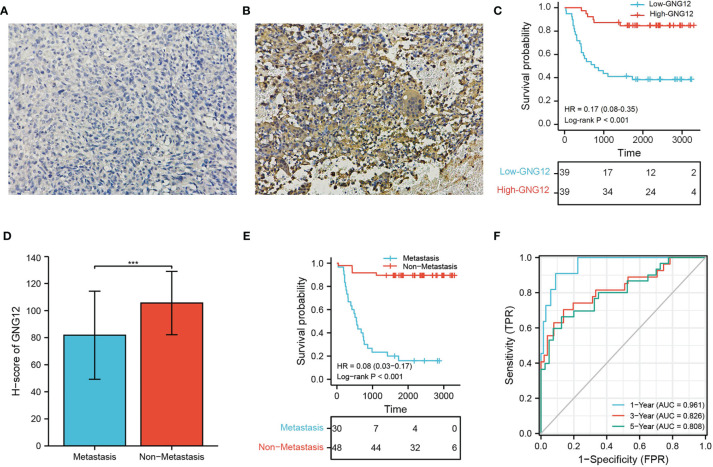
Validation the GNG12 expression and prognostic value of GNG12. **(A, B)** Low/high H-score of GNG12 ICH images. **(C)** The effect of low GNG12 expression on the prognosis of osteosarcoma overall survival (OS) is statistically significant in the validation cohort. **(D)** In the validation cohort, the H-score level of GNG12 between metastasis and non-metastasis groups. **(E)** The effect of metastasis on the prognosis of osteosarcoma overall survival (OS) is statistically significant in the validation cohort. **(F)** Time-dependent ROC curves: 1 year (AUC = 0.961), 3 years (AUC = 0.826), and 5 years (AUC = 0.808). ***p < 0.001.

We used 78 patients with osteosarcoma as a validation cohort to confirm the prognostic reliability of GNG12 (**Table 1** and **Supplementary Table S3**). GNG12 expression levels (high *vs*. low) of these patients were confirmed using immuno-histochemistry (IHC) (**Figures 9A, B**). The results of KM analysis showed that patients with low GNG12 expression tend to have poor survival prognosis (**Figure 9C**). Analysis of time-dependent ROC curves revealed that 1-/3-/5-year AUCs were 0.961, 0.826, and 0.808 (**Figure 9F**). In addition, GNG12 expression was lower in metastatic than in non-metastatic osteosarcoma (**Figure 9D**); patients with metastatic osteosarcoma have a poor survival prognosis (**Figure 9E**).

**Table 1:** Baseline clinical characteristics of validation cohort.

Characteristic	Low GNG12	High GNG12	*p*-value
**N**	39	39	
**Gender, n (%)**			0.425
Female	12 (15.6%)	16 (20.8%)	
Male	27 (35.1%)	22 (28.6%)	
**M/NM, n (%)**			0.036
Metastasis	20 (25.6%)	10 (12.8%)	
Non-Metastasis	19 (24.4%)	29 (37.2%)	
**Recurrence/non-recurrence, n (%)**			0.001
Non-recurrence	16 (20.5%)	31 (39.7%)	
Recurrence	23 (29.5%)	8 (10.3%)	
**Survival status, n (%)**			<0.001
Alive	15 (19.2%)	33 (42.3%)	
Dead	24 (30.8%)	6 (7.7%)	
**Age, median (IQR)**	25 (14.5, 32.5)	23 (17.5, 38.5)	0.545
**Survival time, median (IQR)**	767 (312.5, 2303)	2336 (1,639, 2,557)	<0.001

## 4 Discussion

The transducer and transmembrane-signal regulator GNG12 plays an important role in cancer progression through enhancing cell proliferation (15, 16). It is indispensable for guanosine triphosphatase (GTPase) activity, which functions in GTP-to-GDP catalysis (17). GTPase is associated with proliferation, invasion, and migration of tumors in multiple cancers, including osteosarcoma (18, 19). Despite this link, few studies have explored the role of GNG12 in osteosarcoma. Therefore, in this study, we hypothesized that GNG12 expression may influence osteosarcoma progression. Furthermore, GNG12 may have prognostic and therapeutic value for this cancer.

Analysis of data from GEO indicated that GNG12 expression was significantly downregulated in osteosarcoma tissues compared with normal samples. This reduced expression was positively correlated with poor prognosis in the TARGET cohort. Our functional analyses corroborated previous studies, showing that GNG12 was involved in ossification (20–22), protein targeting to ER (23, 24), extracellular-related terms (25, 26), focal adhesion (25, 27), PI3K-Akt signaling pathway (28, 29), GPCR ligand binding (30), and the matrisome. Through PPI networks, we identified the ER lumen as the location of hub gene clusters present in both GEO and TARGET cohorts. Regulating ER function plays a major role in the treatment of osteosarcoma (23, 31). Specifically, H_2_S-releasing doxorubicins caused misfolding of ER-related proteins to enhance ER-dependent apoptosis, which is effective against doxorubicin-resistant osteosarcoma *in vitro* (23). Additionally, lexibulin induces autophagy and apoptosis of osteosarcoma *in vitro* through triggering mutually enhanced reactive oxygen species and ER stress, suggesting that it could be an effective drug for osteosarcoma therapy (31). Taken together, our research and previous studies provided insight on GNG12’s role in cancer pathogenesis and demonstrated that the protein is a potential biomarker of osteosarcoma.

Integrated landscapes depicting the osteosarcoma microenvironment may help explain how the cancer responds to immunotherapy and help the development of new treatment strategies (32, 33). Our immune infiltration analysis of the GEO cohort showed that the high and low GNG12-expression groups differed significantly in macrophages (M0, M1, and M2), activated CD4+ memory T cells, mast cells, activated dendritic cells, and eosinophils. The same analysis on the TARGET cohort revealed that the three macrophage subtypes and mast cells were significantly different. Combining the two results indicated that macrophages and mast cells play a crucial role in the immune microenvironment of osteosarcoma. Moreover, osteosarcoma occurrence and development are probably related to inflammation and metabolic pathways. Thus, novel osteosarcoma therapy could potentially target macrophages and mast cells to improve their distribution in patients (34, 35). Findings from a mouse tail metastasis model indicated that M2 macrophages enhanced metastasis of osteosarcoma cells (K7M2 WT), thus identifying M2-polarized tumor-associated macrophages (TAMs) as a therapeutic target. The same study also found that all-trans retinoic acid (ATRA) inhibited M2 polarization of TAMs *via* downregulating MMP12 expression, thereby limiting osteosarcoma (36). Our study thus examined the correlation between GNG12 expression and MMP12. We found that MMP12 expression is negatively correlated with GNG12 expression, and high MMP12 expression can also predict poor osteosarcoma PFS (**Supplementary Figure S1A, B**). Therefore, GNG12 may inhibit M2 polarization of TAMs, which negatively regulates MMP12 expression and thus suppresses osteosarcoma metastasis.

Most studies have shown that ceRNA plays an important role in osteosarcoma occurrence and development (37, 38). We constructed a ceRNA network based on TARGET data and found that lncRNA HOTTIP (a low risk factor) can function as a ceRNA, decoying miR-27a-3p (a high risk factor) to promote GNG12-mediated metastasis. Targeting HOTTIP may therefore prove to be beneficial for osteosarcoma treatment (39). Liu et al. reported that miR-27a-3p were upregulated in osteosarcoma to promote the proliferation and invasion of osteosarcoma cells *via* inhibiting expression of TET1 (40). Although these results suggest that HOTTIP may regulate GNG12 through ceRNA and is involved in osteosarcoma progress, further experimental studies are needed to confirm these conclusions. Nevertheless, HOTTIP, miR-27a-3p, and GNG12 are all potential therapeutic targets for osteosarcoma.

This study has several limitations. First, the sample size of our healthy controls was much smaller than the sample size of patients with osteosarcoma. Future studies would benefit from balancing the number of participants in each group. Second, our outcomes were validated in a set of 78 patients with osteosarcoma, using an H-score to evaluate GNG12 expression in osteosarcoma tissue. Although the results confirmed that patients with low GNG12 expression had poor prognosis, this was a retrospective analysis. Thus, future research should employ a prospective methodology to avoid analysis bias. Finally, we did not verify our findings using *in vitro* or *in vivo* experiments, meaning that the exact mechanisms of GNG12 involvement in osteosarcoma remain unclear. This is an important topic for further investigation. Therefore, we highlight several areas in which further work is needed to deepen our understanding. First, as the response of GNG12, it would be interesting to examine the basic expression of these predicted DEGs and hub genes with Western blot, quantitative PCR (qPCR), IHC, immuno-fluorescence (IF) assays, and so on. Second, to clarify the function of GNG12, DEGs, and hub genes in osteosarcoma, a clean loss-of-function and gain-on-function study with tissue- and cell-type specificities remains warranted.

In conclusion, GNG12 mRNA and protein expression were downregulated in osteosarcoma, a change that was related to poor prognosis. We found that GNG12 may promote osteosarcoma through regulating ER function. HOTTIP/miR-27a-3p are candidates for regulating GNG12 expression to inhibit macrophage infiltration and suppress adaptive immunity. We provided important evidence supporting GNG12 as a biomarker for osteosarcoma prognosis and highlighting its potential as a target for immunotherapy.

## Data Availability Statement

The datasets analyzed for this study can be found in the TARGET repository (https://target-data.nci.nih.gov/Public/OS/mRNA-seq/) and Gene Expression Omnibus (GEO, https://ftp.ncbi.nlm.nih.gov/geo/series/GSE42nnn/GSE42352/matrix/). In addition, the validation cohort presented in this study can be found in online repositories. The names of the repository/repositories and accession number(s) can be found in the article/**Supplementary Material**.

## Ethics Statement

The studies involving human participants were reviewed and approved by Ethics Committee of The Second Affiliated Hospital of Nanchang University [Review (2020) No. (086)]. Written informed consent to participate in this study was provided by the participants’ legal guardian/next of kin.

## Author Contributions

JY, ZY, and XC contributed to the conception and design of this study. JY and ZY collected the data sets from the database. JY, TW, JJ, JG, JZ, and TL performed the bioinformatics and statistical analysis. JY, ZY, and AY wrote the first draft of the manuscript. All authors contributed to the article and approved the submitted version.

## Funding

The present study was supported by the National Natural Science Foundation of China (grant nos. 8166090137 and 8186090165), the National Natural Science Foundation of Jiangxi Province (No. 911084415069), and General Plan of Jiangxi Provincial Health Commission (No. 20195568).

## Conflict of Interest

The authors declare that the research was conducted in the absence of any commercial or financial relationships that could be construed as a potential conflict of interest.

## Publisher’s Note

All claims expressed in this article are solely those of the authors and do not necessarily represent those of their affiliated organizations, or those of the publisher, the editors and the reviewers. Any product that may be evaluated in this article, or claim that may be made by its manufacturer, is not guaranteed or endorsed by the publisher.
