# Supplementary figures and images for: Low GNG12 Expression Predicts Adverse Outcomes: A Potential Therapeutic Target for Osteosarcoma
Source: Front Immunol. 2021 Oct 6;12:758845. doi: 10.3389/fimmu.2021.758845 (PMC8527884; doi:10.3389/fimmu.2021.758845)

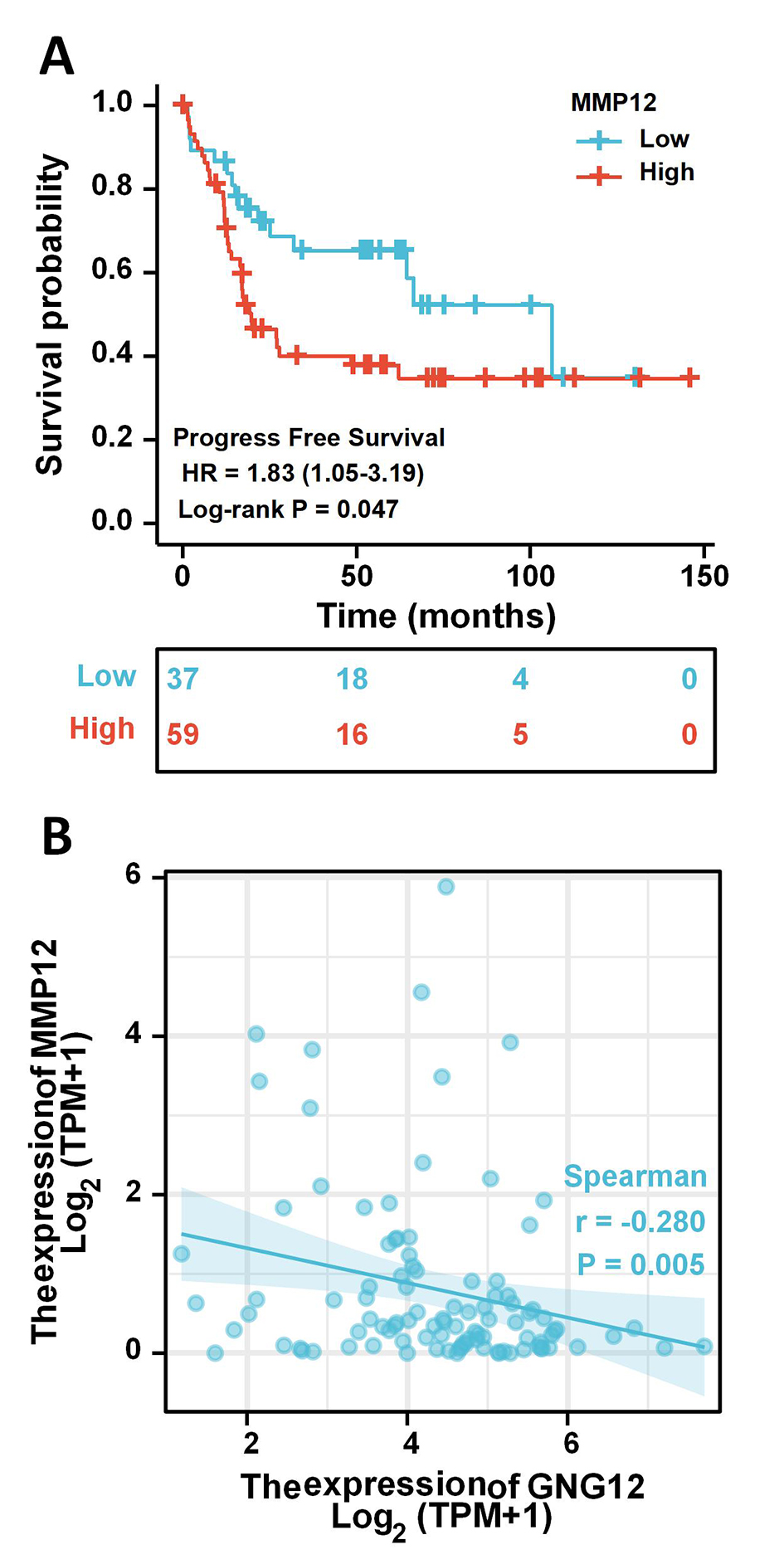

Supplement: Supplementary file 1 [file Image_1.jpeg]
