# Supplementary material for: Low GNG12 Expression Predicts Adverse Outcomes: A Potential Therapeutic Target for Osteosarcoma
Source: Front Immunol. 2021 Oct 6;12:758845. doi: 10.3389/fimmu.2021.758845 (PMC8527884; doi:10.3389/fimmu.2021.758845)
Supplement: Supplementary file 2 [file Table_1.docx]

| **ID** | **logFC** | **logCPM** | **PValue** | **FDR** |
| --- | --- | --- | --- | --- |
| **LOC644063** | -1.63443135 | 5.69619073 | 1.33E-27 | 3.31E-23 |
| **LOC650230** | -1.031478374 | 5.117192502 | 9.16E-22 | 5.72E-18 |
| **LOC653994** | -1.30038566 | 5.101445163 | 2.35E-21 | 9.79E-18 |
| **LOC653942** | -1.340077909 | 6.732466127 | 2.37E-19 | 7.39E-16 |
| **SBDSP** | -1.020583563 | 5.206530826 | 6.16E-19 | 1.71E-15 |
| **SLC5A8** | 1.291145304 | 4.962813718 | 1.15E-18 | 2.87E-15 |
| **LYPLA1** | -1.025930789 | 5.022377518 | 6.69E-18 | 1.52E-14 |
| **PRB2** | 3.36684066 | 6.077266597 | 2.69E-17 | 4.19E-14 |
| **LOC440704** | 1.68163558 | 6.370644314 | 3.44E-17 | 5.06E-14 |
| **LOC641704** | 1.477621018 | 5.944378912 | 1.89E-16 | 2.48E-13 |
| **ET** | 1.180524971 | 6.087503555 | 6.19E-16 | 6.44E-13 |
| **MRPL51** | 1.159470087 | 7.346863305 | 1.43E-15 | 1.34E-12 |
| **LOC642989** | 2.101855909 | 6.966305374 | 2.48E-15 | 2.00E-12 |
| **GDI2** | 1.363214472 | 7.452389524 | 3.21E-15 | 2.49E-12 |
| **LOC643949** | 1.580467375 | 6.254086861 | 3.50E-15 | 2.51E-12 |
| **LOC648852** | 1.012323645 | 4.688064072 | 3.73E-15 | 2.59E-12 |
| **RPL27A** | 1.543358231 | 5.436711094 | 5.54E-15 | 3.46E-12 |
| **USP49** | 1.935638919 | 7.732810864 | 6.46E-15 | 3.85E-12 |
| **LOC644037** | -1.220368707 | 8.410101577 | 9.02E-15 | 5.12E-12 |
| **FAM63A** | 1.297918399 | 6.145401996 | 1.05E-14 | 5.57E-12 |
| **RAXL1** | 1.091753611 | 5.088334922 | 1.10E-14 | 5.63E-12 |
| **LOC440341** | 1.068049563 | 5.397442762 | 1.10E-14 | 5.63E-12 |
| **FAM98A** | 1.481588967 | 5.883109381 | 1.15E-14 | 5.74E-12 |
| **DKFZp762I137** | 1.289753684 | 6.29684935 | 1.29E-14 | 6.20E-12 |
| **LOC653489** | 1.533160875 | 6.16294392 | 1.36E-14 | 6.41E-12 |
| **PTPLAD2** | 1.651742011 | 6.358759634 | 1.85E-14 | 8.43E-12 |
| **LOC643287** | 1.422111708 | 5.333607974 | 1.90E-14 | 8.49E-12 |
| **LOC388344** | 1.093954999 | 4.615446331 | 2.66E-14 | 1.13E-11 |
| **LRRFIP1** | 1.189024085 | 6.081554367 | 2.75E-14 | 1.13E-11 |
| **WSB2** | 1.160621045 | 5.033140471 | 2.77E-14 | 1.13E-11 |
| **LOC647389** | 1.079737529 | 5.326538796 | 2.85E-14 | 1.15E-11 |
| **PIP5K2B** | 1.259716745 | 6.139802379 | 2.90E-14 | 1.15E-11 |
| **HSD17B7** | 1.610315989 | 7.932082679 | 3.33E-14 | 1.30E-11 |
| **TRIP12** | 1.080145654 | 5.387046485 | 3.45E-14 | 1.33E-11 |
| **LOC644250** | 1.709756264 | 6.735092228 | 4.74E-14 | 1.73E-11 |
| **TDRD1** | 1.142610707 | 5.435113985 | 4.77E-14 | 1.73E-11 |
| **ZNF223** | 1.530055506 | 6.472341089 | 4.90E-14 | 1.73E-11 |
| **RPL14** | 1.518169141 | 6.440836406 | 4.90E-14 | 1.73E-11 |
| **PRB1** | 2.604928967 | 5.390029729 | 6.00E-14 | 2.08E-11 |
| **ACTG1** | -1.436292536 | 8.762908698 | 6.44E-14 | 2.19E-11 |
| **FLJ11712** | 1.011491057 | 4.575993696 | 6.50E-14 | 2.19E-11 |
| **LOC653596** | 1.242856575 | 5.615068889 | 7.28E-14 | 2.43E-11 |
| **FAT** | 1.552391013 | 7.494390285 | 8.54E-14 | 2.67E-11 |
| **HNRPA3** | -1.185020691 | 5.439454316 | 8.88E-14 | 2.73E-11 |
| **WASPIP** | 1.247994275 | 5.045923426 | 8.96E-14 | 2.73E-11 |
| **LOC645138** | 1.790014733 | 8.746646383 | 9.18E-14 | 2.76E-11 |
| **ALPP** | 1.62736799 | 8.11439108 | 1.27E-13 | 3.60E-11 |
| **C1orf63** | 1.028803991 | 5.34469157 | 1.31E-13 | 3.64E-11 |
| **TAF15** | -1.081319277 | 7.411187471 | 1.53E-13 | 4.20E-11 |
| **LOC653086** | 1.292149634 | 5.834349393 | 3.06E-13 | 7.57E-11 |
| **UQCRH** | 1.519849889 | 5.527731037 | 3.60E-13 | 8.59E-11 |
| **LOC649518** | 1.753021586 | 6.276748214 | 3.90E-13 | 9.20E-11 |
| **LMOD3** | 1.399749634 | 6.803824916 | 4.66E-13 | 1.02E-10 |
| **C20orf108** | 1.08927592 | 5.351443837 | 4.69E-13 | 1.02E-10 |
| **TRIB3** | -1.608418119 | 5.970511725 | 5.19E-13 | 1.12E-10 |
| **PTMA** | 1.992844887 | 6.639402294 | 7.86E-13 | 1.61E-10 |
| **MCART1** | 1.688292583 | 9.27178439 | 9.29E-13 | 1.87E-10 |
| **IL8** | -2.413012669 | 6.197370035 | 1.01E-12 | 2.02E-10 |
| **RPS28** | 1.662521053 | 9.560455723 | 1.11E-12 | 2.19E-10 |
| **LOC388621** | 1.932195127 | 7.633092618 | 1.13E-12 | 2.21E-10 |
| **C9orf130** | 1.026064735 | 5.368371182 | 1.39E-12 | 2.64E-10 |
| **WDSOF1** | 1.101690605 | 4.555610915 | 1.61E-12 | 2.97E-10 |
| **DKFZp727G131** | 1.633887831 | 8.478854549 | 1.70E-12 | 3.12E-10 |
| **SLC16A3** | 1.279398903 | 6.245207287 | 2.21E-12 | 3.97E-10 |
| **GABRB1** | 2.295014947 | 5.187268499 | 2.31E-12 | 4.09E-10 |
| **KPNA2** | 1.224820261 | 5.078151996 | 2.57E-12 | 4.46E-10 |
| **LOC641848** | 1.414048971 | 5.308007444 | 2.98E-12 | 5.03E-10 |
| **ASNS** | -1.480496647 | 7.273608305 | 3.77E-12 | 6.11E-10 |
| **LOC387753** | 1.642445309 | 8.072895609 | 3.98E-12 | 6.37E-10 |
| **RPN2** | 1.19702253 | 7.296354897 | 4.29E-12 | 6.70E-10 |
| **CEP27** | 1.116644735 | 5.819966674 | 4.31E-12 | 6.70E-10 |
| **ATP1B3** | 1.122852799 | 6.074105779 | 5.13E-12 | 7.76E-10 |
| **FAT3** | 1.372441593 | 5.62549537 | 5.30E-12 | 7.87E-10 |
| **ACTR3** | 1.407756483 | 5.684714999 | 5.37E-12 | 7.94E-10 |
| **LOC284988** | 1.159230711 | 5.036687861 | 5.85E-12 | 8.55E-10 |
| **LOC644914** | 1.529663212 | 7.184409752 | 6.15E-12 | 8.84E-10 |
| **ANKRD30B** | 1.270674982 | 6.678965178 | 7.36E-12 | 1.04E-09 |
| **PRB3** | 2.12387769 | 4.960255776 | 7.74E-12 | 1.08E-09 |
| **LOC388532** | 1.443758876 | 7.809234319 | 7.88E-12 | 1.08E-09 |
| **RPS3A** | 1.273075791 | 9.264382425 | 7.98E-12 | 1.09E-09 |
| **ZNF14** | 1.715846601 | 8.66204716 | 8.45E-12 | 1.12E-09 |
| **AGPAT5** | 1.210594972 | 5.666585466 | 8.46E-12 | 1.12E-09 |
| **LOC220433** | 1.748009252 | 7.065313633 | 9.86E-12 | 1.28E-09 |
| **VGF** | -1.624421828 | 4.604773362 | 1.12E-11 | 1.42E-09 |
| **PHTF2** | 1.043913385 | 5.178096376 | 1.13E-11 | 1.43E-09 |
| **OSBPL8** | 1.026266995 | 4.820186046 | 1.16E-11 | 1.45E-09 |
| **IL1B** | -1.740973908 | 5.106148019 | 1.20E-11 | 1.49E-09 |
| **LOC347376** | 1.1271838 | 4.967242945 | 1.23E-11 | 1.52E-09 |
| **DKFZp564K142** | 1.467114977 | 7.67173658 | 1.50E-11 | 1.82E-09 |
| **LOC644972** | 1.522688802 | 8.901490497 | 1.82E-11 | 2.13E-09 |
| **LOC642749** | 1.044434517 | 6.476129392 | 2.72E-11 | 3.00E-09 |
| **CAV1** | -1.051666701 | 5.049897478 | 2.86E-11 | 3.14E-09 |
| **IBTK** | 1.016350923 | 5.157754881 | 3.23E-11 | 3.47E-09 |
| **YRDC** | 1.052264957 | 6.501878018 | 3.28E-11 | 3.50E-09 |
| **KRT18** | -1.773749138 | 5.313857614 | 4.76E-11 | 4.87E-09 |
| **LDHB** | 1.292995202 | 7.07612585 | 4.79E-11 | 4.88E-09 |
| **SAR1A** | 1.015974394 | 5.327344463 | 4.81E-11 | 4.88E-09 |
| **DKFZp434I1020** | 1.437232328 | 8.37879907 | 4.82E-11 | 4.88E-09 |
| **MTDH** | 1.115407045 | 6.199478823 | 6.57E-11 | 6.36E-09 |
| **ARPC5** | 1.239411879 | 7.626048244 | 6.73E-11 | 6.49E-09 |
| **IL6** | -1.561764709 | 4.748568281 | 6.95E-11 | 6.61E-09 |
| **LOC643007** | 1.211407442 | 5.173227955 | 7.26E-11 | 6.84E-09 |
| **LOC647100** | 1.626293278 | 6.328767813 | 7.62E-11 | 7.05E-09 |
| **FRG1** | 1.095650769 | 5.573060307 | 7.65E-11 | 7.05E-09 |
| **FH** | 1.086802418 | 5.82852764 | 9.39E-11 | 8.38E-09 |
| **LOC648294** | 1.634553116 | 6.494809038 | 9.50E-11 | 8.45E-09 |
| **LOC646433** | 1.124868327 | 9.952862914 | 1.00E-10 | 8.85E-09 |
| **LOC651751** | -2.03930931 | 5.182236856 | 1.07E-10 | 9.41E-09 |
| **FTHL2** | 1.559210105 | 7.092744179 | 1.35E-10 | 1.14E-08 |
| **NUCB2** | 1.158339904 | 5.934243157 | 1.55E-10 | 1.30E-08 |
| **NDUFB9** | 1.496203421 | 6.969736556 | 1.62E-10 | 1.35E-08 |
| **EIF3S10** | 1.112517885 | 6.159273667 | 2.01E-10 | 1.64E-08 |
| **LOC440732** | 1.592999265 | 7.412466113 | 2.04E-10 | 1.66E-08 |
| **FTHL12** | 1.63480265 | 6.838153088 | 2.23E-10 | 1.79E-08 |
| **C21orf55** | 1.25937752 | 9.950148017 | 2.33E-10 | 1.84E-08 |
| **LOC401206** | 1.493691453 | 8.935771287 | 2.48E-10 | 1.94E-08 |
| **LOC440567** | 1.253484904 | 9.13640481 | 2.86E-10 | 2.22E-08 |
| **RPL23** | 1.803279746 | 7.794635962 | 4.23E-10 | 3.09E-08 |
| **ITM2A** | 1.292200781 | 4.65508813 | 4.60E-10 | 3.33E-08 |
| **SUMO2** | 1.699162738 | 7.081013985 | 4.70E-10 | 3.38E-08 |
| **FTHL11** | 1.219549997 | 5.847484637 | 4.83E-10 | 3.45E-08 |
| **CCT3** | 1.001584546 | 6.160336636 | 5.06E-10 | 3.56E-08 |
| **IER3** | -1.644011882 | 7.811103205 | 5.45E-10 | 3.76E-08 |
| **LOC644969** | 1.55025197 | 7.277873147 | 5.80E-10 | 3.98E-08 |
| **PTGES3** | 1.496970177 | 6.280565433 | 6.67E-10 | 4.49E-08 |
| **ZNF486** | 1.387126393 | 9.370513704 | 7.37E-10 | 4.86E-08 |
| **MCM8** | 1.277264424 | 7.925127558 | 7.59E-10 | 4.99E-08 |
| **LGALS7** | -1.700722648 | 4.870518823 | 7.98E-10 | 5.22E-08 |
| **RPLP1** | 1.606689715 | 6.627227516 | 8.32E-10 | 5.38E-08 |
| **RPL10A** | 1.215869162 | 6.987202921 | 1.08E-09 | 6.79E-08 |
| **C3orf28** | 1.154621359 | 4.830306352 | 1.12E-09 | 7.00E-08 |
| **PSAT1** | -1.03400635 | 5.528554825 | 1.14E-09 | 7.11E-08 |
| **RPL7** | 1.406684882 | 6.629687278 | 1.20E-09 | 7.41E-08 |
| **CDH2** | 1.388535047 | 7.370047866 | 1.52E-09 | 9.21E-08 |
| **LOC649682** | 1.568532026 | 7.694256812 | 1.66E-09 | 9.77E-08 |
| **LOC389787** | 1.66598662 | 7.552099013 | 1.75E-09 | 1.02E-07 |
| **LOC653780** | 1.177397165 | 9.934272834 | 1.80E-09 | 1.04E-07 |
| **GMFG** | 1.011828524 | 5.588613991 | 1.99E-09 | 1.13E-07 |
| **GPT2** | -1.004578769 | 5.86656305 | 2.13E-09 | 1.20E-07 |
| **ALDH1A3** | -1.719390017 | 5.44216818 | 2.15E-09 | 1.21E-07 |
| **IL18** | 1.088221614 | 9.701676762 | 2.26E-09 | 1.27E-07 |
| **LOC653658** | 1.642826042 | 8.45769363 | 2.37E-09 | 1.32E-07 |
| **SULF2** | 1.011041888 | 4.54648349 | 2.55E-09 | 1.39E-07 |
| **A2M** | 1.861213998 | 7.689590691 | 3.14E-09 | 1.67E-07 |
| **GHR** | 1.059185609 | 5.333805265 | 3.32E-09 | 1.74E-07 |
| **AMY1C** | 1.298014736 | 9.168639549 | 3.33E-09 | 1.74E-07 |
| **HLA-DQB2** | 1.623668397 | 4.776296663 | 4.93E-09 | 2.50E-07 |
| **CST1** | 1.567897829 | 4.638543979 | 5.12E-09 | 2.58E-07 |
| **LOC643516** | 1.060710796 | 8.103480216 | 6.05E-09 | 2.99E-07 |
| **LOC653499** | -1.429901386 | 4.530742405 | 6.95E-09 | 3.34E-07 |
| **ITM2B** | 1.209405523 | 8.023607213 | 7.63E-09 | 3.60E-07 |
| **IFP38** | 1.087812433 | 5.548120454 | 8.26E-09 | 3.83E-07 |
| **CD68** | 1.628884313 | 8.36029642 | 8.90E-09 | 4.08E-07 |
| **LOC284230** | 1.720752626 | 7.266568607 | 8.99E-09 | 4.10E-07 |
| **CYR61** | -1.160481308 | 6.476092066 | 9.26E-09 | 4.21E-07 |
| **RBBP7** | 1.006941144 | 5.969864557 | 9.72E-09 | 4.40E-07 |
| **PPT1** | 1.062725166 | 7.689197553 | 9.84E-09 | 4.45E-07 |
| **SEPT11** | 1.336810979 | 6.412809956 | 1.02E-08 | 4.57E-07 |
| **LOC653328** | 1.280941271 | 9.113840567 | 1.38E-08 | 5.82E-07 |
| **HNRPA2B1** | 1.050873656 | 8.13817439 | 1.47E-08 | 6.09E-07 |
| **FTHL8** | 1.473282082 | 6.589773437 | 1.79E-08 | 7.19E-07 |
| **LOC646483** | 1.060351304 | 8.581291294 | 2.26E-08 | 8.89E-07 |
| **KRTHB1** | -1.348277865 | 4.56095365 | 2.92E-08 | 1.11E-06 |
| **CPE** | 1.618908183 | 7.646984768 | 3.05E-08 | 1.15E-06 |
| **EEF1A2** | -1.952832498 | 6.616237231 | 3.24E-08 | 1.20E-06 |
| **SLC7A5** | -1.289581701 | 7.027082077 | 3.75E-08 | 1.37E-06 |
| **H2BFS** | -1.058503508 | 6.264087054 | 3.83E-08 | 1.39E-06 |
| **LOC653974** | 1.363954702 | 7.257671268 | 4.42E-08 | 1.57E-06 |
| **GJA1** | 1.457724834 | 8.306887305 | 4.44E-08 | 1.57E-06 |
| **CDH11** | 1.224073183 | 7.49894827 | 4.77E-08 | 1.68E-06 |
| **NTS** | 1.290527796 | 4.313494446 | 5.39E-08 | 1.87E-06 |
| **LOC647450** | -1.836491951 | 5.765425191 | 6.55E-08 | 2.18E-06 |
| **SSPN** | 1.037058035 | 5.168626292 | 6.58E-08 | 2.18E-06 |
| **SLPI** | -1.840666885 | 5.798832379 | 6.74E-08 | 2.22E-06 |
| **TUBB3** | -1.101487679 | 5.955281716 | 7.33E-08 | 2.40E-06 |
| **NPPB** | -1.126587172 | 4.206296367 | 7.85E-08 | 2.55E-06 |
| **LOC653247** | 1.235223309 | 4.355289551 | 7.97E-08 | 2.58E-06 |
| **THRA** | 1.154855102 | 5.429797856 | 8.76E-08 | 2.80E-06 |
| **TIMP2** | 1.066481695 | 6.763373742 | 1.28E-07 | 3.84E-06 |
| **LOC647727** | 1.266412513 | 6.715542907 | 1.54E-07 | 4.45E-06 |
| **LOC653341** | 1.043221454 | 9.011888855 | 2.53E-07 | 6.80E-06 |
| **PHGDH** | -1.049092694 | 8.677770012 | 4.08E-07 | 1.03E-05 |
| **MGC35169** | -1.260666911 | 4.720041319 | 4.37E-07 | 1.09E-05 |
| **LOC652493** | -1.816303543 | 6.078307129 | 4.80E-07 | 1.18E-05 |
| **CSPG2** | 1.380005158 | 7.260888514 | 4.86E-07 | 1.20E-05 |
| **POSTN** | 1.296975667 | 5.639218672 | 4.91E-07 | 1.21E-05 |
| **CRHBP** | 1.116028042 | 4.248416458 | 5.22E-07 | 1.27E-05 |
| **PHLDA1** | -1.001662196 | 6.1292605 | 5.76E-07 | 1.38E-05 |
| **RELN** | -1.131210968 | 4.494277197 | 1.01E-06 | 2.20E-05 |
| **CST6** | 1.19433463 | 4.525336527 | 1.13E-06 | 2.41E-05 |
| **LOC642115** | 1.09119363 | 7.30816313 | 1.14E-06 | 2.43E-05 |
| **NPY** | 1.383930349 | 4.806607814 | 1.31E-06 | 2.72E-05 |
| **AXL** | -1.113826738 | 6.901938188 | 1.49E-06 | 3.05E-05 |
| **MLPH** | -1.039378489 | 5.14785754 | 1.79E-06 | 3.57E-05 |
| **HSPA1A** | 1.389470554 | 7.111956995 | 3.62E-06 | 6.46E-05 |
| **LOC402251** | 1.162352103 | 9.017249948 | 4.32E-06 | 7.53E-05 |
| **FRZB** | 1.072418217 | 5.054007659 | 6.06E-06 | 0.000100467 |
| **IBSP** | 1.537734478 | 7.795149368 | 7.46E-06 | 0.000119803 |
| **LOC654194** | 1.095629619 | 8.788396252 | 8.19E-06 | 0.000129297 |
| **GREM1** | -1.027004621 | 5.251577673 | 9.16E-06 | 0.000141901 |
| **NQO1** | -1.06542187 | 6.169694141 | 1.04E-05 | 0.00015878 |
| **AMBN** | 1.217322965 | 4.947563659 | 1.19E-05 | 0.000179415 |
| **EGFL6** | 1.035228349 | 4.747909809 | 1.30E-05 | 0.000192858 |
| **MAMDC2** | 1.027967388 | 6.177588188 | 1.67E-05 | 0.000236042 |
| **LOC652694** | -1.022443349 | 4.596968791 | 3.15E-05 | 0.000402481 |
| **PTHR1** | 1.037679499 | 8.065951514 | 0.000215079 | 0.002023929 |
| **COL3A1** | 1.097509483 | 9.356558515 | 0.000230844 | 0.002148202 |
| **PTGDS** | -1.068785051 | 5.279665903 | 0.000352097 | 0.003059755 |
| **PAGE2B** | 1.277154708 | 6.404161318 | 0.000940278 | 0.00699372 |
| **MYH7** | 1.057154349 | 5.216660666 | 0.001225781 | 0.008739986 |
