# Supplementary material for: Low GNG12 Expression Predicts Adverse Outcomes: A Potential Therapeutic Target for Osteosarcoma
Source: Front Immunol. 2021 Oct 6;12:758845. doi: 10.3389/fimmu.2021.758845 (PMC8527884; doi:10.3389/fimmu.2021.758845)
Supplement: Supplementary file 3 [file Table_2.docx]

| **ID** | **logFC** | **logCPM** | **PValue** | **FDR** |
| --- | --- | --- | --- | --- |
| **GNG12** | -1.672165471 | 5.151677876 | 1.40E-22 | 1.54E-18 |
| **DDIT4L** | -3.771531832 | 4.590815528 | 1.50E-17 | 8.29E-14 |
| **CYFIP2** | 2.389766 | 5.356989979 | 3.25E-17 | 1.20E-13 |
| **HCRT** | 6.2969778 | 3.763079818 | 2.97E-16 | 8.21E-13 |
| **AMELX** | 7.899135421 | 4.648284628 | 2.01E-15 | 4.44E-12 |
| **SCGB3A1** | -5.22876501 | 6.147139541 | 4.12E-15 | 7.59E-12 |
| **DKK1** | 2.906758327 | 5.158502951 | 1.20E-14 | 1.89E-11 |
| **FAP** | -1.942589592 | 5.685618075 | 1.28E-13 | 1.76E-10 |
| **SLC7A8** | -1.589519197 | 5.807403213 | 2.71E-13 | 3.33E-10 |
| **PRB2** | 7.128827085 | 5.73064952 | 3.04E-13 | 3.35E-10 |
| **CORT** | 2.112755308 | 3.800337314 | 3.77E-13 | 3.79E-10 |
| **ITK** | 2.144043163 | 3.516920935 | 4.67E-13 | 4.29E-10 |
| **APCDD1** | 2.091326805 | 6.864350802 | 1.52E-12 | 1.29E-09 |
| **TAC4** | 2.376258083 | 3.887595103 | 2.27E-12 | 1.79E-09 |
| **POP4** | 2.10222236 | 7.521562218 | 4.32E-12 | 3.06E-09 |
| **RASGRP2** | 2.036962381 | 4.217735649 | 4.43E-12 | 3.06E-09 |
| **C1QTNF3** | -3.035840549 | 6.007810027 | 6.17E-12 | 4.01E-09 |
| **GREM1** | -2.50573187 | 4.66736017 | 1.64E-11 | 1.00E-08 |
| **OPN3** | 1.841258952 | 6.259696093 | 1.74E-11 | 1.01E-08 |
| **FAM166B** | 2.911032456 | 3.595716818 | 2.72E-11 | 1.50E-08 |
| **SPON2** | -1.941201939 | 5.719003847 | 3.59E-11 | 1.89E-08 |
| **MEPE** | 3.363499674 | 5.384184316 | 5.58E-11 | 2.77E-08 |
| **PTPRZ1** | 2.37144158 | 5.152579053 | 5.77E-11 | 2.77E-08 |
| **COL3A1** | -1.881877989 | 11.09868816 | 6.70E-11 | 3.08E-08 |
| **NRXN1** | 2.447545708 | 3.929317204 | 7.88E-11 | 3.48E-08 |
| **ROBO2** | 1.550283469 | 4.620613851 | 1.24E-10 | 5.28E-08 |
| **FBLN1** | -1.691961205 | 7.071281622 | 1.76E-10 | 7.22E-08 |
| **GPC3** | -2.480383857 | 3.609823114 | 1.88E-10 | 7.39E-08 |
| **CHMP4C** | 2.047905218 | 3.754489629 | 2.84E-10 | 1.08E-07 |
| **COL14A1** | -2.38604122 | 5.679967502 | 3.65E-10 | 1.34E-07 |
| **UQCRFS1** | 1.88690638 | 6.248264502 | 3.86E-10 | 1.38E-07 |
| **ITPR1** | -1.454831145 | 3.727282083 | 4.31E-10 | 1.49E-07 |
| **EGFR** | -1.465733223 | 3.516813791 | 5.63E-10 | 1.84E-07 |
| **SOST** | 3.34108367 | 5.811727308 | 5.65E-10 | 1.84E-07 |
| **GNGT2** | 1.637427645 | 3.302304051 | 6.02E-10 | 1.90E-07 |
| **SMPD3** | 1.863963624 | 5.126882668 | 8.13E-10 | 2.49E-07 |
| **PANX3** | 2.288364994 | 7.625136123 | 8.47E-10 | 2.53E-07 |
| **DLX3** | 1.60456073 | 5.53923967 | 1.05E-09 | 3.00E-07 |
| **FAM180A** | -2.311047423 | 3.563169741 | 1.06E-09 | 3.00E-07 |
| **PCSK5** | 2.518448722 | 6.25601163 | 2.86E-09 | 7.90E-07 |
| **GADD45A** | -1.236472521 | 5.309447546 | 3.00E-09 | 8.07E-07 |
| **TAC3** | 3.485227155 | 6.618875298 | 3.23E-09 | 8.49E-07 |
| **HOMER2** | 1.407547782 | 5.543548907 | 3.46E-09 | 8.87E-07 |
| **MRGPRF** | -2.327643016 | 3.334127862 | 3.55E-09 | 8.87E-07 |
| **PLA2G16** | 1.59844607 | 5.440299701 | 3.62E-09 | 8.87E-07 |
| **SP6** | 2.080335441 | 3.652158327 | 4.06E-09 | 9.52E-07 |
| **CYP26B1** | -1.421087261 | 3.379417127 | 4.19E-09 | 9.64E-07 |
| **MTSS1** | 1.452485318 | 5.584203685 | 4.30E-09 | 9.64E-07 |
| **C1S** | -1.682625084 | 6.322305997 | 4.37E-09 | 9.64E-07 |
| **DLK1** | -5.595328808 | 3.516926556 | 5.01E-09 | 1.08E-06 |
| **CALD1** | -1.114947749 | 7.299876969 | 5.53E-09 | 1.16E-06 |
| **PRB1** | 5.803862506 | 3.953095683 | 5.93E-09 | 1.19E-06 |
| **PDGFRL** | -1.583698308 | 4.653790179 | 5.95E-09 | 1.19E-06 |
| **CRISPLD2** | -1.638221641 | 4.651803127 | 6.19E-09 | 1.22E-06 |
| **PODN** | -1.631469944 | 3.71483523 | 6.68E-09 | 1.27E-06 |
| **FOLR1** | 1.77568816 | 4.688175475 | 7.20E-09 | 1.35E-06 |
| **HSPA12B** | -1.40979496 | 3.487678492 | 7.35E-09 | 1.35E-06 |
| **SYT12** | 1.61335913 | 3.7284769 | 8.00E-09 | 1.41E-06 |
| **SMAD6** | 1.111507709 | 5.300374108 | 8.07E-09 | 1.41E-06 |
| **HTRA1** | -1.336656522 | 8.242704244 | 8.19E-09 | 1.41E-06 |
| **COL15A1** | -1.166851686 | 5.081751436 | 8.19E-09 | 1.41E-06 |
| **HIST3H2A** | 1.870799875 | 4.339052046 | 9.29E-09 | 1.58E-06 |
| **LRIG3** | -1.5411288 | 4.681750738 | 9.69E-09 | 1.62E-06 |
| **ASPSCR1** | 1.098075007 | 6.197455005 | 9.96E-09 | 1.63E-06 |
| **C1RL** | -1.072485195 | 3.914972089 | 1.01E-08 | 1.63E-06 |
| **RPS6KA6** | 1.501017052 | 3.357696675 | 1.08E-08 | 1.73E-06 |
| **CRISPLD1** | -1.591222375 | 5.609933693 | 1.39E-08 | 2.19E-06 |
| **SLC16A8** | 1.812711203 | 3.310297908 | 1.46E-08 | 2.26E-06 |
| **TGFB3** | -1.170629597 | 6.6531659 | 1.51E-08 | 2.29E-06 |
| **TMCC2** | 1.317144457 | 3.481417095 | 1.62E-08 | 2.42E-06 |
| **ARID3B** | 1.922311061 | 3.404909589 | 1.88E-08 | 2.76E-06 |
| **AMPH** | -1.536344668 | 3.412851548 | 1.92E-08 | 2.79E-06 |
| **ARHGAP44** | 1.293645433 | 3.584428589 | 2.09E-08 | 2.99E-06 |
| **MCAM** | 1.048621507 | 7.033320157 | 2.34E-08 | 3.31E-06 |
| **HRH1** | -1.236117195 | 3.266636091 | 2.38E-08 | 3.31E-06 |
| **SEC11C** | 1.136545135 | 5.715648279 | 2.40E-08 | 3.31E-06 |
| **BHLHE41** | -1.398716998 | 4.286577889 | 2.60E-08 | 3.54E-06 |
| **RARRES1** | 1.61247628 | 4.016852905 | 3.22E-08 | 4.28E-06 |
| **AEBP1** | -1.520787606 | 8.554929735 | 3.33E-08 | 4.38E-06 |
| **SMOC2** | -1.879854305 | 5.204730543 | 3.91E-08 | 5.07E-06 |
| **TNFRSF19** | -1.11059878 | 4.581049177 | 4.19E-08 | 5.37E-06 |
| **JCHAIN** | -3.196168147 | 5.298646064 | 6.67E-08 | 8.36E-06 |
| **MYH3** | -2.743102718 | 4.188005464 | 6.84E-08 | 8.48E-06 |
| **ANGPTL2** | -1.450109314 | 6.96627251 | 7.20E-08 | 8.83E-06 |
| **SCGB1A1** | -6.566778987 | 5.654305677 | 7.31E-08 | 8.87E-06 |
| **ANGPT1** | 1.421387536 | 5.050270187 | 7.90E-08 | 9.43E-06 |
| **MSX2** | 1.89225257 | 3.187284788 | 7.95E-08 | 9.43E-06 |
| **SEMA4B** | 1.139242247 | 4.928764374 | 8.26E-08 | 9.66E-06 |
| **NDUFB9** | 1.061890312 | 8.698323482 | 8.66E-08 | 9.96E-06 |
| **JAG2** | 1.195377954 | 4.883953759 | 8.83E-08 | 1.01E-05 |
| **CERKL** | 1.055704055 | 4.173520551 | 9.14E-08 | 1.03E-05 |
| **GABRG2** | 1.405222123 | 3.204725089 | 9.59E-08 | 1.07E-05 |
| **CHML** | 1.396799607 | 3.588241495 | 9.86E-08 | 1.09E-05 |
| **KCNK17** | 2.230953731 | 3.393441543 | 1.10E-07 | 1.19E-05 |
| **EFHD1** | 1.395615894 | 5.150796504 | 1.11E-07 | 1.19E-05 |
| **HBB** | 2.836890329 | 10.85817005 | 1.14E-07 | 1.19E-05 |
| **KLK4** | 1.80046288 | 4.255133916 | 1.15E-07 | 1.19E-05 |
| **BAMBI** | 1.43240349 | 6.822691618 | 1.15E-07 | 1.19E-05 |
| **CA12** | -2.031792607 | 4.689684283 | 1.27E-07 | 1.29E-05 |
| **GADD45B** | 1.125091021 | 6.363106475 | 1.27E-07 | 1.29E-05 |
| **GNG4** | 1.760702107 | 4.150570525 | 1.38E-07 | 1.39E-05 |
| **C4orf32** | 1.048454217 | 3.646509 | 1.40E-07 | 1.39E-05 |
| **CRYGS** | -1.905469334 | 3.912002147 | 1.45E-07 | 1.43E-05 |
| **DBNDD2** | 1.054015534 | 5.94229133 | 1.54E-07 | 1.50E-05 |
| **IFITM5** | 1.92404701 | 9.090616613 | 1.55E-07 | 1.50E-05 |
| **AMBN** | 3.471684624 | 6.4231731 | 1.61E-07 | 1.54E-05 |
| **CTXN1** | 1.870928069 | 4.640121659 | 1.64E-07 | 1.56E-05 |
| **IL11RA** | -1.332892112 | 4.535183931 | 1.69E-07 | 1.59E-05 |
| **C1R** | -1.341900738 | 7.570160567 | 1.71E-07 | 1.60E-05 |
| **IQCF3** | 1.782675211 | 3.530131707 | 1.77E-07 | 1.64E-05 |
| **CABP4** | 1.130867576 | 3.37446828 | 1.99E-07 | 1.81E-05 |
| **CD109** | -1.066861706 | 4.758197707 | 1.99E-07 | 1.81E-05 |
| **ECM1** | -1.208568985 | 5.615953684 | 2.00E-07 | 1.81E-05 |
| **URI1** | 1.488392932 | 7.269060466 | 2.01E-07 | 1.81E-05 |
| **CHN2** | 1.046477185 | 4.49524446 | 2.27E-07 | 2.00E-05 |
| **FGGY** | -1.206184451 | 4.220329977 | 2.51E-07 | 2.20E-05 |
| **C19orf12** | 1.404311558 | 5.92479875 | 2.54E-07 | 2.21E-05 |
| **FBLN7** | -1.275550096 | 4.4111432 | 2.76E-07 | 2.37E-05 |
| **C11orf70** | 1.336099424 | 3.221461782 | 2.77E-07 | 2.37E-05 |
| **MATN4** | -3.505734067 | 5.413458137 | 2.99E-07 | 2.54E-05 |
| **IGSF10** | -1.450331993 | 4.076307924 | 3.01E-07 | 2.54E-05 |
| **MAN1A1** | -1.080835664 | 3.822263916 | 3.10E-07 | 2.59E-05 |
| **SAMD11** | 1.335185271 | 5.718059756 | 3.88E-07 | 3.22E-05 |
| **NSG1** | 1.74829296 | 3.505531155 | 4.45E-07 | 3.66E-05 |
| **DLX4** | 1.494079179 | 4.903843172 | 4.56E-07 | 3.73E-05 |
| **CH25H** | -1.80849429 | 3.250379928 | 5.35E-07 | 4.22E-05 |
| **RNASE4** | -1.135450257 | 4.427268188 | 5.61E-07 | 4.39E-05 |
| **S100A16** | -1.181990266 | 6.215549131 | 5.72E-07 | 4.43E-05 |
| **TRIM17** | 1.479655088 | 3.090407186 | 5.74E-07 | 4.43E-05 |
| **COL8A2** | -1.831457206 | 5.467630104 | 6.29E-07 | 4.79E-05 |
| **MFAP4** | -2.796568071 | 6.616149802 | 6.65E-07 | 4.96E-05 |
| **CWF19L2** | 1.308462707 | 4.7307108 | 6.96E-07 | 5.12E-05 |
| **TCF7L2** | 1.471400088 | 5.060201502 | 7.08E-07 | 5.18E-05 |
| **PTGFR** | -1.624612768 | 4.314972111 | 7.47E-07 | 5.39E-05 |
| **MEGF6** | -1.610516712 | 3.493232132 | 7.57E-07 | 5.40E-05 |
| **GAS1** | -1.308043047 | 5.318553426 | 7.62E-07 | 5.40E-05 |
| **CCNE1** | 1.682809215 | 5.520201 | 7.88E-07 | 5.54E-05 |
| **HBA2** | 2.674870995 | 11.20365588 | 7.95E-07 | 5.55E-05 |
| **TBXA2R** | 1.148300635 | 3.743723795 | 8.35E-07 | 5.76E-05 |
| **COL22A1** | 1.955101572 | 5.760463702 | 8.60E-07 | 5.86E-05 |
| **PGF** | 1.108643343 | 6.00616792 | 8.80E-07 | 5.93E-05 |
| **COL6A2** | -1.108052751 | 9.992217656 | 8.80E-07 | 5.93E-05 |
| **THBS3** | -1.141059572 | 5.574587924 | 9.01E-07 | 6.03E-05 |
| **COL6A1** | -1.1034045 | 10.01193849 | 9.30E-07 | 6.19E-05 |
| **NETO2** | 1.491855663 | 4.994640993 | 9.97E-07 | 6.55E-05 |
| **P4HA3** | -1.400720712 | 4.094594749 | 1.01E-06 | 6.63E-05 |
| **MICAL2** | -1.006148856 | 4.848473739 | 1.05E-06 | 6.81E-05 |
| **FMO1** | -2.88830993 | 3.276440747 | 1.05E-06 | 6.81E-05 |
| **HES4** | 1.114058485 | 6.249208161 | 1.09E-06 | 6.98E-05 |
| **GRAMD1B** | 1.093290723 | 4.037058788 | 1.18E-06 | 7.47E-05 |
| **EGFL6** | -2.581522526 | 5.965629663 | 1.22E-06 | 7.66E-05 |
| **DAPL1** | 2.02716703 | 3.932917754 | 1.45E-06 | 8.94E-05 |
| **SFTPA2** | -6.232166594 | 4.462840184 | 1.49E-06 | 9.08E-05 |
| **HBA1** | 2.441692796 | 10.69478087 | 1.58E-06 | 9.60E-05 |
| **ALDH1A1** | -1.885712825 | 4.217052528 | 1.62E-06 | 9.77E-05 |
| **FBXO15** | 1.40069351 | 3.887412209 | 1.75E-06 | 0.000104054 |
| **CA5B** | -1.161188416 | 4.005615032 | 1.76E-06 | 0.000104054 |
| **THBS2** | -1.384671518 | 6.859244593 | 1.76E-06 | 0.000104054 |
| **BAHCC1** | 1.385323844 | 4.106109307 | 1.94E-06 | 0.000113046 |
| **MYEF2** | 1.289625083 | 3.866572193 | 2.10E-06 | 0.000120237 |
| **ZIC1** | -1.169554187 | 4.581041845 | 2.18E-06 | 0.00012363 |
| **AIM2** | -1.638117973 | 3.421920725 | 2.23E-06 | 0.0001255 |
| **AHCYL2** | 1.268041169 | 4.385288335 | 2.32E-06 | 0.000129737 |
| **NTN4** | -1.286927599 | 3.203078645 | 2.45E-06 | 0.00013484 |
| **ITGA11** | -1.205029938 | 5.202307206 | 2.46E-06 | 0.00013484 |
| **WIF1** | 2.284924319 | 4.866562074 | 2.49E-06 | 0.00013599 |
| **OSTN** | -3.399659825 | 3.836054874 | 2.54E-06 | 0.000138099 |
| **KAZALD1** | 1.150094616 | 6.593029459 | 2.58E-06 | 0.00013914 |
| **RHBDL2** | 1.238674803 | 6.047048426 | 2.60E-06 | 0.00013914 |
| **CDK18** | 1.006691022 | 4.7872887 | 2.61E-06 | 0.00013914 |
| **RAB11FIP1** | 1.045429105 | 3.457500895 | 2.63E-06 | 0.00013979 |
| **HMCN1** | -1.125396066 | 4.754976468 | 2.66E-06 | 0.000140458 |
| **WNT5B** | -1.299295951 | 4.451380158 | 2.90E-06 | 0.000151363 |
| **ZNF362** | 1.383263689 | 5.989918701 | 2.94E-06 | 0.000151693 |
| **NPB** | 1.625723438 | 4.966748534 | 3.48E-06 | 0.000172215 |
| **RPL22L1** | 1.082078638 | 6.177188196 | 3.71E-06 | 0.000181986 |
| **PDCD2L** | 1.103017221 | 3.955745584 | 3.98E-06 | 0.000194509 |
| **FLNC** | -1.874039633 | 4.352965098 | 4.03E-06 | 0.000195652 |
| **PHLDB2** | -1.131117743 | 3.908530299 | 4.05E-06 | 0.000195652 |
| **PLPP4** | 1.426526645 | 3.457116337 | 4.28E-06 | 0.000202599 |
| **FOSB** | 2.158746796 | 4.990944396 | 4.35E-06 | 0.000205138 |
| **TNFRSF6B** | -2.111651533 | 4.405647863 | 4.46E-06 | 0.000207663 |
| **CRIP1** | 1.191816683 | 8.61438196 | 4.70E-06 | 0.000217089 |
| **SFTPA1** | -5.887986769 | 4.015526513 | 4.83E-06 | 0.000221266 |
| **ADAM32** | 1.151279843 | 3.673847727 | 5.05E-06 | 0.000229458 |
| **NR2F1** | -1.09869719 | 3.542668565 | 5.17E-06 | 0.000232523 |
| **CAMK2N1** | -1.454912573 | 4.014896175 | 5.41E-06 | 0.000239809 |
| **ADRA1D** | 1.49250582 | 3.812357203 | 5.47E-06 | 0.000239904 |
| **FBLN5** | -1.348484603 | 4.597687186 | 5.52E-06 | 0.000240987 |
| **PTN** | -1.550868989 | 8.262160309 | 5.67E-06 | 0.000245335 |
| **SFTPB** | -3.496138359 | 4.81610505 | 6.10E-06 | 0.00025991 |
| **CMIP** | -1.025099806 | 5.715285397 | 6.15E-06 | 0.000261274 |
| **TSPAN7** | -1.091108736 | 5.841034497 | 6.21E-06 | 0.000262755 |
| **XYLT1** | -1.113156175 | 3.260751274 | 6.33E-06 | 0.000265572 |
| **GLTSCR1** | 1.188128003 | 4.253274599 | 6.43E-06 | 0.000268827 |
| **PRRX1** | -1.063399447 | 7.246442919 | 6.74E-06 | 0.000278547 |
| **DMP1** | 1.982659083 | 5.222880325 | 6.86E-06 | 0.00028053 |
| **SSPN** | -1.108179677 | 5.202229321 | 7.21E-06 | 0.000291383 |
| **NR1D1** | -1.215064323 | 3.923701262 | 7.34E-06 | 0.000295876 |
| **VSIG10** | 1.062035278 | 4.309365768 | 8.29E-06 | 0.000325626 |
| **TMEM145** | 1.599096976 | 3.185364345 | 8.35E-06 | 0.000325626 |
| **HEY1** | 1.103958673 | 5.867239579 | 8.38E-06 | 0.000325626 |
| **SSX4** | 2.886431348 | 3.434210181 | 8.43E-06 | 0.000326431 |
| **OR4F17** | 1.435291739 | 4.048990047 | 9.07E-06 | 0.000346336 |
| **SFTPC** | -5.622643177 | 6.393411602 | 9.21E-06 | 0.000350688 |
| **PYGM** | 1.504826656 | 3.950979516 | 1.01E-05 | 0.00037846 |
| **FBN1** | -1.009609475 | 6.421815907 | 1.03E-05 | 0.000382313 |
| **NID2** | -1.014259876 | 6.176761305 | 1.03E-05 | 0.000382813 |
| **MEST** | -1.64772933 | 5.559848019 | 1.06E-05 | 0.000388639 |
| **POSTN** | -1.255723692 | 8.343587165 | 1.07E-05 | 0.000393693 |
| **CAV1** | -1.094445715 | 5.940501965 | 1.08E-05 | 0.000393693 |
| **RAMP1** | 1.318347549 | 4.798453773 | 1.08E-05 | 0.000393693 |
| **GEM** | -1.294517816 | 4.035449994 | 1.18E-05 | 0.000428593 |
| **ACTA2** | -1.178338322 | 7.439307196 | 1.22E-05 | 0.000438646 |
| **ADGRD1** | 1.244114995 | 4.405577513 | 1.49E-05 | 0.000516699 |
| **CDH15** | 1.400198209 | 6.140873414 | 1.50E-05 | 0.000519276 |
| **WRAP53** | 1.581030067 | 5.021113592 | 1.56E-05 | 0.000533449 |
| **USP28** | 1.146931932 | 4.888002988 | 1.56E-05 | 0.000533449 |
| **EFHC1** | 1.040104019 | 4.929066855 | 1.64E-05 | 0.000554794 |
| **LUM** | -1.012794116 | 11.46104548 | 1.71E-05 | 0.000565279 |
| **SLC14A1** | -2.06616069 | 3.211983002 | 1.76E-05 | 0.000577592 |
| **LOXL1** | -1.288921129 | 6.852731732 | 1.83E-05 | 0.000597451 |
| **NTNG1** | -1.463630387 | 3.364915414 | 1.94E-05 | 0.000621445 |
| **RASL11B** | -1.298826559 | 3.360439991 | 1.96E-05 | 0.00062345 |
| **PPP1R14A** | -1.535703299 | 3.594938331 | 1.98E-05 | 0.000628954 |
| **SLC36A2** | 1.253847507 | 3.80705941 | 2.05E-05 | 0.000643028 |
| **CA3** | 1.793148537 | 7.491557285 | 2.12E-05 | 0.000654597 |
| **SLC8A3** | 1.42791303 | 4.74971859 | 2.15E-05 | 0.000660003 |
| **MOSPD2** | 1.082929488 | 4.399051814 | 2.16E-05 | 0.000660977 |
| **GPI** | 1.003727741 | 8.504280382 | 2.43E-05 | 0.000731023 |
| **ANO5** | 1.286168347 | 5.232574536 | 2.43E-05 | 0.000731023 |
| **TMEM212** | 1.241826788 | 3.350324835 | 2.46E-05 | 0.000735486 |
| **APCDD1L** | -1.818368617 | 3.484929325 | 2.52E-05 | 0.000745914 |
| **PHOSPHO1** | 1.345125809 | 7.093746793 | 2.76E-05 | 0.000801664 |
| **ABCB4** | 1.13613117 | 3.448883786 | 2.76E-05 | 0.000801664 |
| **SAP25** | 1.825917358 | 3.159229648 | 2.77E-05 | 0.000801664 |
| **FNDC5** | -1.555705119 | 3.451241414 | 2.94E-05 | 0.000842003 |
| **INTU** | 1.019570356 | 3.706604554 | 3.03E-05 | 0.000863163 |
| **S100A2** | -1.288802252 | 4.558933217 | 3.05E-05 | 0.000863163 |
| **SAMD5** | -1.209091424 | 3.059687725 | 3.18E-05 | 0.000896817 |
| **POPDC3** | -1.422257801 | 3.33598874 | 3.30E-05 | 0.000922642 |
| **LRIG1** | -1.374201244 | 3.440974981 | 3.36E-05 | 0.0009345 |
| **CPNE5** | 1.069160213 | 4.098813719 | 3.41E-05 | 0.0009448 |
| **RTN1** | 1.028914852 | 3.31108477 | 3.72E-05 | 0.001012008 |
| **EPHA3** | -1.038766503 | 4.394853664 | 3.92E-05 | 0.001052139 |
| **HMGA2** | -1.730870895 | 3.243259864 | 3.94E-05 | 0.001055502 |
| **MMP1** | -1.664564986 | 3.348377036 | 4.07E-05 | 0.001080234 |
| **CFB** | -1.088023904 | 3.245847748 | 4.18E-05 | 0.001097453 |
| **SCGB3A2** | -1.871504771 | 4.587712194 | 4.19E-05 | 0.001097453 |
| **CILP2** | -1.774475453 | 4.142535858 | 4.21E-05 | 0.001100769 |
| **RGS4** | -1.558681374 | 3.135798025 | 4.28E-05 | 0.001112903 |
| **ECM2** | -1.1665242 | 4.538911918 | 4.39E-05 | 0.00113328 |
| **PLSCR4** | -1.009874818 | 3.663885317 | 4.53E-05 | 0.001162129 |
| **TG** | 1.085260693 | 3.554032491 | 4.67E-05 | 0.00119508 |
| **DIO2** | 1.343794785 | 4.964811578 | 5.08E-05 | 0.00128545 |
| **CRYBA2** | 2.129752937 | 3.054662719 | 5.10E-05 | 0.001287666 |
| **S100A8** | 2.02888266 | 4.936192482 | 5.82E-05 | 0.001421397 |
| **RAB27B** | -1.000752345 | 3.082894221 | 6.18E-05 | 0.001487093 |
| **GYG2** | 1.007003297 | 5.149520153 | 6.72E-05 | 0.001601137 |
| **FAT3** | 1.046860162 | 5.484633887 | 7.38E-05 | 0.001729014 |
| **SLC6A15** | 1.548919612 | 3.494250049 | 7.50E-05 | 0.001745694 |
| **SMOC1** | -1.572127846 | 3.845280055 | 7.73E-05 | 0.001785928 |
| **TMSB15A** | 1.189200671 | 6.197229187 | 7.78E-05 | 0.001790384 |
| **BMP8B** | 1.108022641 | 4.923027277 | 8.38E-05 | 0.001902771 |
| **COL24A1** | 1.049520878 | 4.379272207 | 8.45E-05 | 0.001910393 |
| **WISP2** | -1.481126202 | 4.151811885 | 8.66E-05 | 0.001955199 |
| **C16orf89** | -1.4523509 | 3.229634181 | 8.83E-05 | 0.001982226 |
| **FGFR2** | -1.022080616 | 5.409403079 | 8.85E-05 | 0.001982226 |
| **HEPH** | -1.474628221 | 3.477803061 | 9.20E-05 | 0.002038931 |
| **CXADR** | 1.107465575 | 3.375819216 | 9.87E-05 | 0.002152973 |
| **NAA38** | 1.139609898 | 7.891578988 | 0.000108644 | 0.002312276 |
| **SOD3** | -1.262859418 | 5.716134945 | 0.000108712 | 0.002312276 |
| **ALPL** | 1.095354412 | 9.932061682 | 0.000114329 | 0.00239029 |
| **COL9A1** | -2.562564039 | 4.99435608 | 0.000118386 | 0.002442726 |
| **CGREF1** | 1.157494318 | 5.718455988 | 0.000126554 | 0.002582308 |
| **EMID1** | -1.081840029 | 4.474303694 | 0.000127222 | 0.002587858 |
| **SULF2** | -1.009670597 | 5.268108334 | 0.000129829 | 0.002634521 |
| **AQP3** | -1.181754628 | 3.307010935 | 0.000131428 | 0.002657214 |
| **CNBD2** | 1.111152482 | 3.2490958 | 0.000132504 | 0.002669182 |
| **NOTUM** | 1.438159682 | 4.839821388 | 0.000139623 | 0.00273765 |
| **PDGFD** | 1.018701024 | 5.348947476 | 0.000144694 | 0.002792448 |
| **C10orf10** | -1.240853371 | 5.025472525 | 0.000161684 | 0.003082611 |
| **FOSL1** | -1.107193627 | 4.294268875 | 0.00016495 | 0.003123299 |
| **ARG2** | -1.00048696 | 3.506392173 | 0.000170597 | 0.00319731 |
| **TGFBI** | -1.037603532 | 10.16685913 | 0.000182319 | 0.00336285 |
| **LOXL4** | 1.063899153 | 3.732598796 | 0.000189372 | 0.003478333 |
| **ADAP1** | 1.056485149 | 3.317823719 | 0.000194154 | 0.003548405 |
| **GFPT2** | -1.104040973 | 3.701977662 | 0.000199453 | 0.003600962 |
| **IBSP** | 1.36073694 | 10.29245859 | 0.000199637 | 0.003600962 |
| **PENK** | 1.974273715 | 3.284498265 | 0.000207233 | 0.003689753 |
| **CHRNA1** | -1.863044769 | 4.14897386 | 0.00022034 | 0.003842546 |
| **SEMA3C** | -1.160128741 | 3.653671033 | 0.000222002 | 0.00385934 |
| **EPCAM** | 1.301589085 | 3.769176307 | 0.000273623 | 0.004562721 |
| **SPP2** | 1.240040512 | 3.184593532 | 0.000296543 | 0.004821111 |
| **NDNF** | 1.064252386 | 5.509350652 | 0.00030491 | 0.004913733 |
| **NTM** | 1.634009844 | 4.538715865 | 0.000309936 | 0.004972943 |
| **GSTA1** | 1.941581925 | 5.075225396 | 0.000317767 | 0.005018348 |
| **GLIS3** | -1.101284548 | 3.06177833 | 0.000352845 | 0.00547739 |
| **RARRES2** | -1.663872813 | 5.389138185 | 0.000355121 | 0.005482773 |
| **AHNAK2** | -1.012713731 | 3.679649157 | 0.000357485 | 0.005496207 |
| **MSC** | -1.130113152 | 4.24332564 | 0.000360973 | 0.005534418 |
| **ENTPD3** | 1.230021152 | 3.925952316 | 0.00039699 | 0.006003249 |
| **ISLR** | -1.698044907 | 6.393160809 | 0.000414963 | 0.006203446 |
| **COL27A1** | -1.037598591 | 5.482599713 | 0.000417222 | 0.006207167 |
| **GAL** | 1.518779481 | 4.372569035 | 0.000449471 | 0.006532273 |
| **ASPN** | -1.531795133 | 6.414111132 | 0.000479275 | 0.006844399 |
| **SLAMF9** | -1.974207917 | 3.19171327 | 0.000483712 | 0.006889387 |
| **CA9** | -1.682532584 | 4.519667956 | 0.000548973 | 0.007537455 |
| **STAC3** | -1.378721525 | 4.523138856 | 0.000579668 | 0.007822682 |
| **CHST13** | 1.049482688 | 3.79877704 | 0.000588057 | 0.007878108 |
| **TCEAL7** | -1.189411582 | 3.133608745 | 0.000594297 | 0.007942421 |
| **GALNT14** | 1.020390675 | 5.129303378 | 0.000597519 | 0.007975832 |
| **COL2A1** | -2.024361774 | 7.853481125 | 0.000708188 | 0.009057483 |
| **MLC1** | 1.225712925 | 4.325324218 | 0.000750688 | 0.009492371 |
| **HGF** | -1.135028997 | 3.616974968 | 0.000874325 | 0.010676631 |
| **TTLL7** | 1.295079043 | 3.982870034 | 0.000892272 | 0.010823943 |
| **CHRDL2** | -1.897188199 | 4.473403006 | 0.001012769 | 0.011805654 |
| **DPPA4** | 1.365105552 | 3.746917805 | 0.001063492 | 0.012252312 |
| **FGFR4** | -1.43592526 | 3.431896979 | 0.001078297 | 0.012365315 |
| **RYR1** | -1.246385876 | 3.356391928 | 0.001085828 | 0.012412874 |
| **GZMA** | 1.228610996 | 3.894838127 | 0.001101085 | 0.012517892 |
| **ETV1** | -1.048501556 | 3.27516245 | 0.001127956 | 0.012731596 |
| **TNNT3** | -1.56708275 | 5.519668307 | 0.001226573 | 0.01347277 |
| **LY6K** | 1.067397049 | 5.396817364 | 0.001295342 | 0.014046449 |
| **MFI2** | 1.137273325 | 6.768080904 | 0.001323541 | 0.014256309 |
| **ACAN** | -1.35330514 | 8.169092507 | 0.001495769 | 0.015466393 |
| **COL11A1** | -1.041354634 | 9.154520328 | 0.001670219 | 0.016656135 |
| **DLK2** | -1.050266652 | 3.073798922 | 0.00172849 | 0.017128184 |
| **GAGE12F** | 2.344255971 | 5.694554546 | 0.001913905 | 0.018441198 |
| **SRPK3** | -1.046884459 | 3.445189015 | 0.002212295 | 0.020266828 |
| **S100A9** | 1.353005538 | 5.688861707 | 0.002285435 | 0.020747462 |
| **TP53** | 1.600904509 | 8.873616411 | 0.002538751 | 0.022277641 |
| **MMP23B** | -1.106950751 | 4.743610733 | 0.00259998 | 0.022670758 |
| **DAZ1** | 2.253097576 | 3.757506748 | 0.002691398 | 0.023156928 |
| **SCUBE3** | -1.156337761 | 3.583986873 | 0.003076228 | 0.025652244 |
| **CST7** | -1.069371438 | 3.210899121 | 0.003082044 | 0.025652244 |
| **MT1A** | 1.130816773 | 4.135095539 | 0.003400935 | 0.027430602 |
| **COL11A2** | 1.124724623 | 7.714035516 | 0.003483537 | 0.027807151 |
| **GAGE2A** | 2.266936374 | 3.538747364 | 0.003879451 | 0.030222488 |
| **CAMK2B** | 1.041542239 | 3.419180145 | 0.004932038 | 0.035320993 |
| **KCNJ3** | 1.165071647 | 3.261906002 | 0.004933868 | 0.035320993 |
| **PTGDS** | -1.207340228 | 7.062403971 | 0.005429866 | 0.037698297 |
| **SCT** | 1.038701758 | 3.456788124 | 0.005590352 | 0.038360403 |
| **IGF2** | -1.091670404 | 8.226558974 | 0.006423696 | 0.041984123 |
| **CCND2** | -1.046995592 | 4.518245156 | 0.007164933 | 0.04496515 |
| **SCRG1** | -1.216171652 | 3.361455481 | 0.007435548 | 0.046294987 |
| **PCSK1N** | 1.152866736 | 3.156219439 | 0.007698461 | 0.047454451 |
