# Supplementary material for: Low GNG12 Expression Predicts Adverse Outcomes: A Potential Therapeutic Target for Osteosarcoma
Source: Front Immunol. 2021 Oct 6;12:758845. doi: 10.3389/fimmu.2021.758845 (PMC8527884; doi:10.3389/fimmu.2021.758845)

No.1 H-Score 46.668


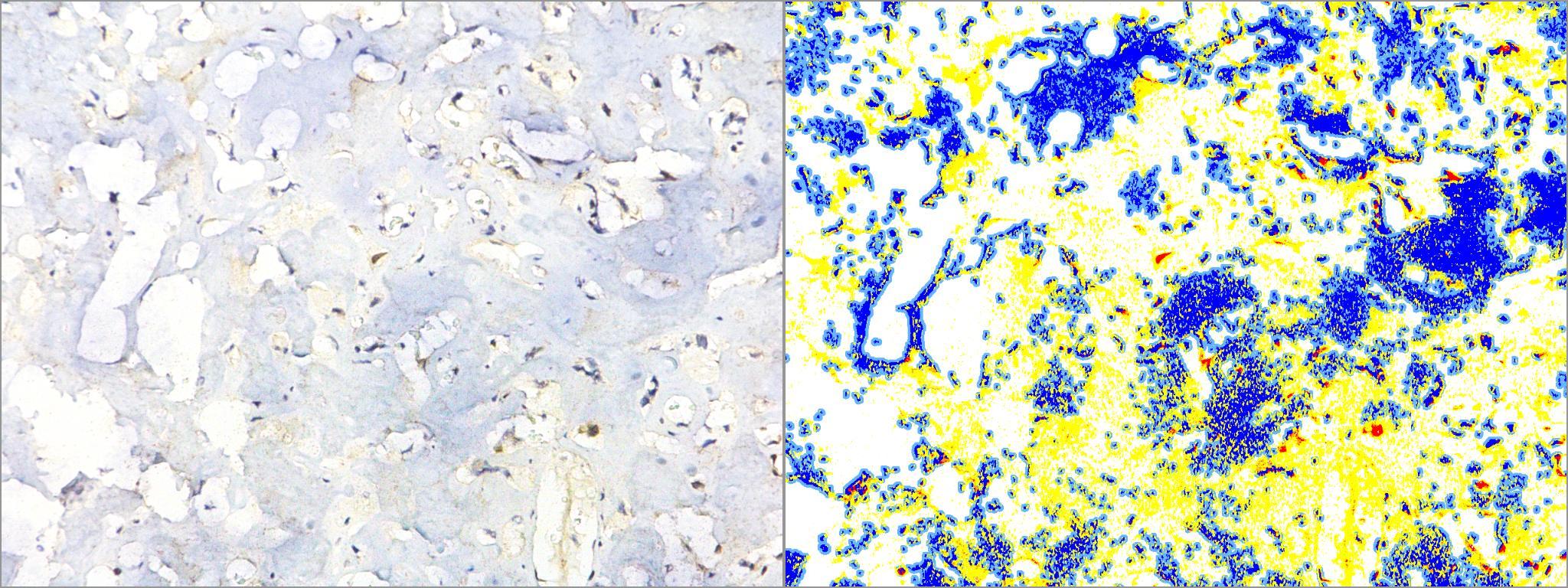


No.2 H-Score 75.116


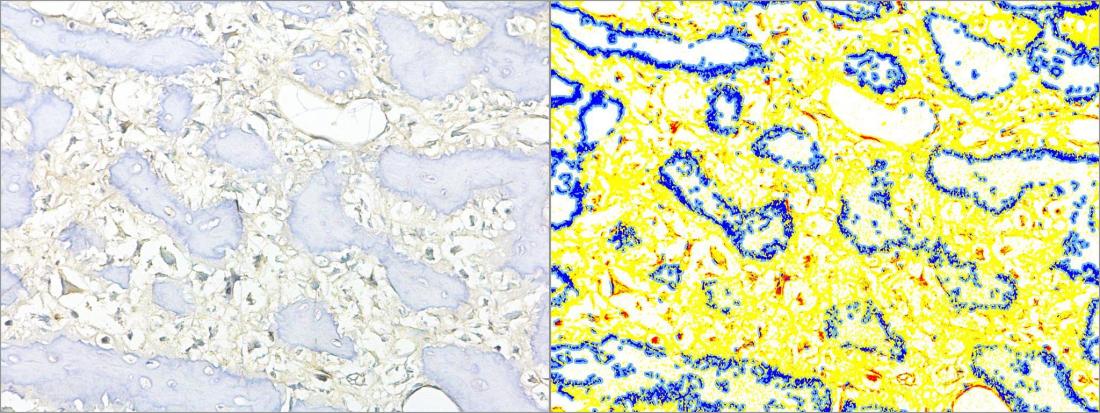


No.3 H-Score 116.12


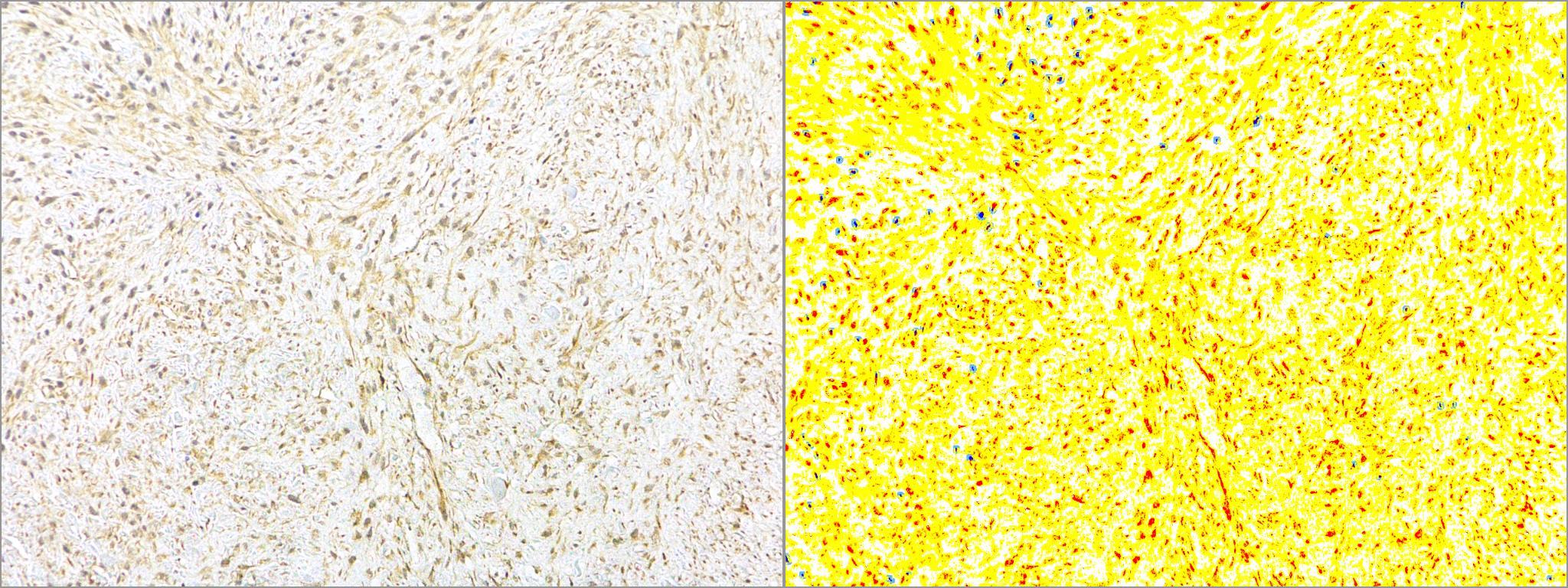


No.4 H-Score 130.89


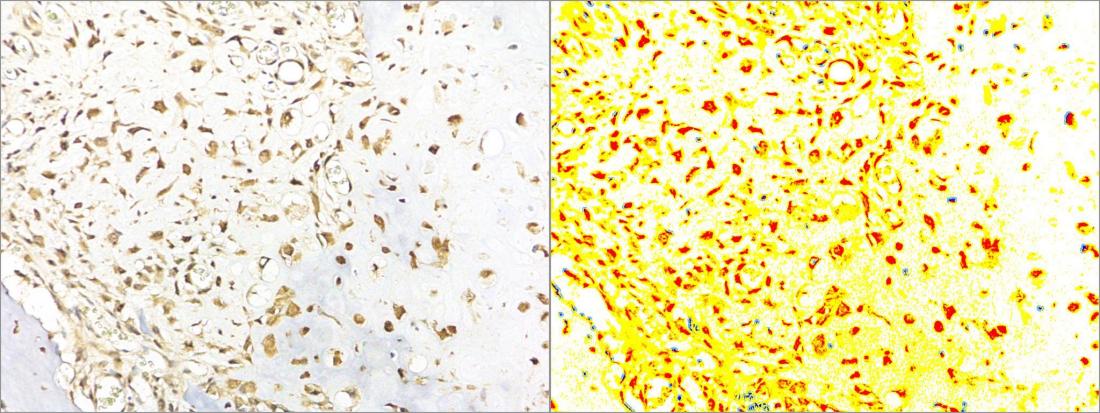


No.5 H-Score 97.705


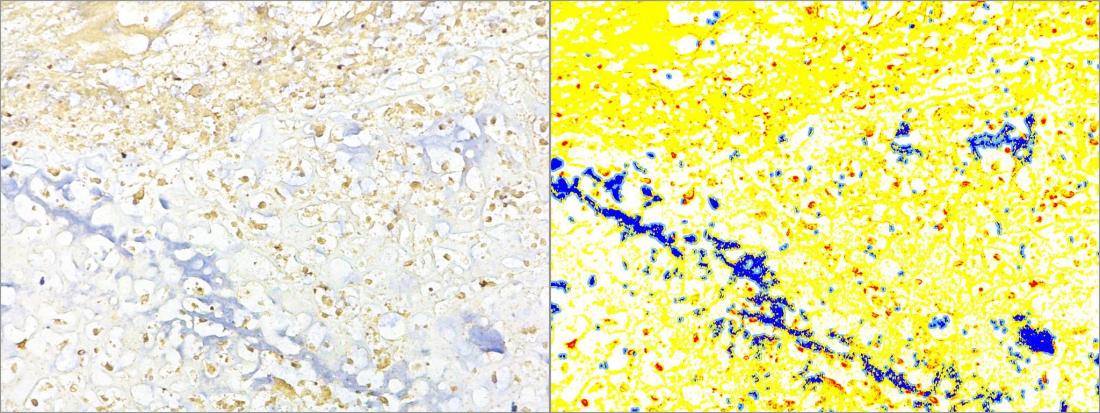


No.6 H-Score 111.631


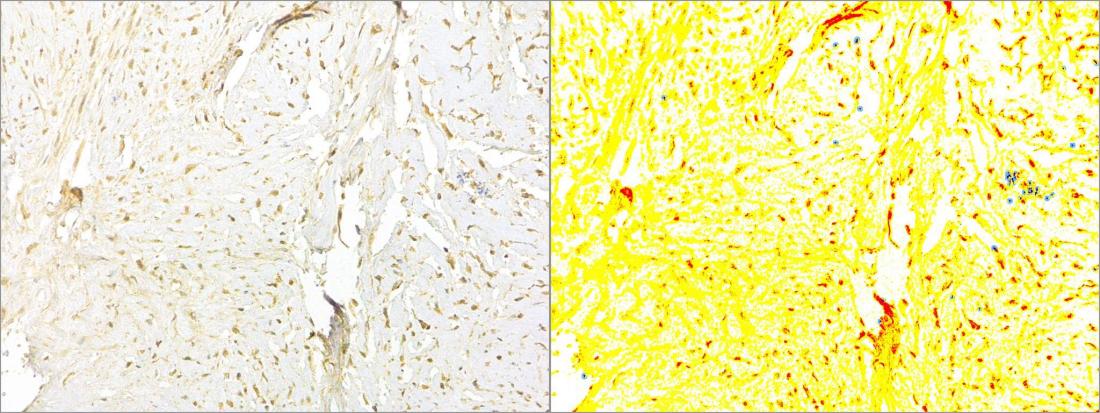


No.7 H-Score 84.363


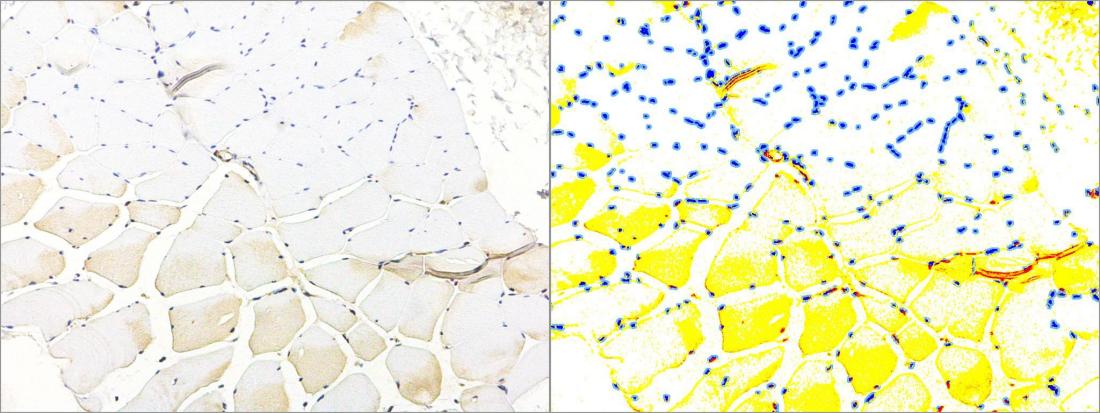


No.8 H-Score 111.986


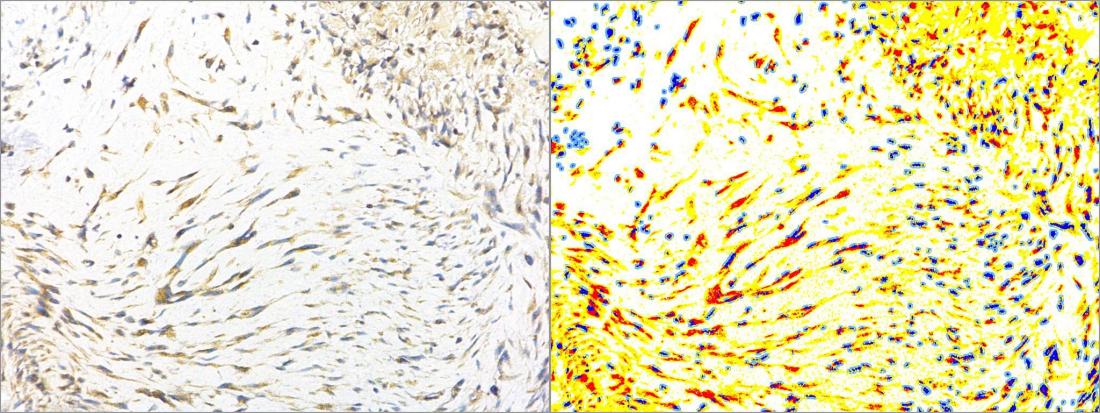


No.9 H-Score 19.265


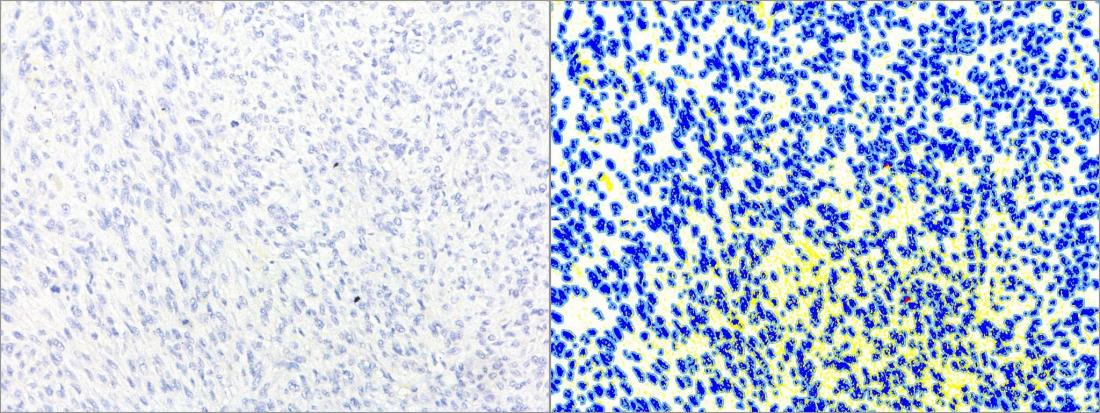


No.10 H-Score 96.019


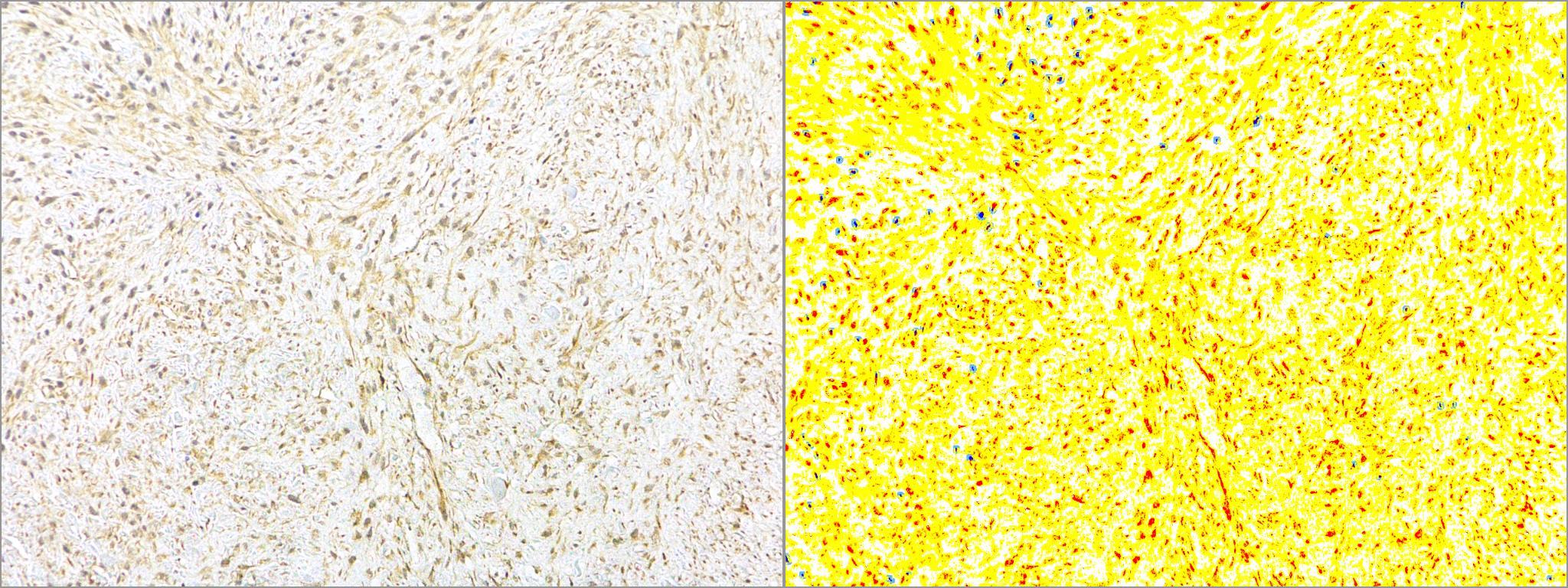


No.11 H-Score 114.5


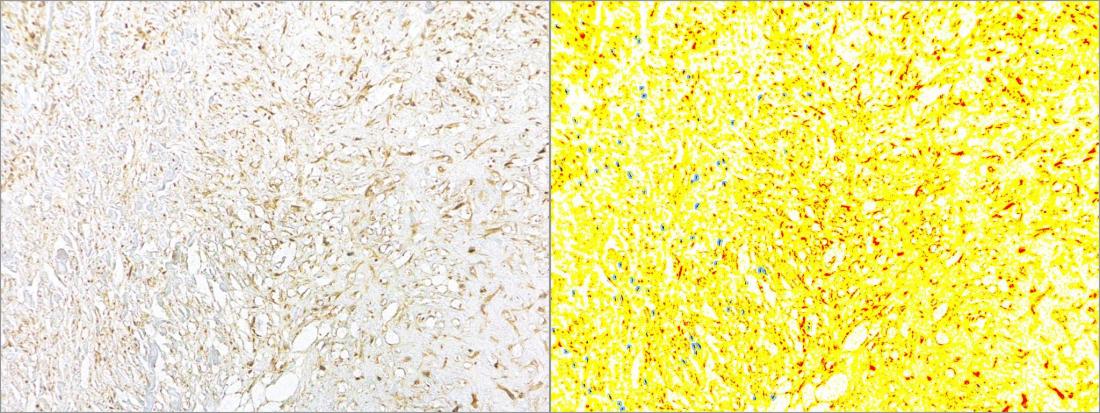


No.12 H-Score 126.147


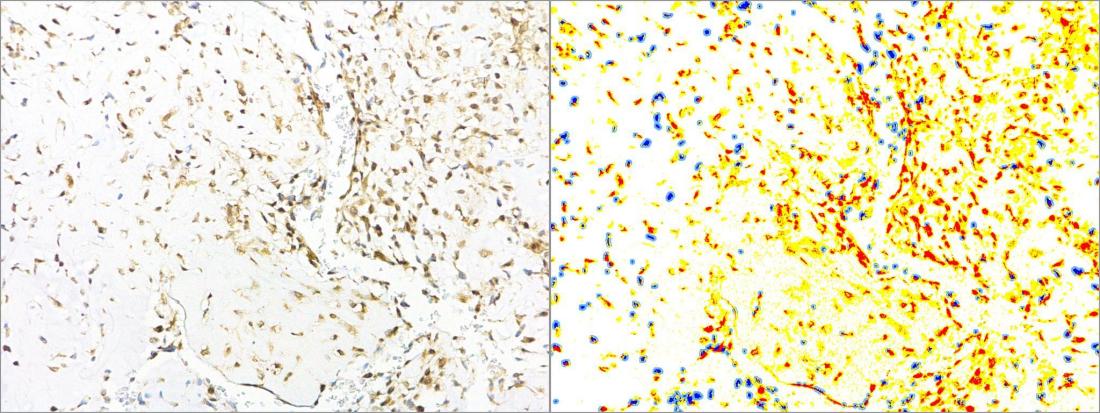


No.13 H-Score 107.447


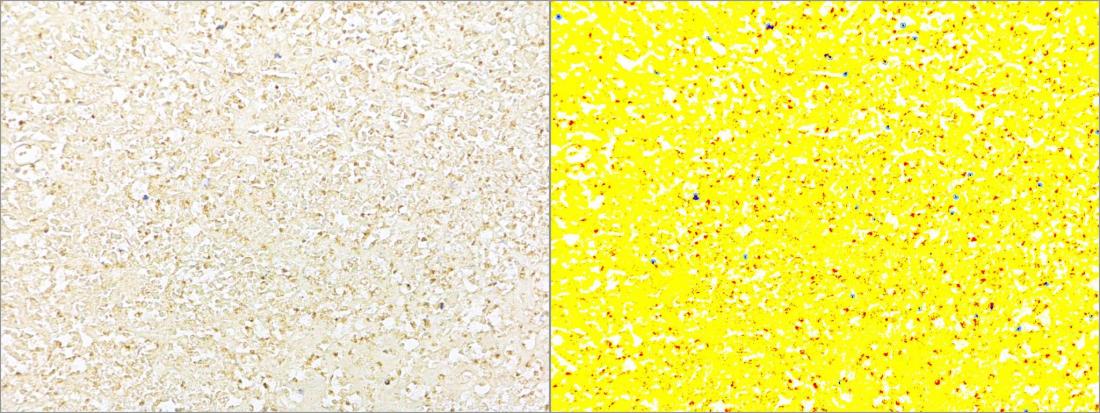


No.14 H-Score 65.701


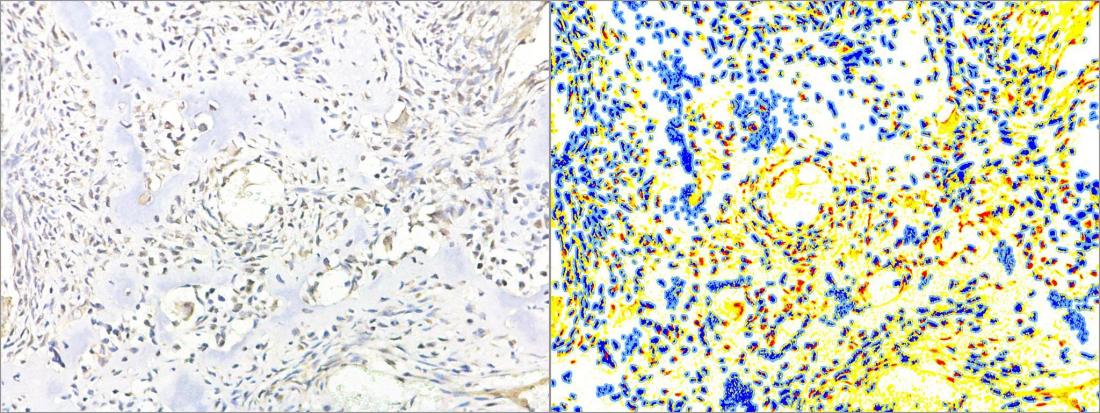


No.15 H-Score 130.523


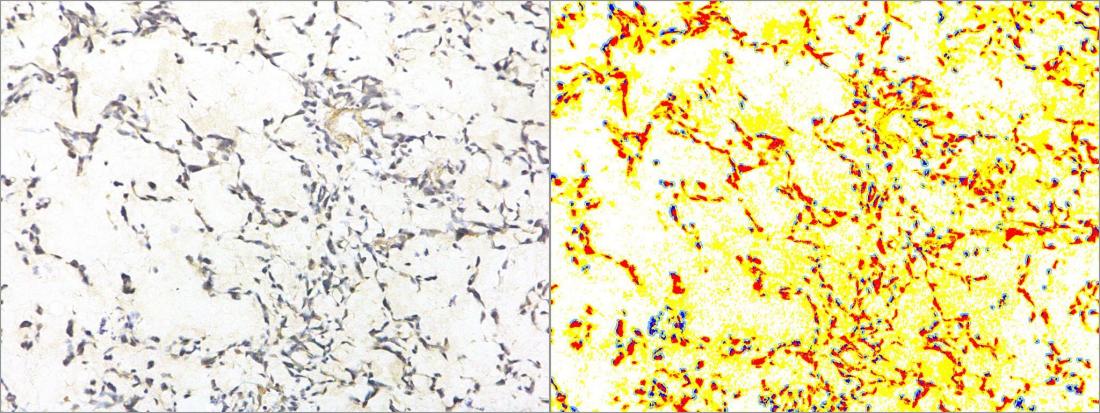


No.16 H-Score 103.692


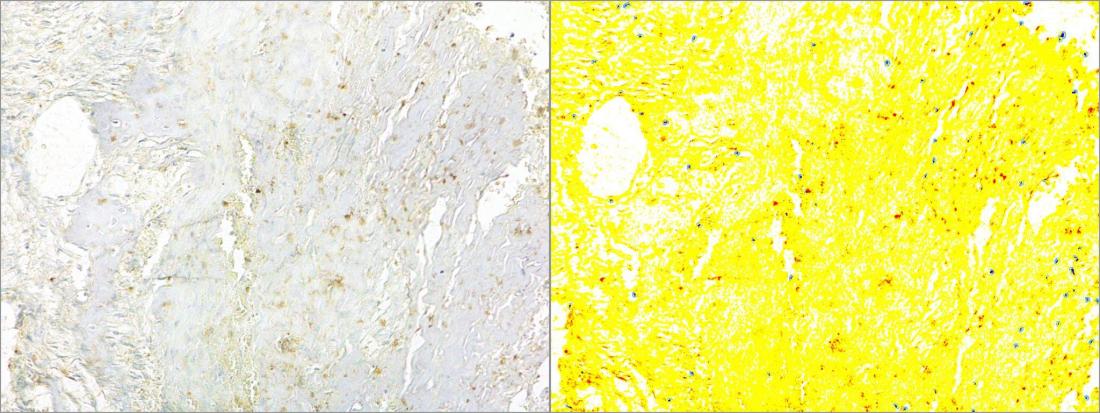


No.17 H-Score 99.564


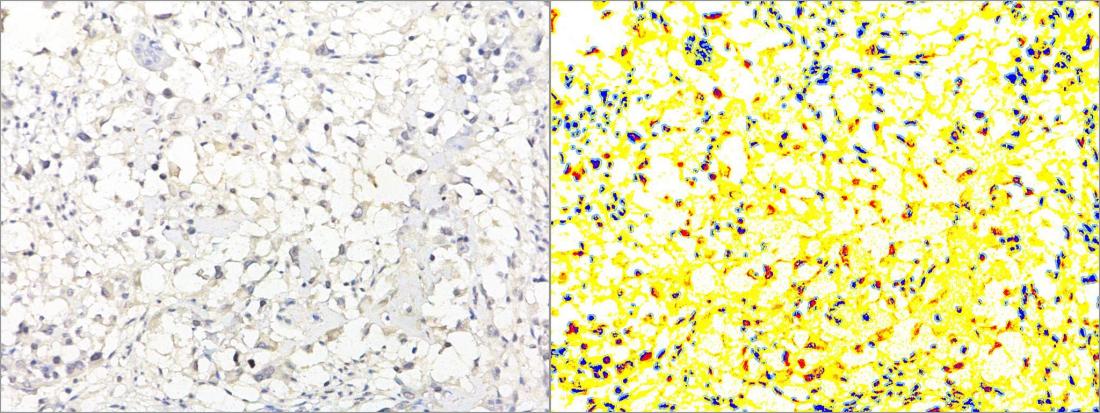


No.18 H-Score 84.915


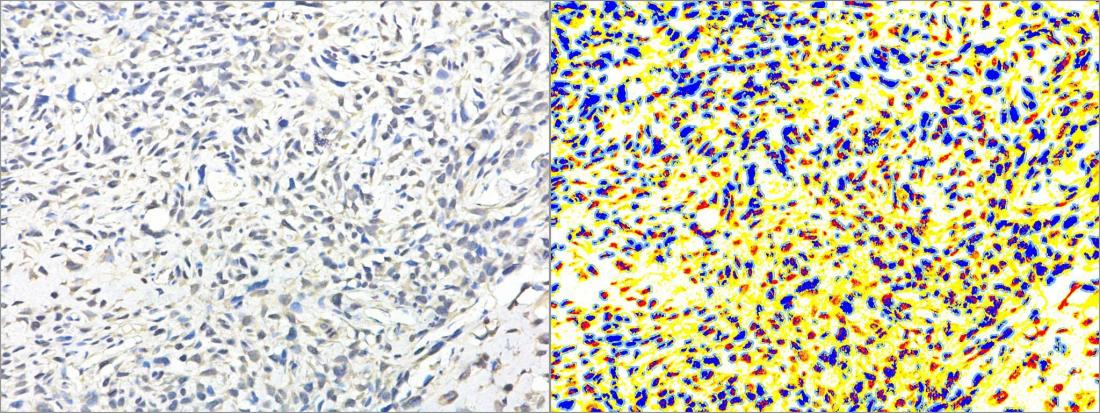


No.19 H-Score 104.892


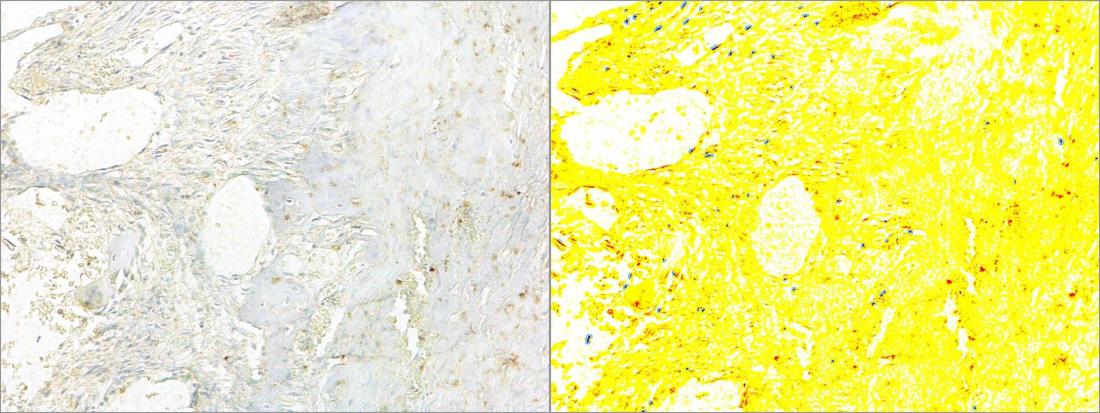


No.20 H-Score 102.54


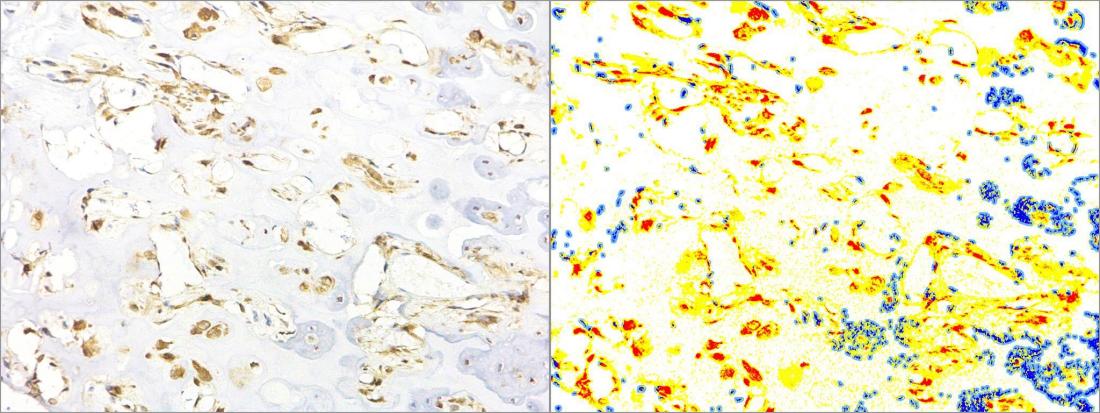


No.21 H-Score 61.021


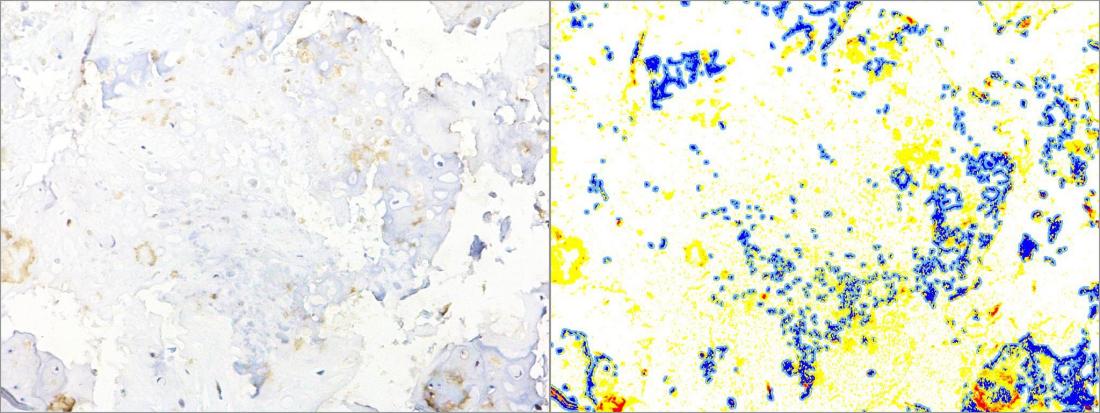


No.22 H-Score 107.991


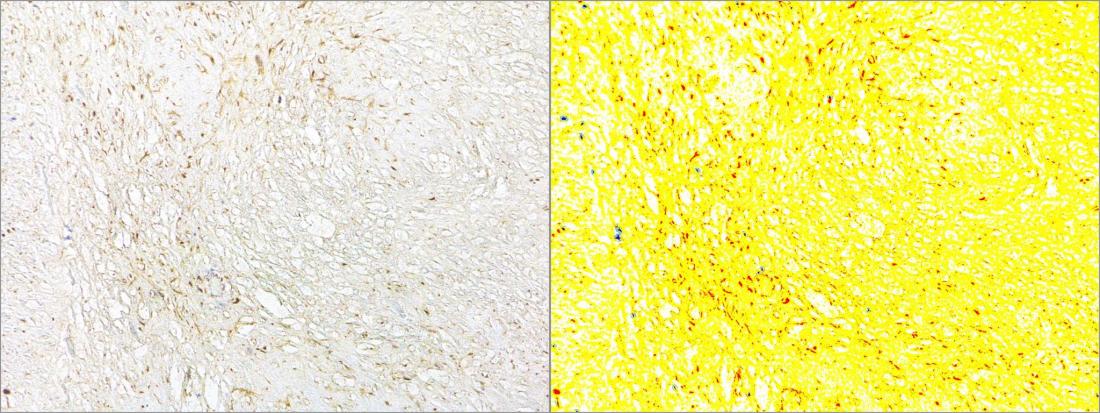


No.23 H-Score 106.326


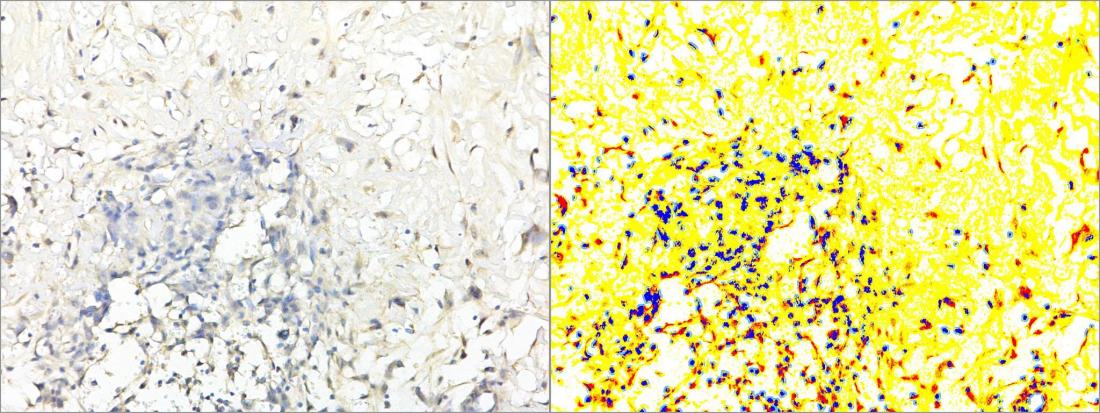


No.24 H-Score 63.257


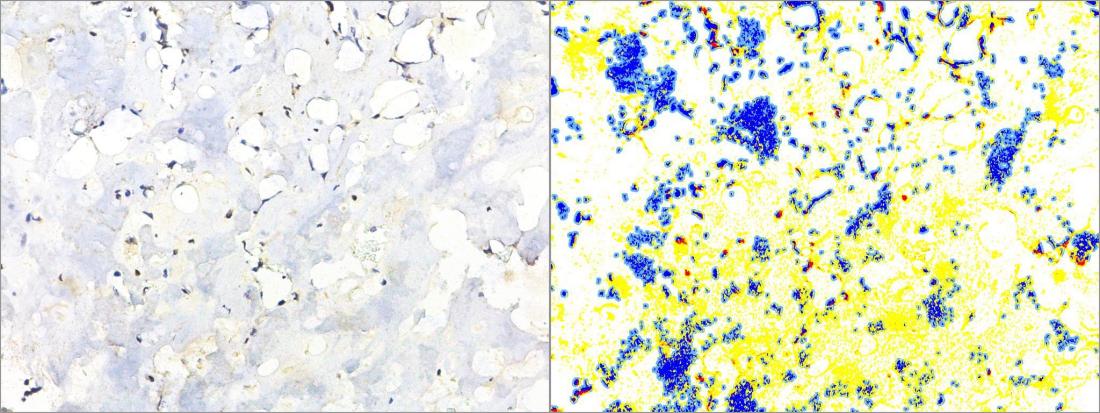


No.25 H-Score 86.386


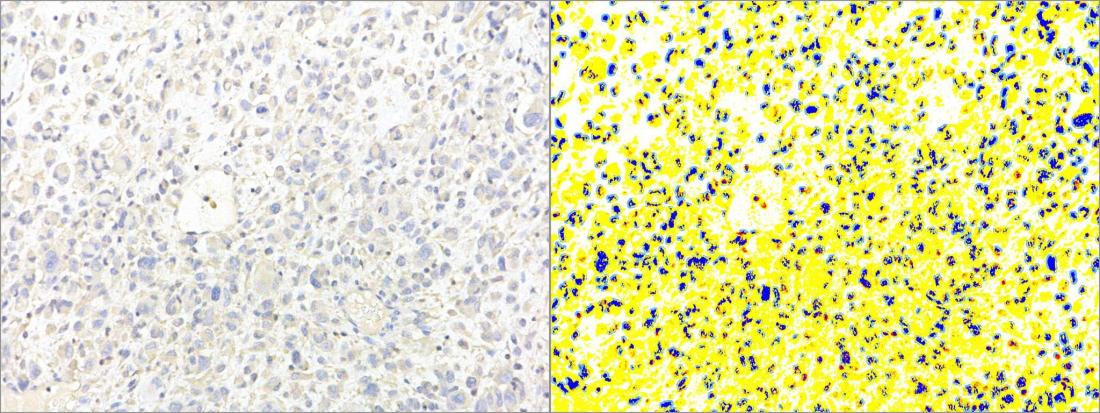


No.26 H-Score 136.3


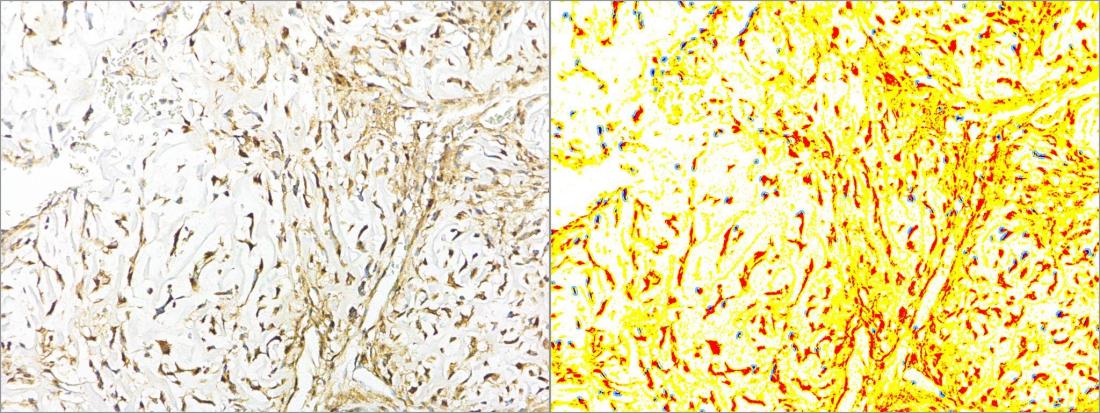


No.27 H-Score 107.056


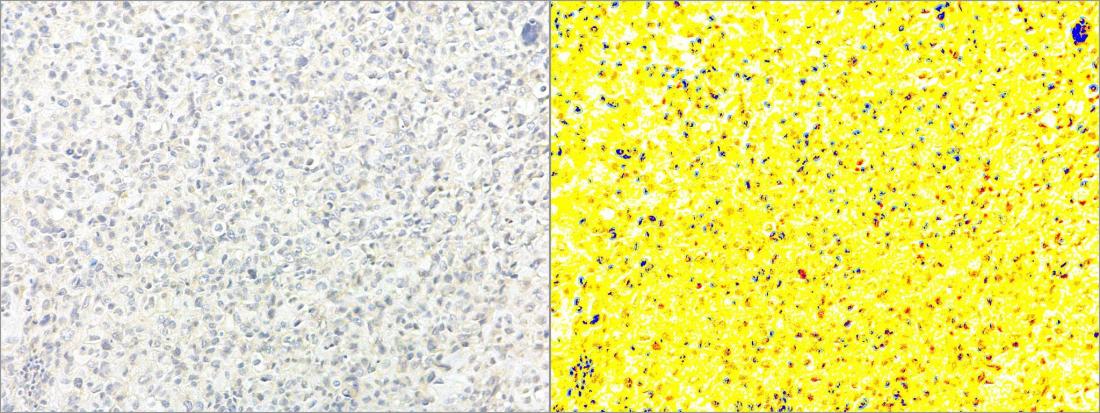


No.28 H-Score 71.4


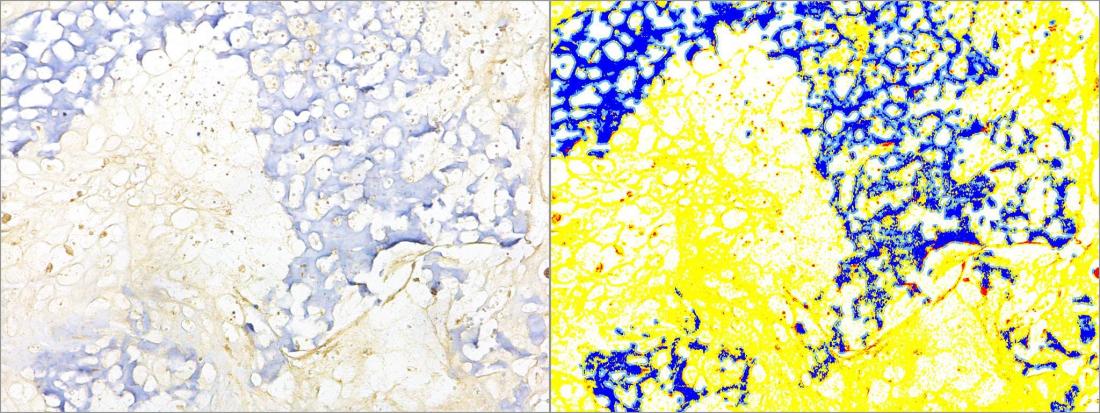


No.29 H-Score 109.334


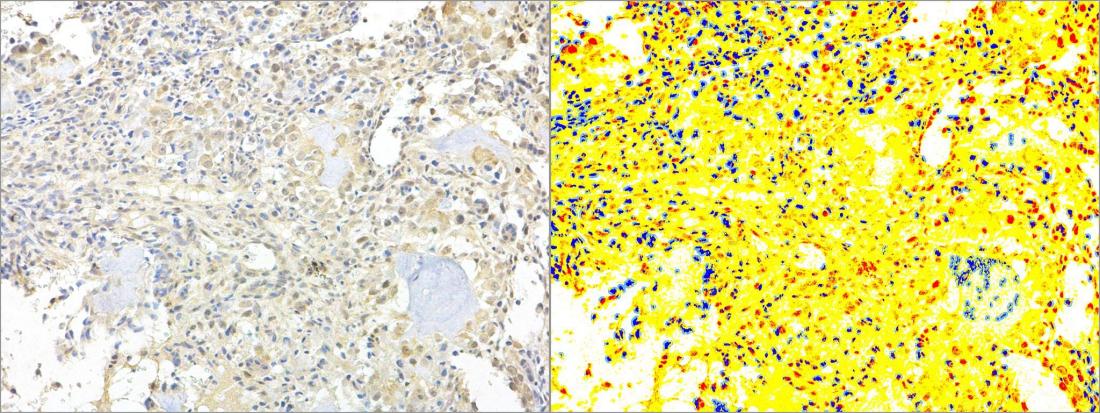


No.30 H-Score 113.368


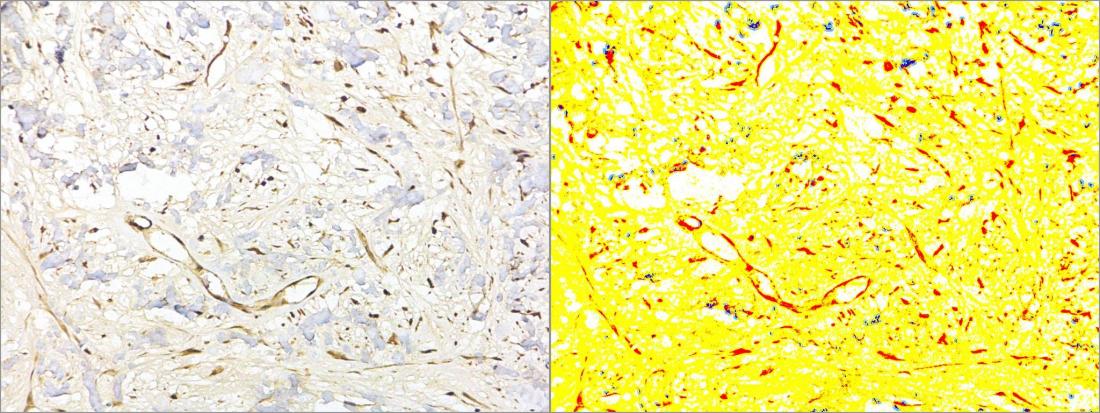


No.31 H-Score 28.113


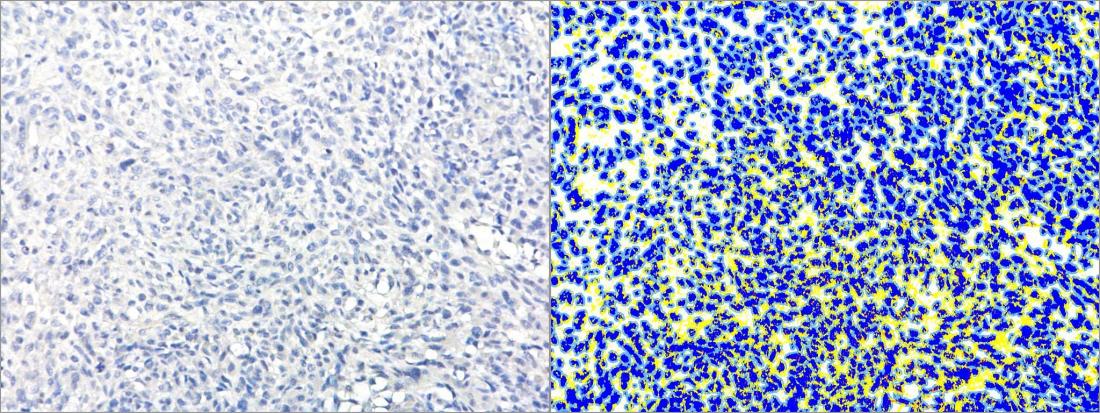


No.32 H-Score 59.646


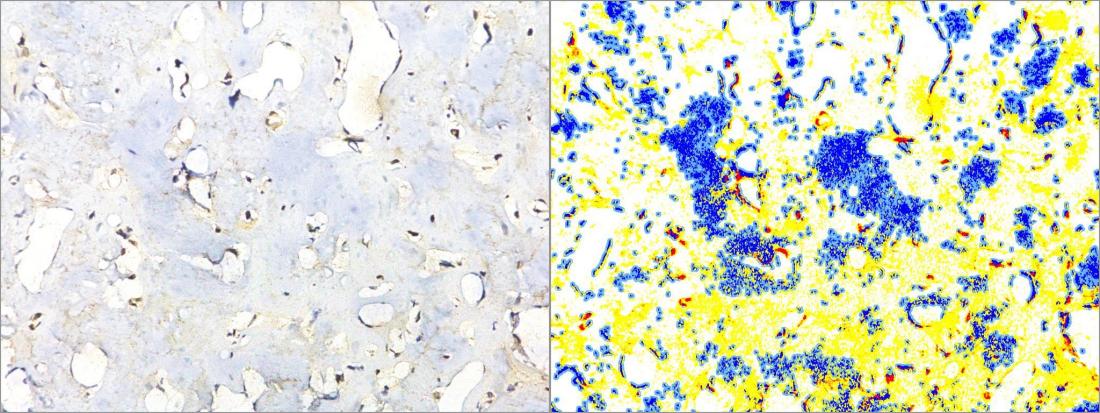


No.33 H-Score 83.623


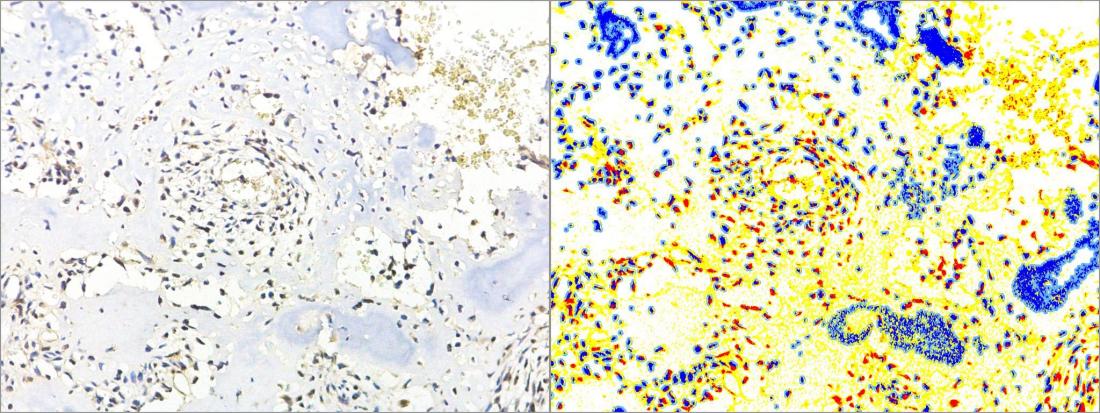


No.34 H-Score 21.879


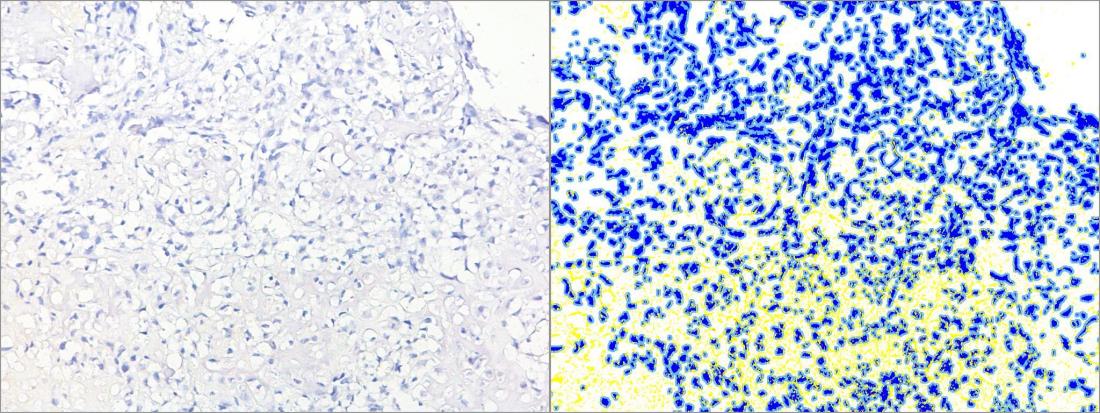


No.35 H-Score 94.679


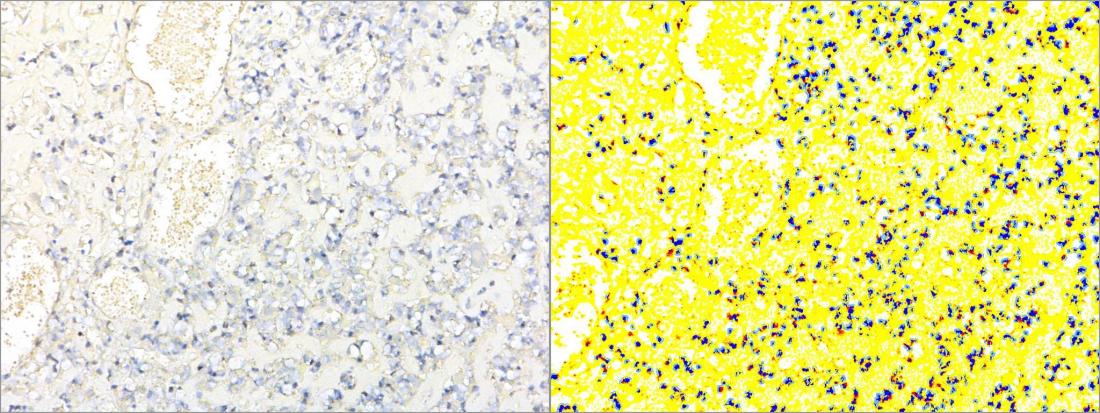


No.36 H-Score 23.276


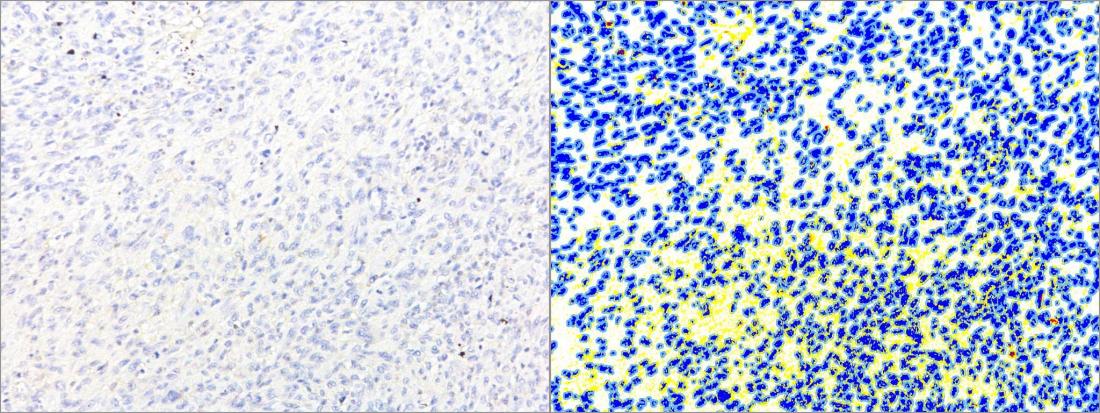


No.37 H-Score 103.148


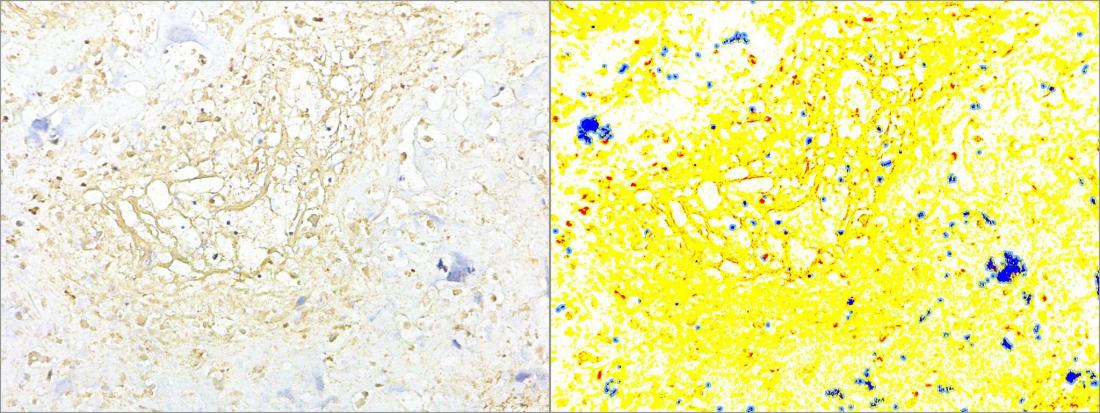


No.38 H-Score 108.128


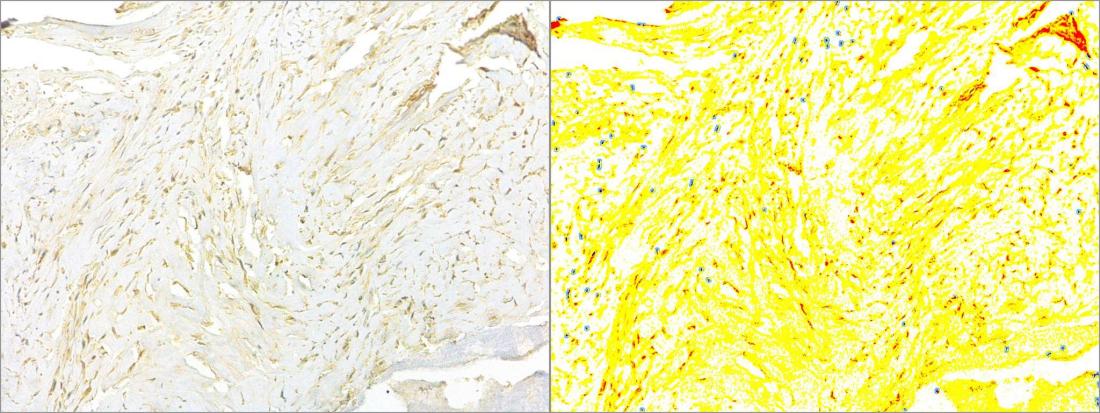


No.39 H-Score 109.617


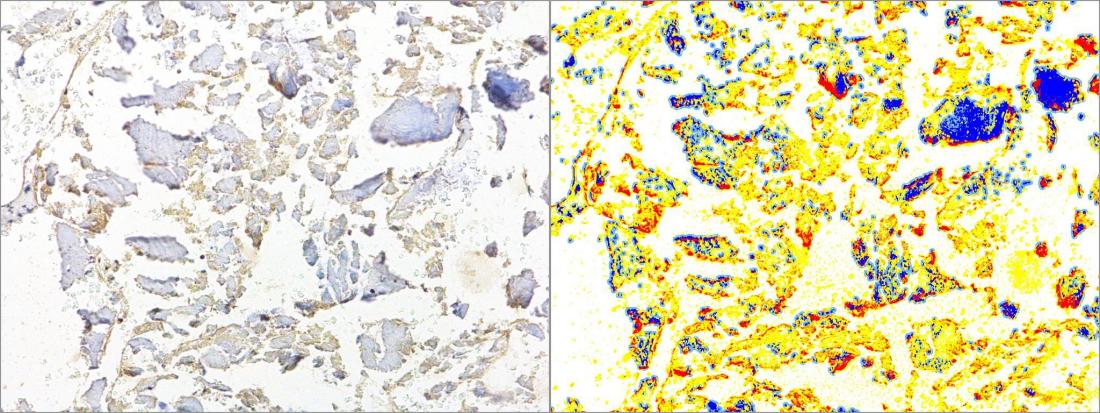


No.40 H-Score 107.831


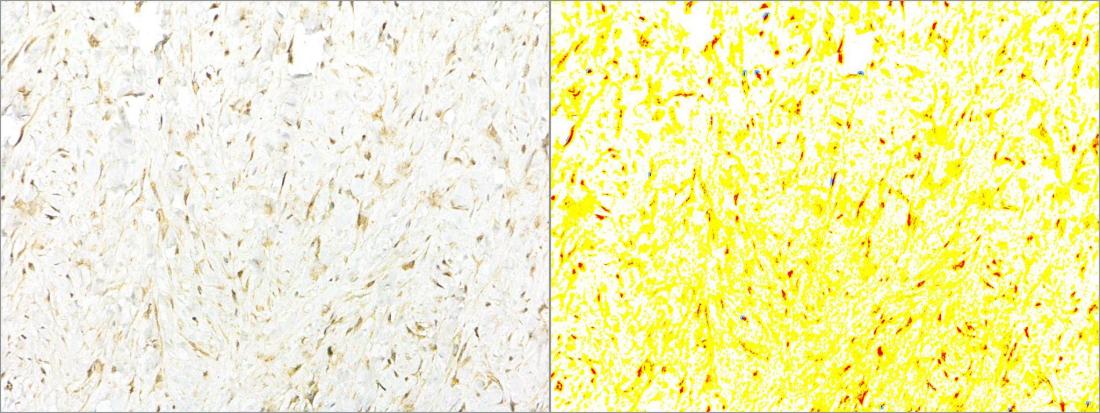


No.41 H-Score 113.125


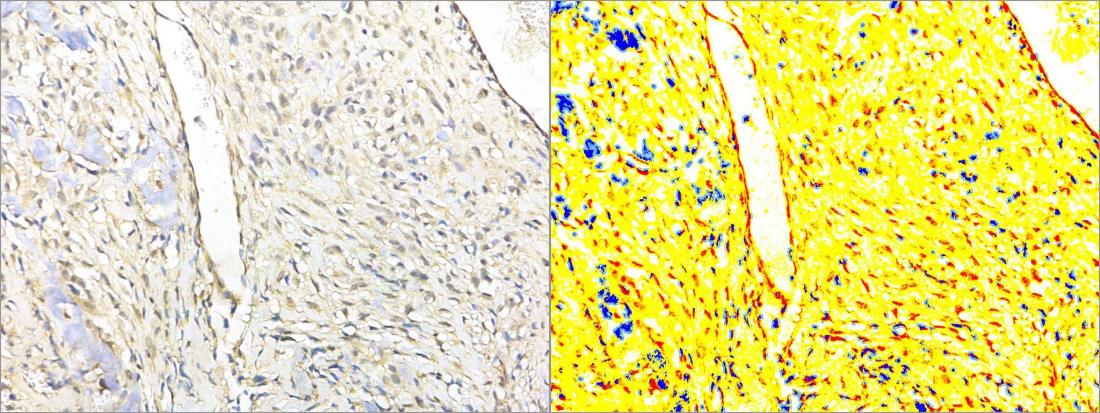


No.42 H-Score 105.717


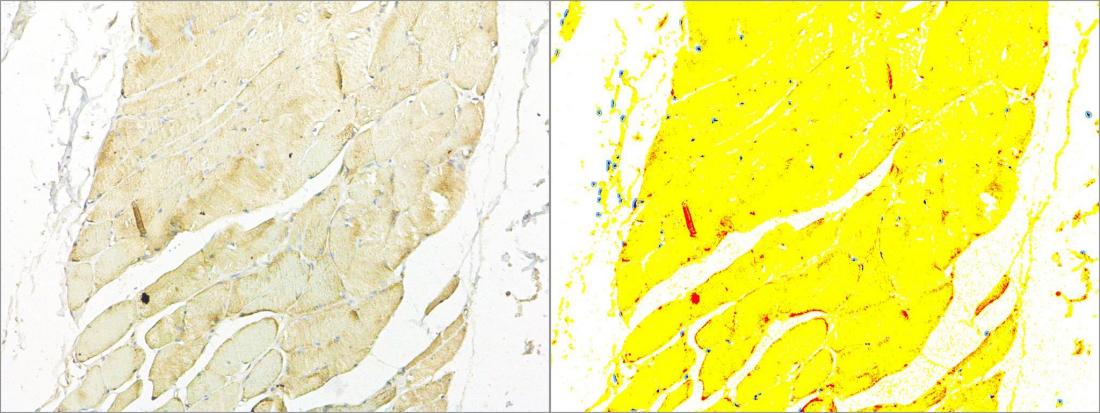


No.43 H-Score 116.362


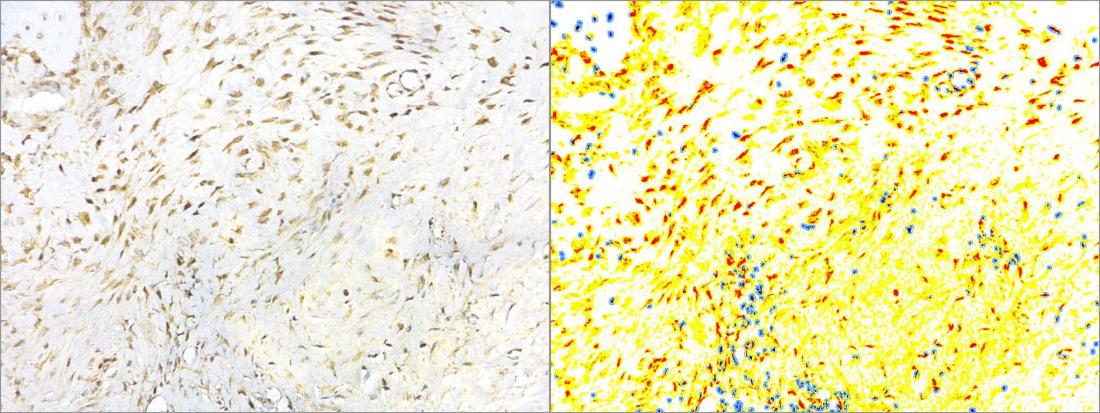


No.44 H-Score 120.514


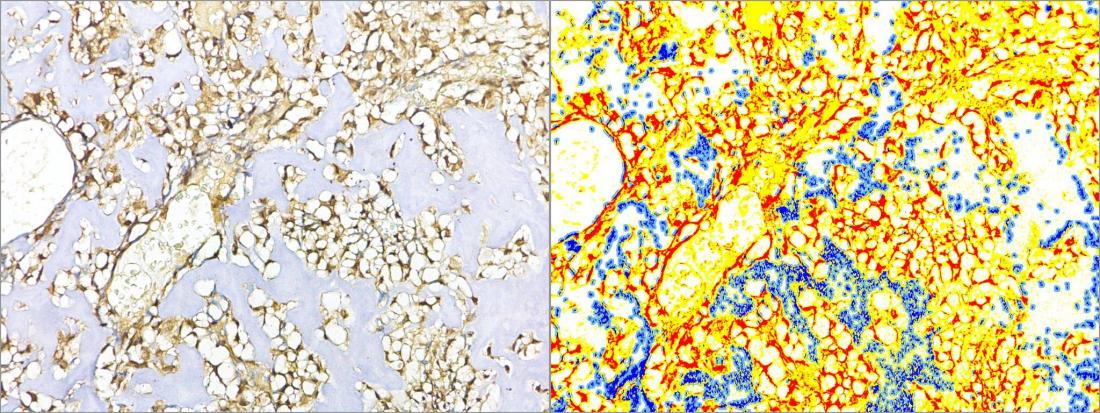


No.45 H-Score 117.144


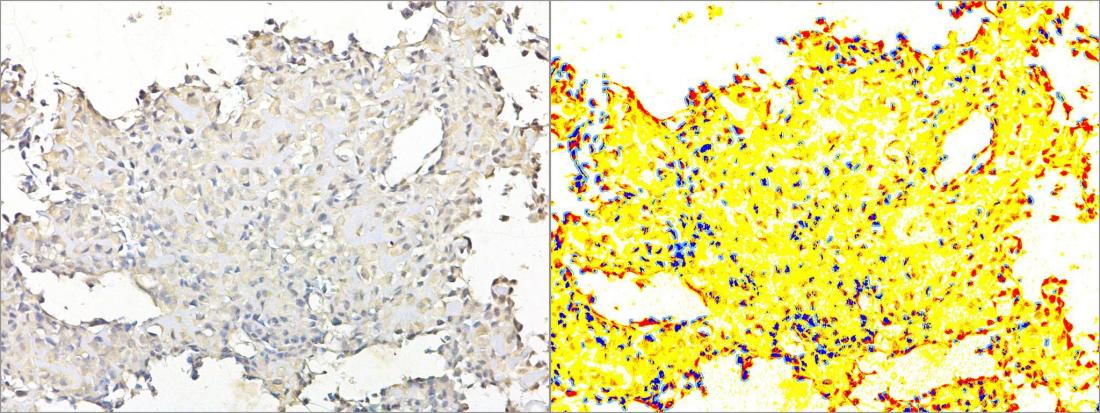


No.46 H-Score 77.567


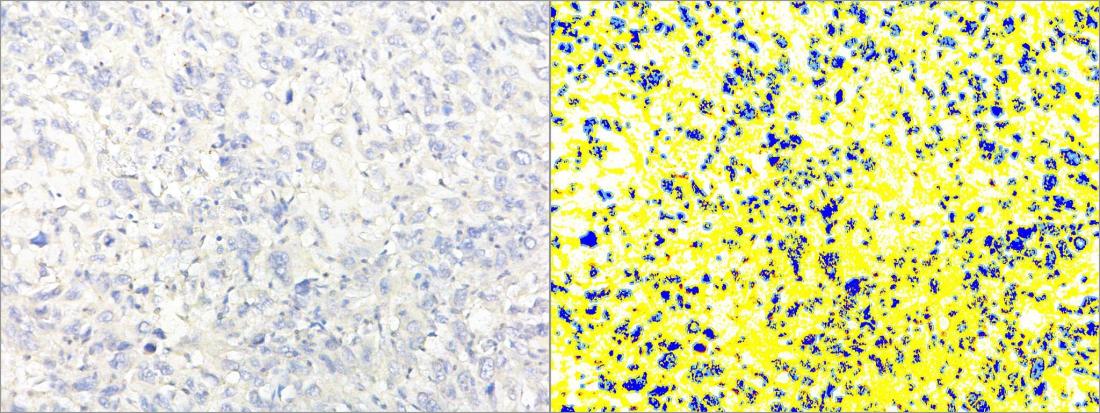


No.47 H-Score 59.043


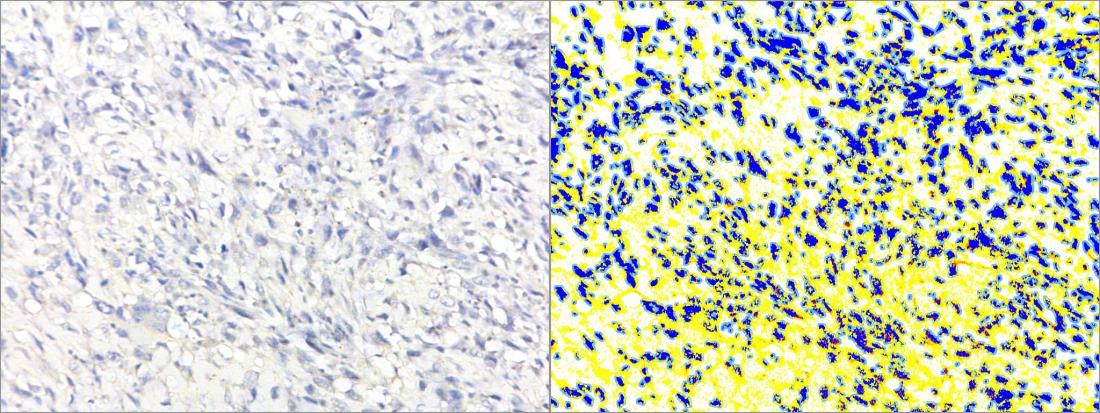


No.48 H-Score 119.066


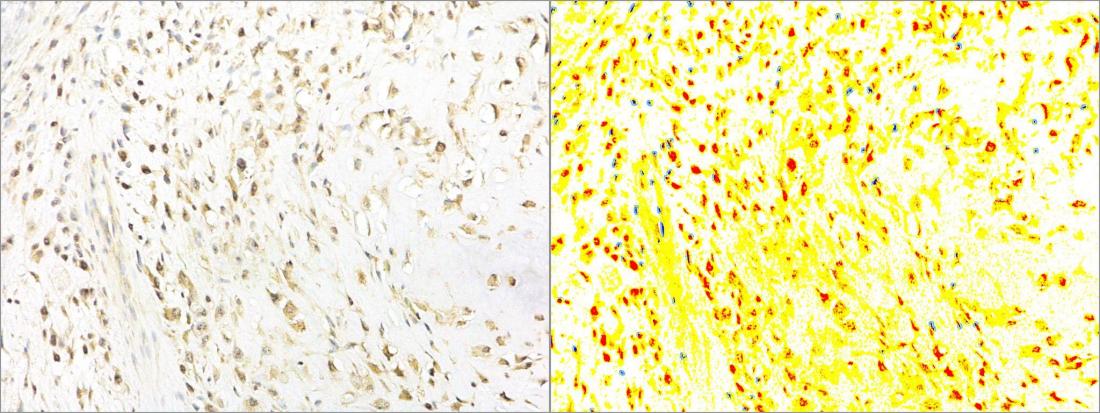


No.49 H-Score 138.214


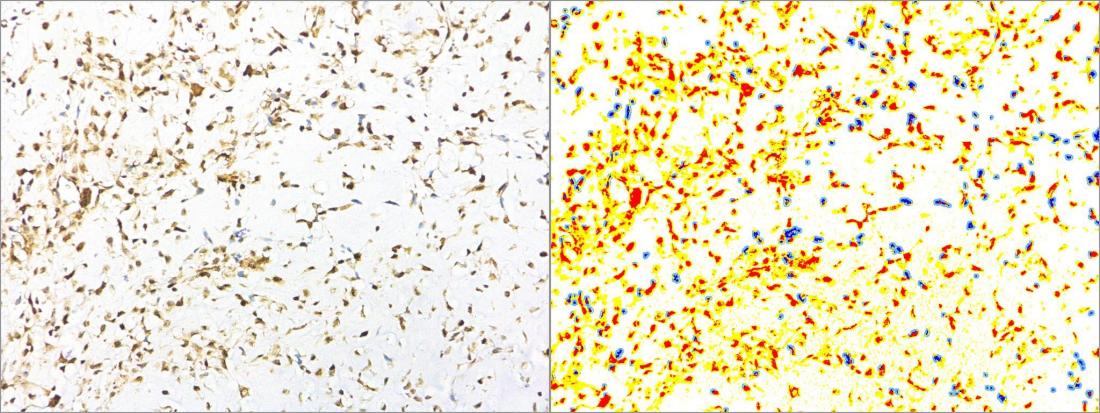


No.50 H-Score 106.893


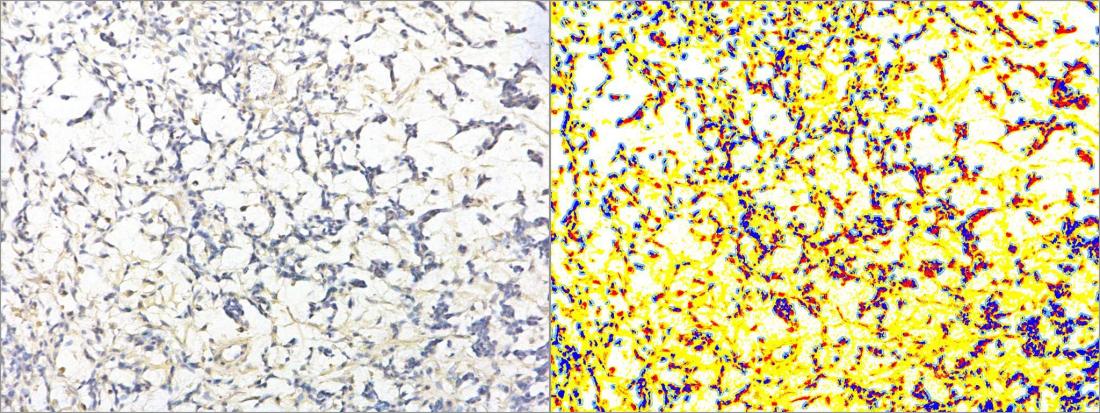


No.51 H-Score 119.697


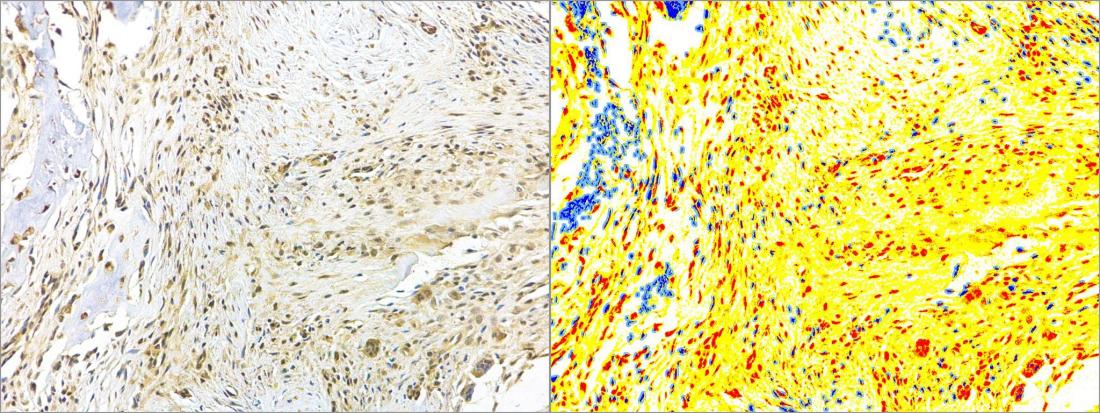


No.52 H-Score 115.021


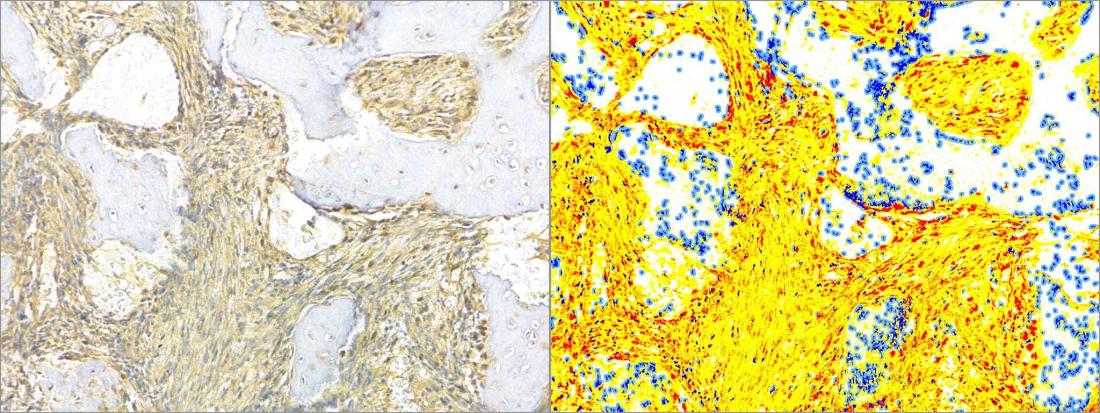


No.53 H-Score 102.394


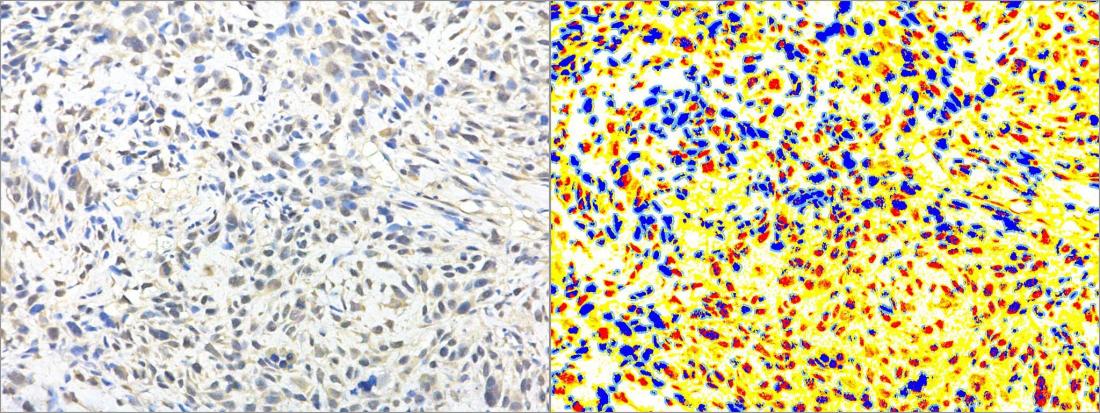


No.54 H-Score 14.062


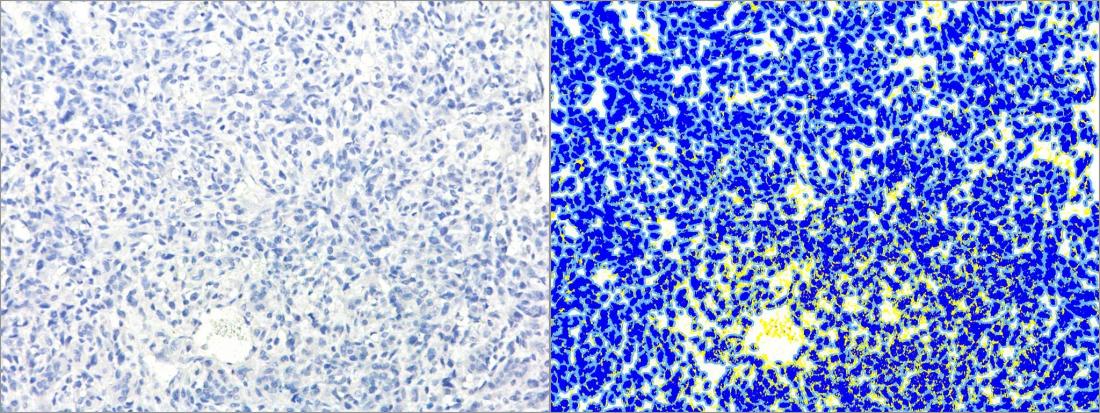


No.55 H-Score 91.601


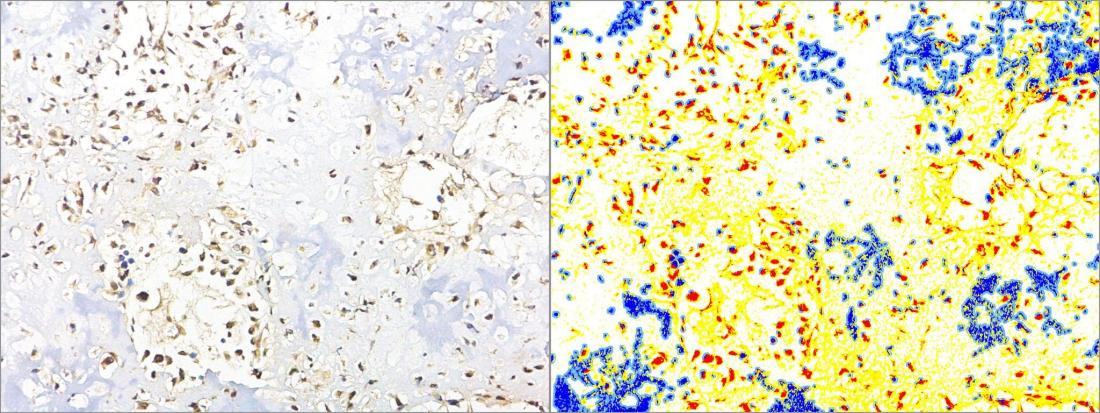


No.56 H-Score 124.29


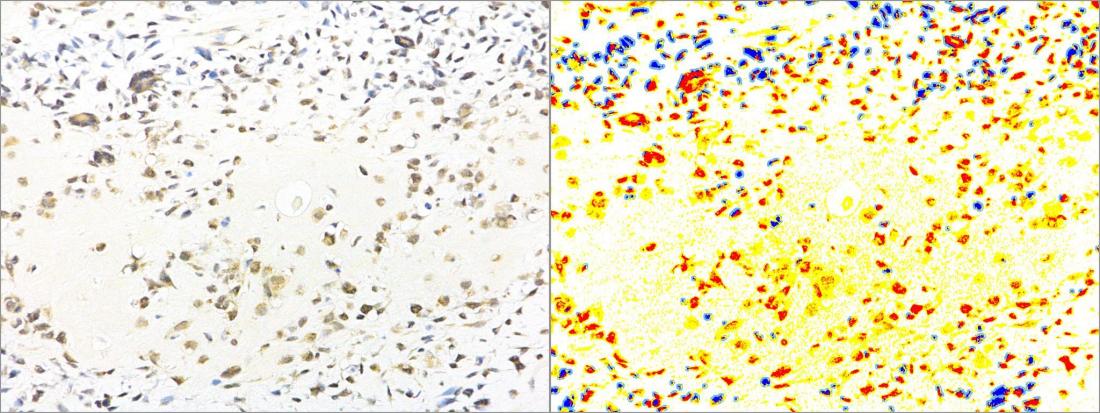


No.57 H-Score 112.619


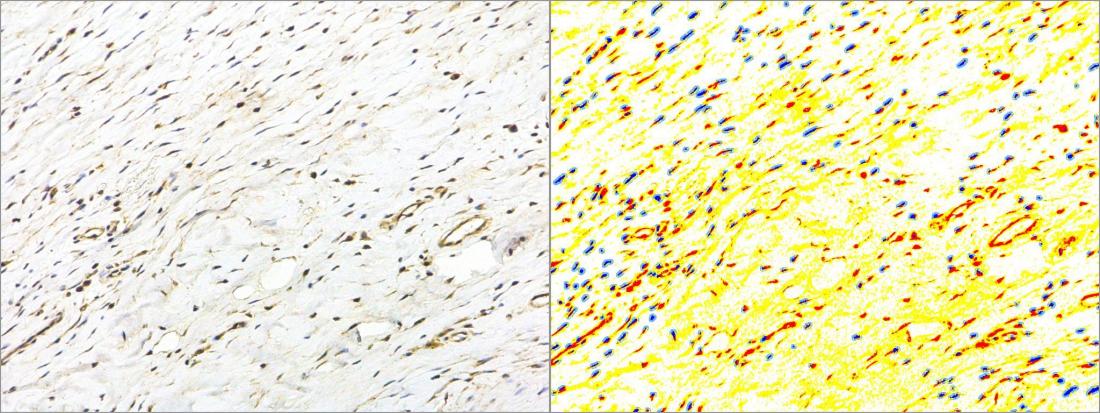


No.58 H-Score 148.15


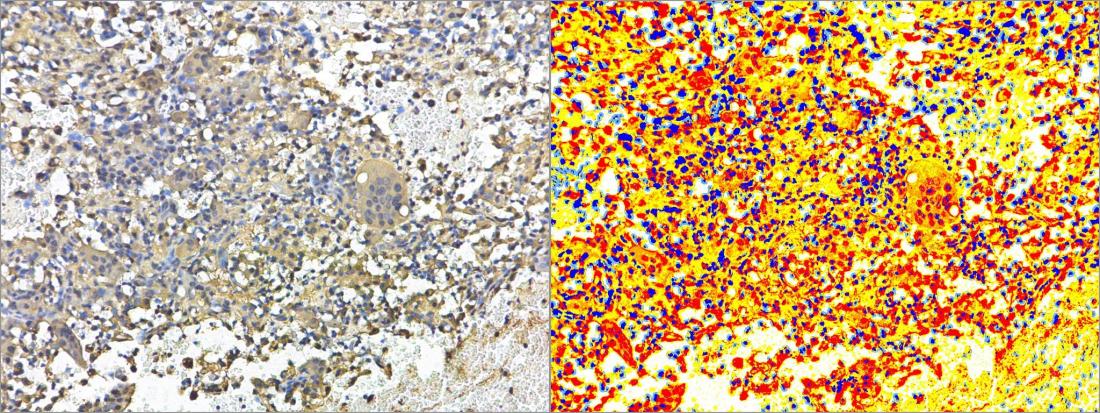


No.59 H-Score 52.152


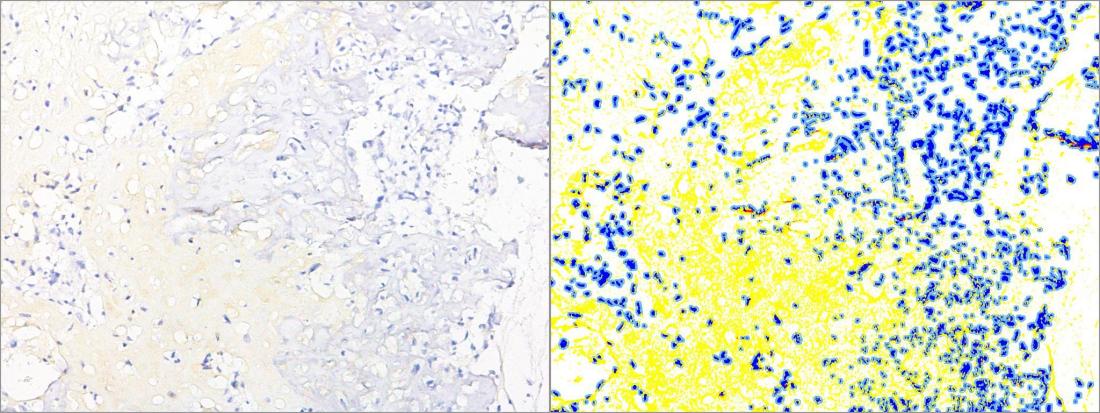


No.60 H-Score 89.453


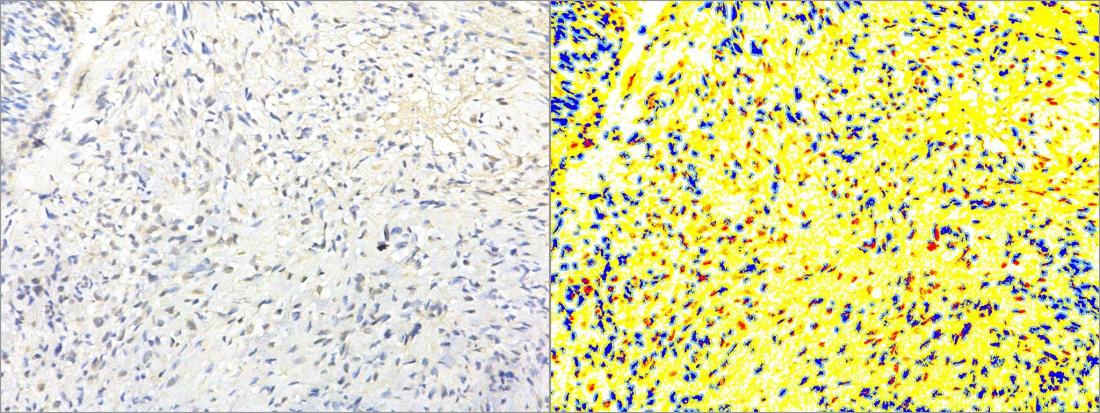


No.61 H-Score 60.872


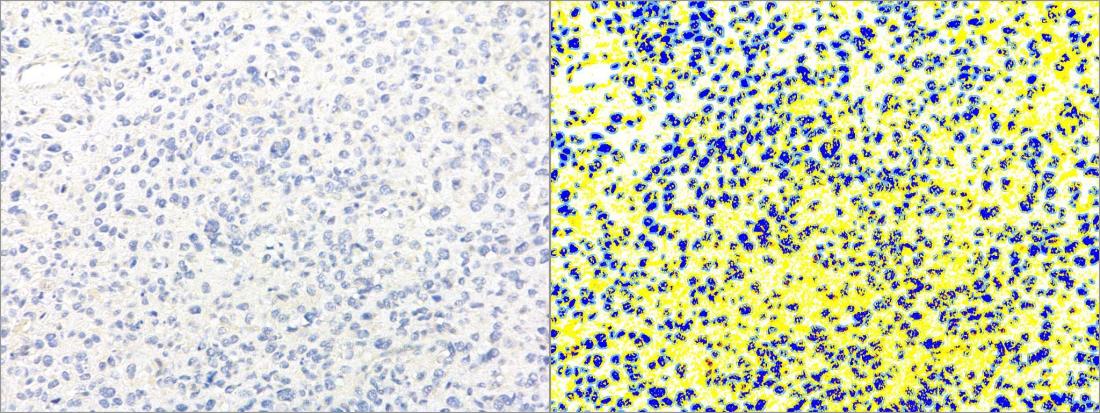


No.62 H-Score 108.488


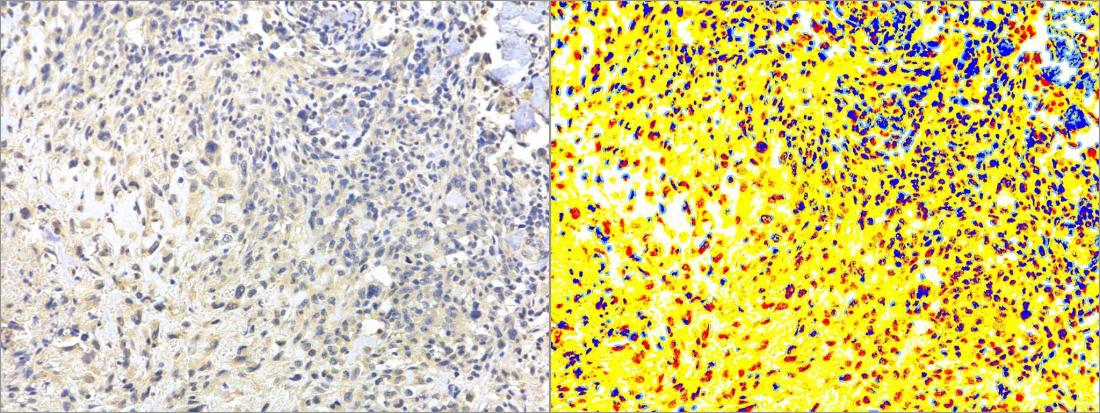


No.63 H-Score 117.719


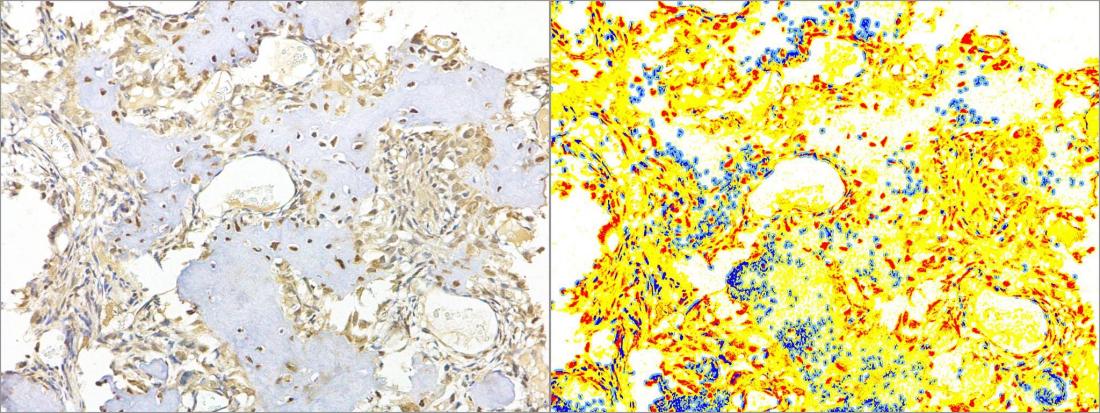


No.64 H-Score 37.341


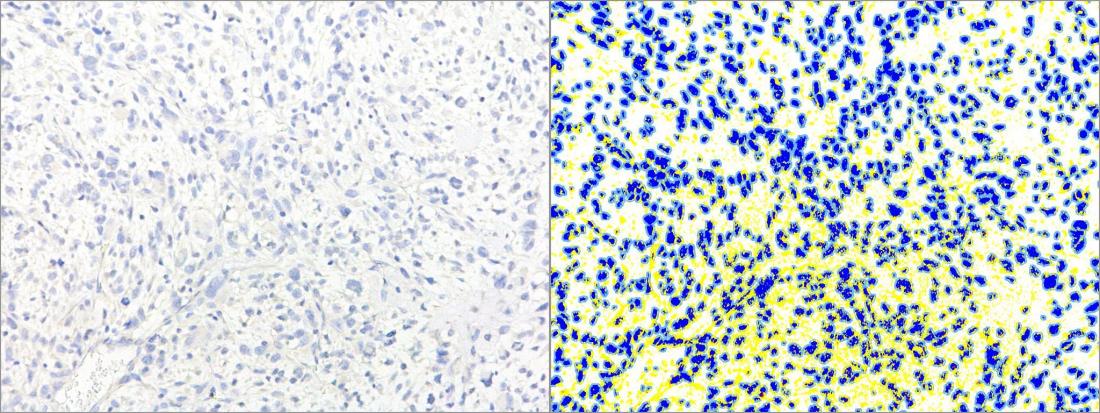


No.65 H-Score 91.484


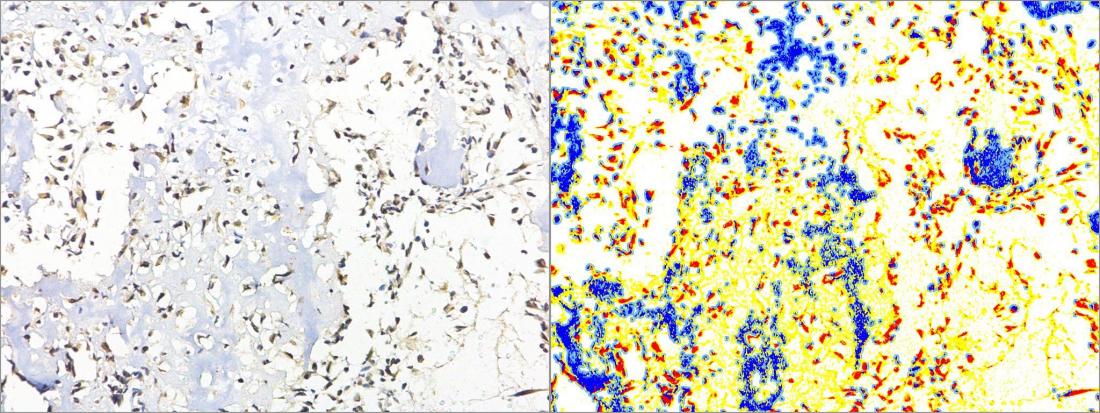


No.66 H-Score 125.739


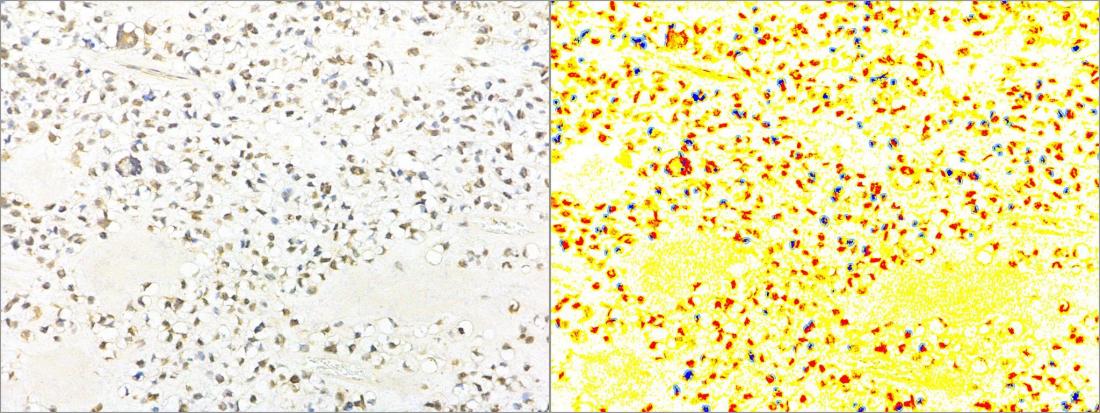


No.67 H-Score 106.638


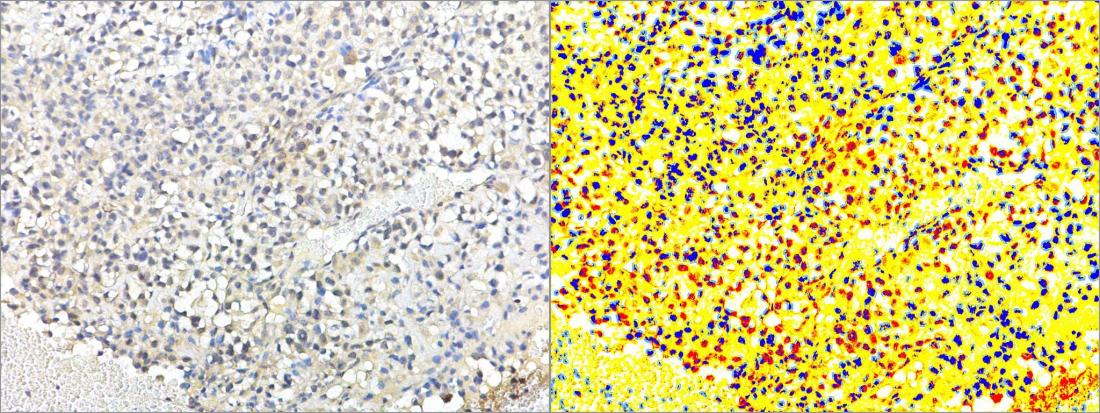


No.68 H-Score 101.473


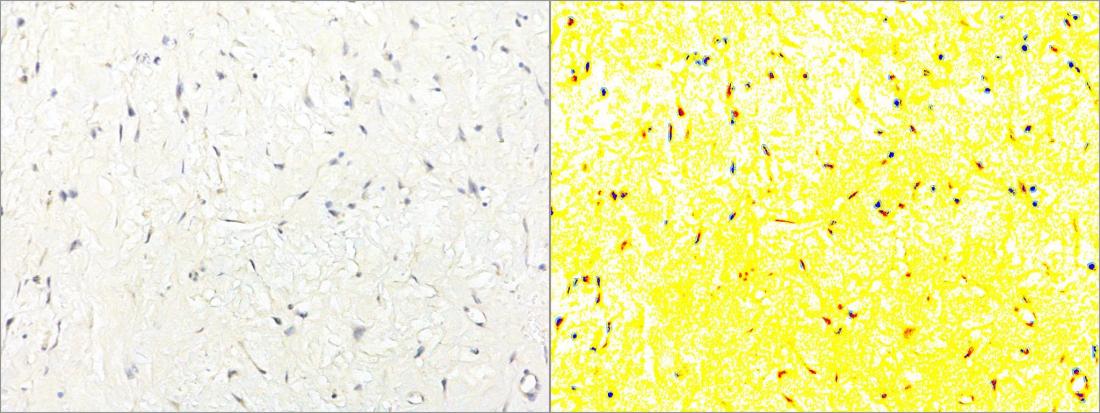


No.69 H-Score 107.621


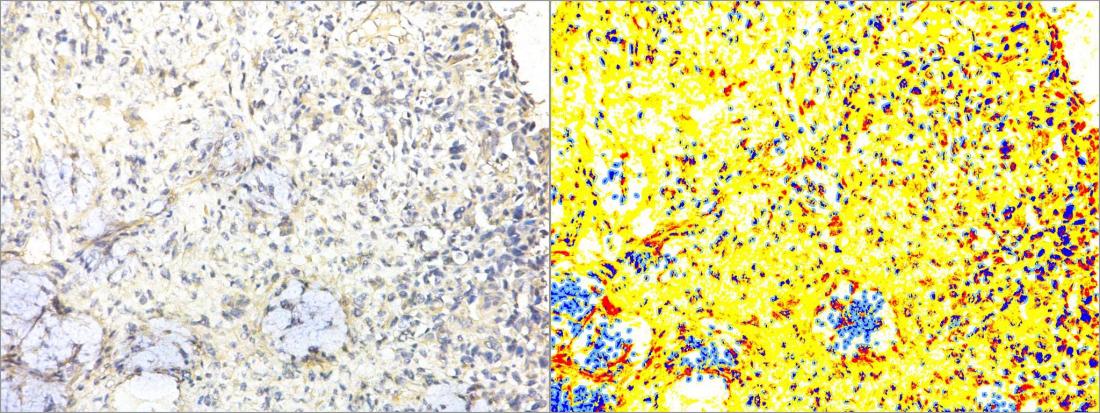


No.70 H-Score 98.071


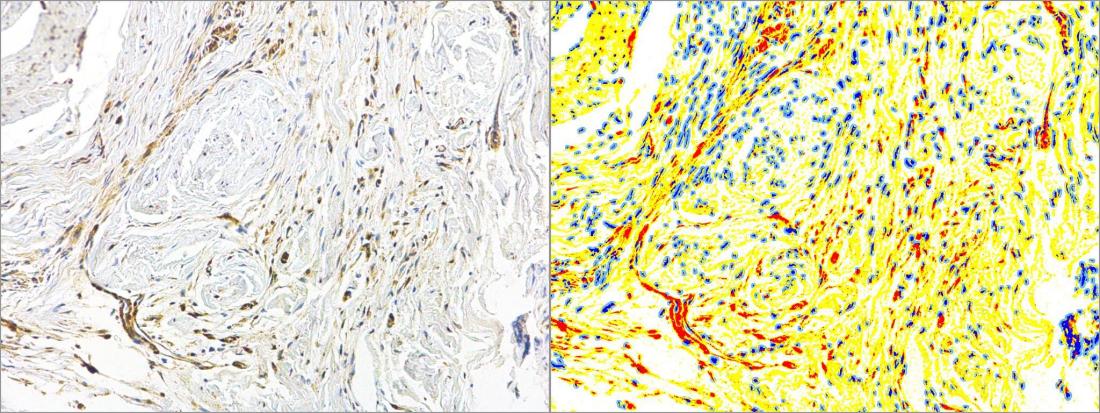


No.71 H-Score 121.354


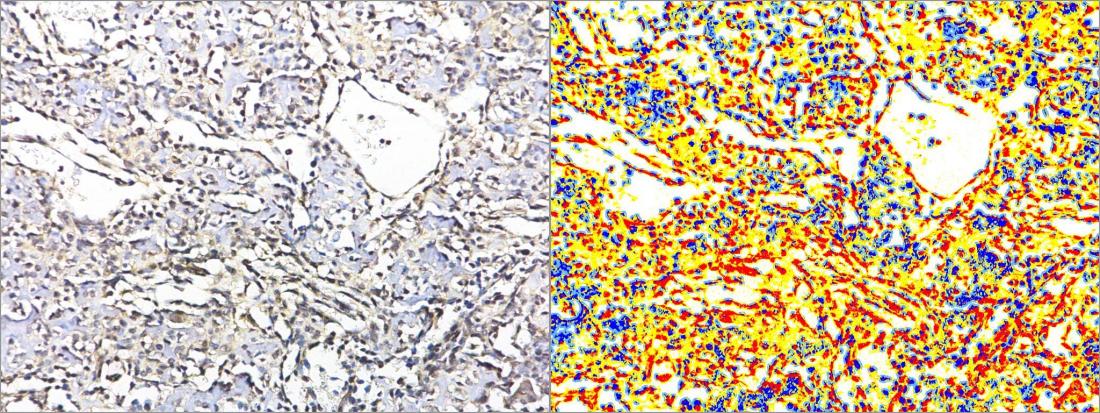


No.72 H-Score 108.727


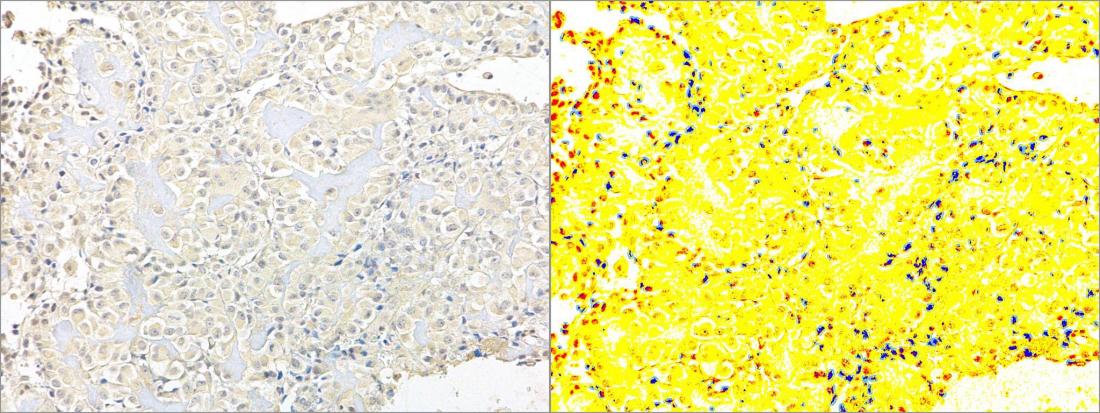


No.73 H-Score 94.282


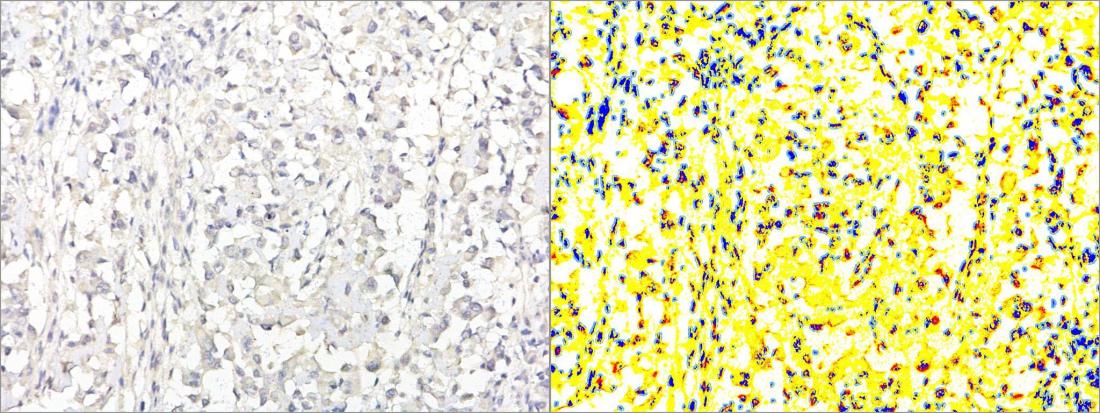


No.74 H-Score 107.803


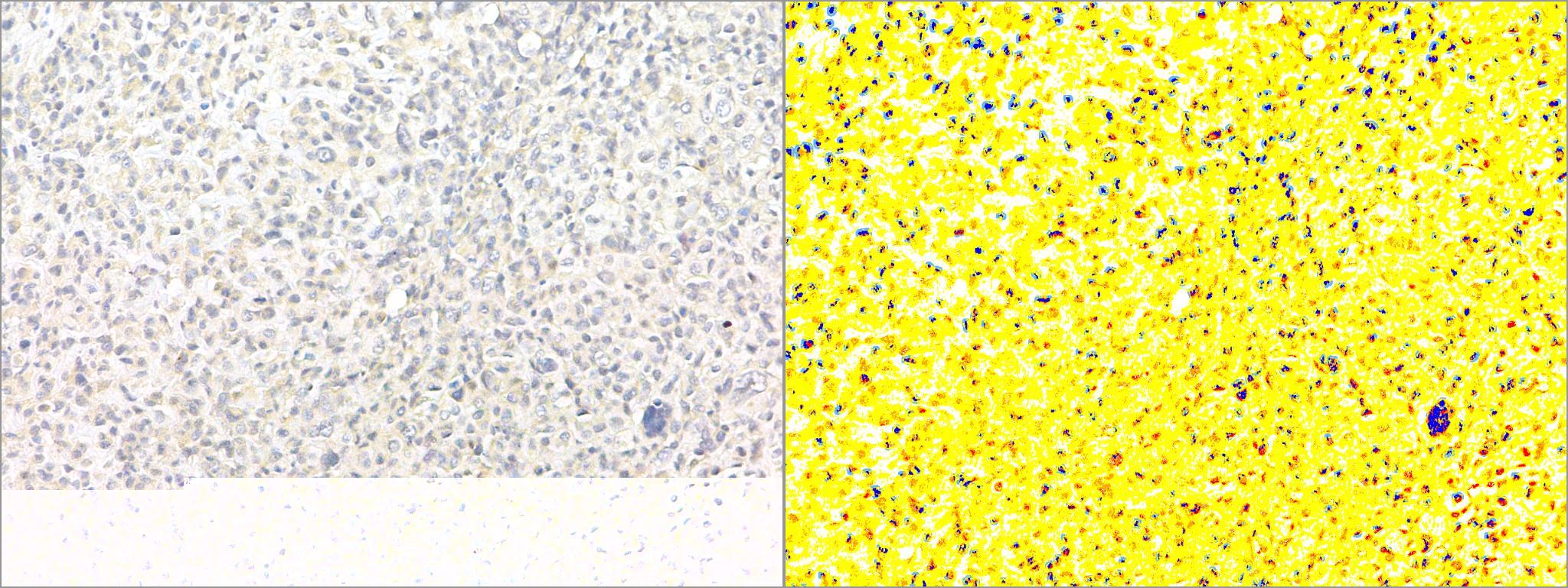


No.75 H-Score 133.487


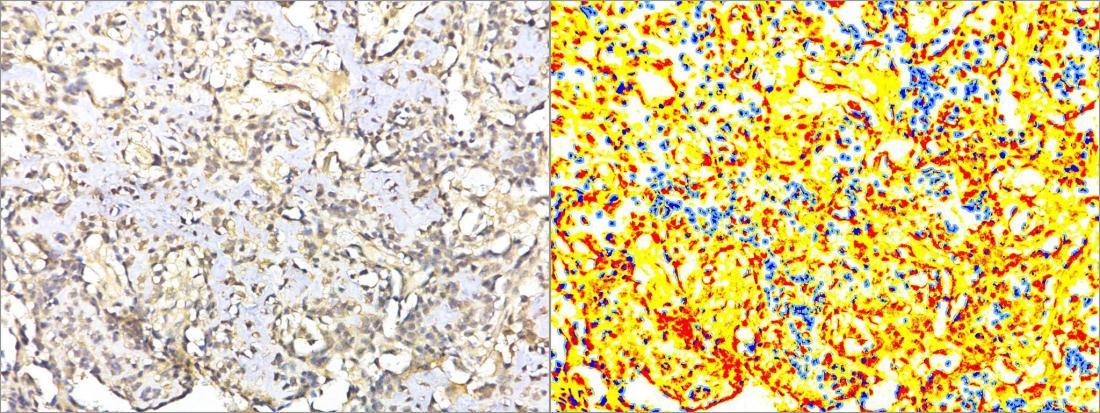


No.76 H-Score 120.388


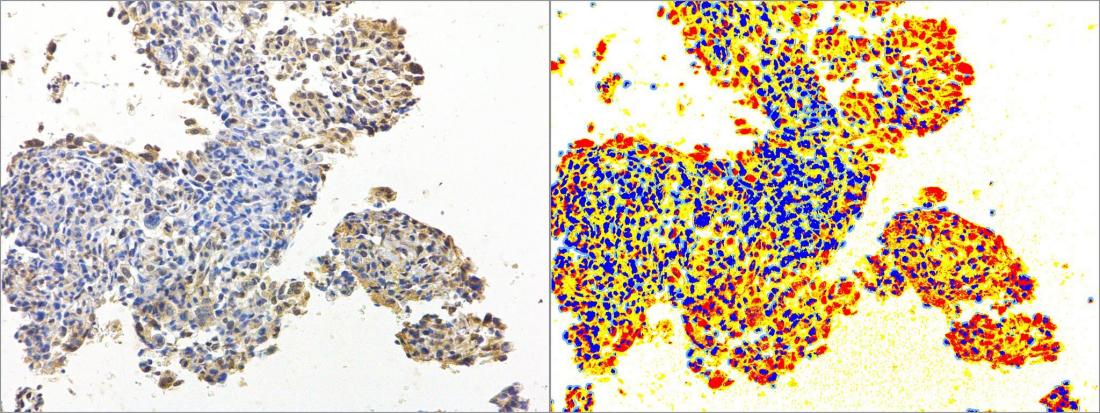


No.77 H-Score 114.44


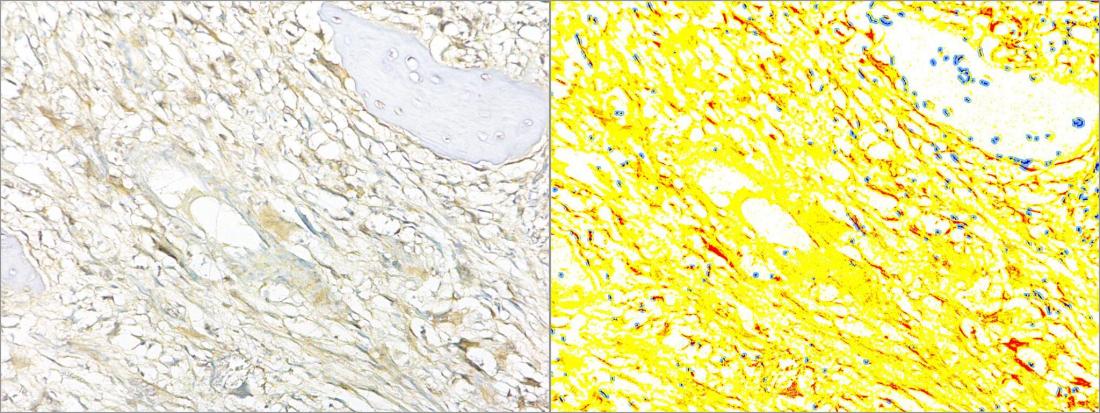


No.78 H-Score 90.405


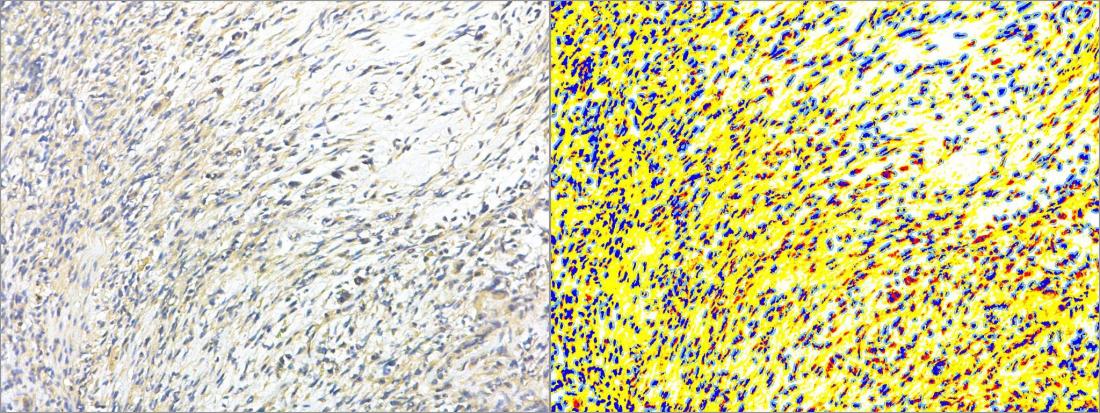

Supplement: Supplementary file 4 [file Table_3.docx]
